# IKAP—Identifying K mAjor cell Population groups in single-cell RNA-sequencing analysis

**DOI:** 10.1093/gigascience/giz121

**Published:** 2019-10-01

**Authors:** Yun-Ching Chen, Abhilash Suresh, Chingiz Underbayev, Clare Sun, Komudi Singh, Fayaz Seifuddin, Adrian Wiestner, Mehdi Pirooznia

**Affiliations:** 1 Bioinformatics and Computational Biology Laboratory, National Heart, Lung, and Blood Institute, National Institutes of Health, 12 South Drive, Bethesda, MD 20892, USA; 2 Hematology Branch, National Heart, Lung, and Blood Institute, National Institutes of Health, 10 Center Drive, Bethesda, MD 20814, USA

**Keywords:** single-cell RNA-sequencing, clustering, cell ontology, Seurat

## Abstract

**Background:**

In single-cell RNA-sequencing analysis, clustering cells into groups and differentiating cell groups by differentially expressed (DE) genes are 2 separate steps for investigating cell identity. However, the ability to differentiate between cell groups could be affected by clustering. This interdependency often creates a bottleneck in the analysis pipeline, requiring researchers to repeat these 2 steps multiple times by setting different clustering parameters to identify a set of cell groups that are more differentiated and biologically relevant.

**Findings:**

To accelerate this process, we have developed IKAP—an algorithm to identify major cell groups and improve differentiating cell groups by systematically tuning parameters for clustering. We demonstrate that, with default parameters, IKAP successfully identifies major cell types such as T cells, B cells, natural killer cells, and monocytes in 2 peripheral blood mononuclear cell datasets and recovers major cell types in a previously published mouse cortex dataset. These major cell groups identified by IKAP present more distinguishing DE genes compared with cell groups generated by different combinations of clustering parameters. We further show that cell subtypes can be identified by recursively applying IKAP within identified major cell types, thereby delineating cell identities in a multi-layered ontology.

**Conclusions:**

By tuning the clustering parameters to identify major cell groups, IKAP greatly improves the automation of single-cell RNA-sequencing analysis to produce distinguishing DE genes and refine cell ontology using single-cell RNA-sequencing data.

## Findings

Single-cell RNA-sequencing (scRNA-seq) enables inquiry of cell identity based on single-cell transcriptomics. To facilitate cell type characterization and recognition, computational methods have been developed for (i) clustering cells with similar transcriptomic profiles into groups and (ii) identifying a set of differentially expressed (DE) genes to differentiate those cell groups [1]. These 2 tasks are frequently treated as independent entities. However, groups identified by clustering greatly determine the DE genes associated with each group. Compared with clustering, computing DE genes is often more resource intensive. We therefore attempted to improve and accelerate biological interpretation of RNA-seq data by developing an algorithm to effectively identify the *k* major groups that produce distinguishing DE genes.

Despite the existence of well-performing scRNA-seq clustering methods, identifying *k* groups remains a challenge owing to parameter specification [2]. Most (if not all) clustering methods require a parameter suggesting *k* and a list of genes or principal components (PCs) for computing cell-to-cell similarity. The proper *k* is generally unknown a priori. Choosing a small *k* may mix >1 cell type in a group whereas choosing a large *k* would result in many subgroups of unclear biological significance. Both can complicate cell type recognition by producing uninformative DE genes. In addition, feature selection can affect grouping quality, which, in turn affects its distinguishing power. Therefore, *k* and feature selection often become a bottleneck in the scRNA-seq analysis pipeline.

**Figure 1:**
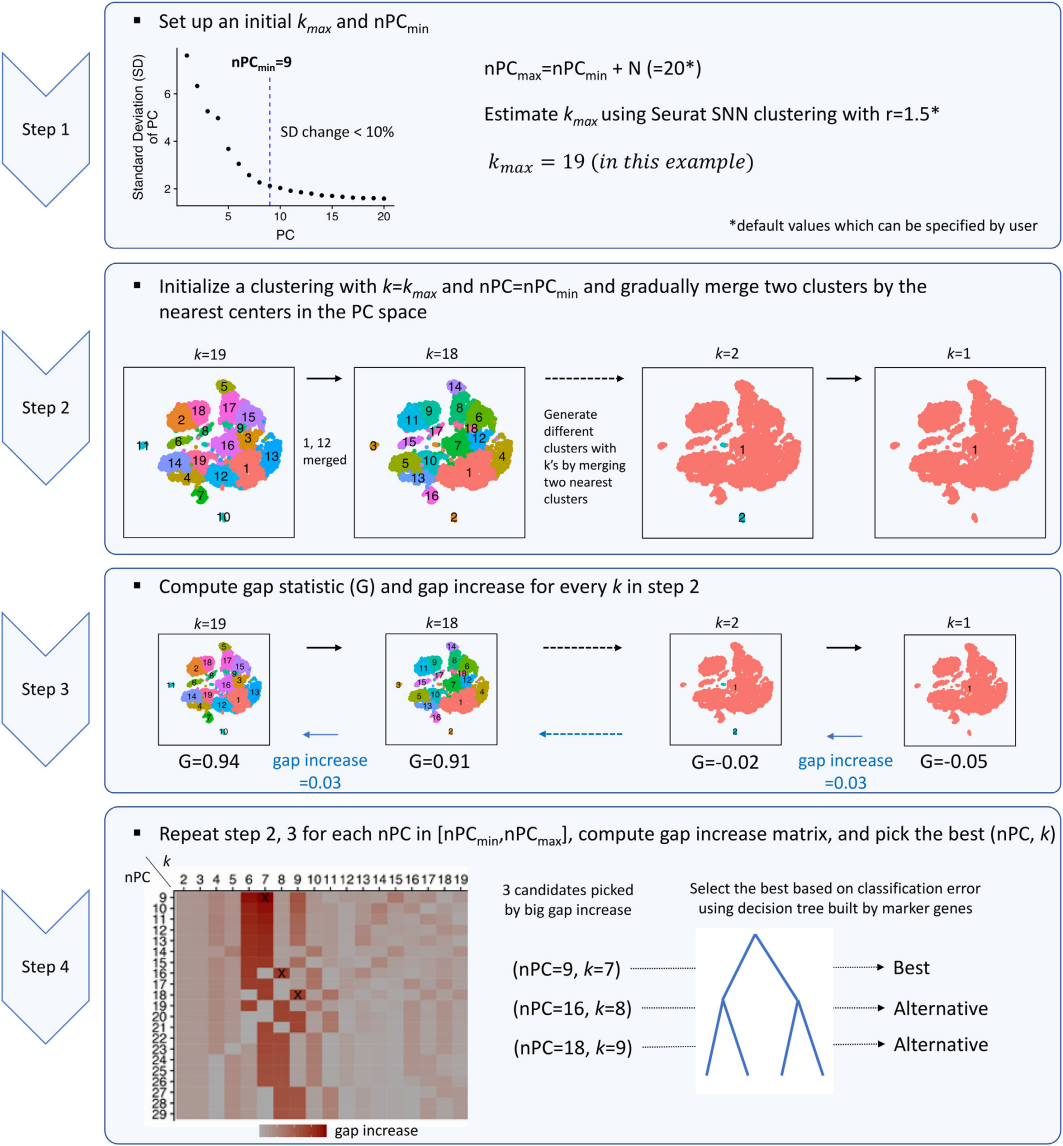
IKAP workflow. See Online Methods for details. SNN: shared nearest neighbor.

To address this issue, we propose an unbiased approach—called IKAP (Identifying K mAjor cell Population groups)—which identifies well-separated *k* major groups poised to produce distinguishing DE genes in an scRNA-seq dataset by systematically exploring the parameter space (Fig. 1 and Online Methods). IKAP is implemented on top of Seurat [3]—one of the most widely used scRNA-seq analysis packages—in which clustering requires 2 parameters that need to be specified by users: resolution *r* that determines *k* (the higher *r*, the larger *k*) and the number of top principal components (nPC). Briefly, for a given nPC, IKAP initializes a set of *k*_max_ groups by setting a high *r*. To simulate the fine-to-coarse grouping process, 2 nearest groups are merged iteratively, generating *k*_max_ sets of groups with *k* = 1 to *k*_max_. For each set, the gap statistic is computed to measure the gap between the grouping with observed data and that with random data [4]. The gap often monotonically increases (at variable amount) as *k* increases from 1 to *k*_max_, indicating that splitting out each group somewhat contributes to the grouping moving away from randomness (Supplementary Fig. 1). We reason that those *k*’s that contribute more (i.e., yield large gap increase) might correspond to the set of *k* well-separated major groups. IKAP repeats this procedure for a range of nPCs. Finally, a few candidate sets of cell groups with large gap increases are picked. Among all candidate sets, the one with the lowest classification error is marked as the best, using decision trees built from DE genes. IKAP can be run default without specifying any parameter as we did for experimentation in this study and can potentially be tailored for scRNA-seq clustering methods other than Seurat.

**Figure 2:**
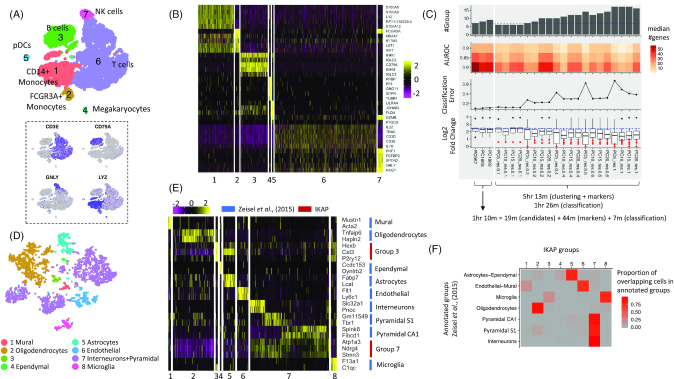
Major cell groups identified for PBMC_8K (A, B, and C) and the mouse cortex dataset (D, E, and F). (A) Shown are tSNE plots for the 7 major groups identified by IKAP with cell types labeled (top) and expression of known marker genes (bottom): *CD3E* for T cells, *CD79A* for B cells, *GNLY* for NK cells, and *LYZ* for monocytes. (B) The heat map for expression of the top 5 DE genes (by expression fold change) from each group in (A). Rows are genes and columns are cells. (C) Performance summary of 3 candidate sets proposed by IKAP (left) and the 20 trial sets (right). Note that the number of candidate sets can vary for different datasets. Running time is shown at the bottom. The dashed blue lines indicate the number of cell groups (top) and the median log_2_ fold change (bottom) of the best set (PC9K7). (D) The tSNE plot for 8 major groups identified by IKAP in the mouse cortex dataset consistent with previously annotated cell types. (E) The heat map for expression of marker genes annotated for major cell types in Zeisel et al. 2015 [6] (blue) and DE genes identified by IKAP for groups 3 and 7 in (D) (red). (F) The heat map indicates the proportion of overlapping cells between IKAP-identified major groups and major cell types annotated in Zeisel et al. 2015 [6].

We tested IKAP on a peripheral blood mononuclear cell (PBMC) dataset of ∼8,000 cells (denoted as PBMC_8K) from a healthy donor [5]. The best set (with *k* = 7 and nPC = 9; thus, abbreviated as PC9K7) and 2 alternative sets (PC16K8 and PC18K9, respectively) were reported. The major groups reported in PC9K7 were effectively aligned with known major cell types such as B cells, T cells, and natural killer (NK) cells as evidenced by expression of marker genes (Fig. 2A). Note that in this paper, cell types are defined as types of cells that have been manually (or conventionally) annotated and defined by a set of marker genes. Those marker genes of different cell types (such as *CD3E, TRAC*, and *IL32* for T cells) were also prioritized to the top of the DE gene list for every group (Fig. 2B), facilitating cell type determination. To compare with the trial-and-error strategy, we varied nPC (=5, 10, 15, and 20) and *r* (=0.1, 0.2, 0.4, 0.6, and 1.0) to generate 20 trial sets of groups using Seurat clustering. Most trial sets did not divide cells into major cell types (Supplementary Fig. 2) and cell type marker genes were not ranked at the top or were unspecific to particular cell groups, complicating cell type recognition (Supplementary Fig. 3). To quantitatively evaluate whether a set of cell groups can produce distinguishing DE genes, we designed 3 metrics: (i) the number of DE genes with high AUROC (area under the receiver operating curve), (ii) in-group versus out-of-group expression fold change among high-AUROC DE genes, and (iii) classification error when classifying cells using decision trees built from multiple DE genes. Compared with the 20 trial sets, we found PC9K7 yielded more DE genes with high AUROC, higher expression fold change, and lower classification error (Fig. 2C). Two alternative sets (PC16K8 and PC18K9) also agreed with major cell types and produced distinguishing DE genes with more rare types or subtypes reported (Fig. 2C; Supplementary Fig. 4). Finally, IKAP consumed less time (1 hr 10 m) than computing the 20 trial sets (5 hr 13 m) (Fig. 2C). Although IKAP required an extra step to explore parameter space (19 m), much time was saved because of fewer runs (3 candidate sets versus 20 trial sets) of time-consuming DE gene identification. The result shows that IKAP could help biological interpretation by picking appropriate parameters and reporting major cell groups that produce distinguishing DE genes within a reasonable time frame.

To test robustness, we repeated the same analysis for another dataset, PBMC_4K (∼4,000 cells), from the same donor [5]. This time 2 candidate sets (PC8K7 and PC20K8) were picked with PC20K8 marked as the best. Compared with the 20 trial sets, IKAP candidate sets were in better agreement with known cell types, cell type marker genes being prioritized at the top, producing more distinguishing DE genes, and consuming shorter running time (Supplementary Figs 5–7).

Next, we applied IKAP on a mouse cortex dataset (∼3,000 cells) in which 9 major cell types were previously annotated in Zeisel et al. 2015 [6] (Supplementary Fig. 8A). IKAP identified 1 candidate set with 8 major groups (PC13K8) (Fig. 2D). Six groups were consistent with the annotated cell types as evidenced by expressing marker genes annotated specific to those cell types (Fig. 2E) and high proportion of overlapping cells (Fig. 2F). For the 2 remaining groups, one was a subtype of microglia cells (group 3 in Fig. 2D) characterized by *Hexb* (AUROC = 1.0), *Cst3* (0.98), and *P2ry12* (0.98) and the other was the union of 3 annotated cell types, interneurons, pyramidal S1, and pyramidal CA1 (group 7 in Fig. 2D), characterized by many DE genes including *Atp1a3* (0.93), *Ndrg4* (0.94), and *Stmn3* (0.93) (Fig. 2E; Supplementary Fig. 9). Those genes were significantly upregulated in interneurons, pyramidal S1, and pyramidal CA1 compared with every other cell type annotated in Zeisel et al. 2015 [6] (Supplementary Table 1). The common gene expression profile suggested a high-level cell identity shared across the 3 cell types, which was consistent with the clustering result showing their similarity compared to other cell types in Zeisel et al. 2015 (see Fig. 1C in [6]). Compared with the 20 trial sets (generated in the same way described above), DE genes in PC13K8 yielded higher expression fold change and lower classification error (Supplementary Fig. 8B). Interestingly, the number of DE genes with high AUROC remained high as more subgroups were identified (which was not true for PBMC datasets), suggesting that transcriptomic differentiation in the mouse cortex cells was very fine-grained. Overall, IKAP successfully recovered major cell types (rather than many subtypes) that were consistent with previous annotations and produced distinguishing DE genes.

**Figure 3:**
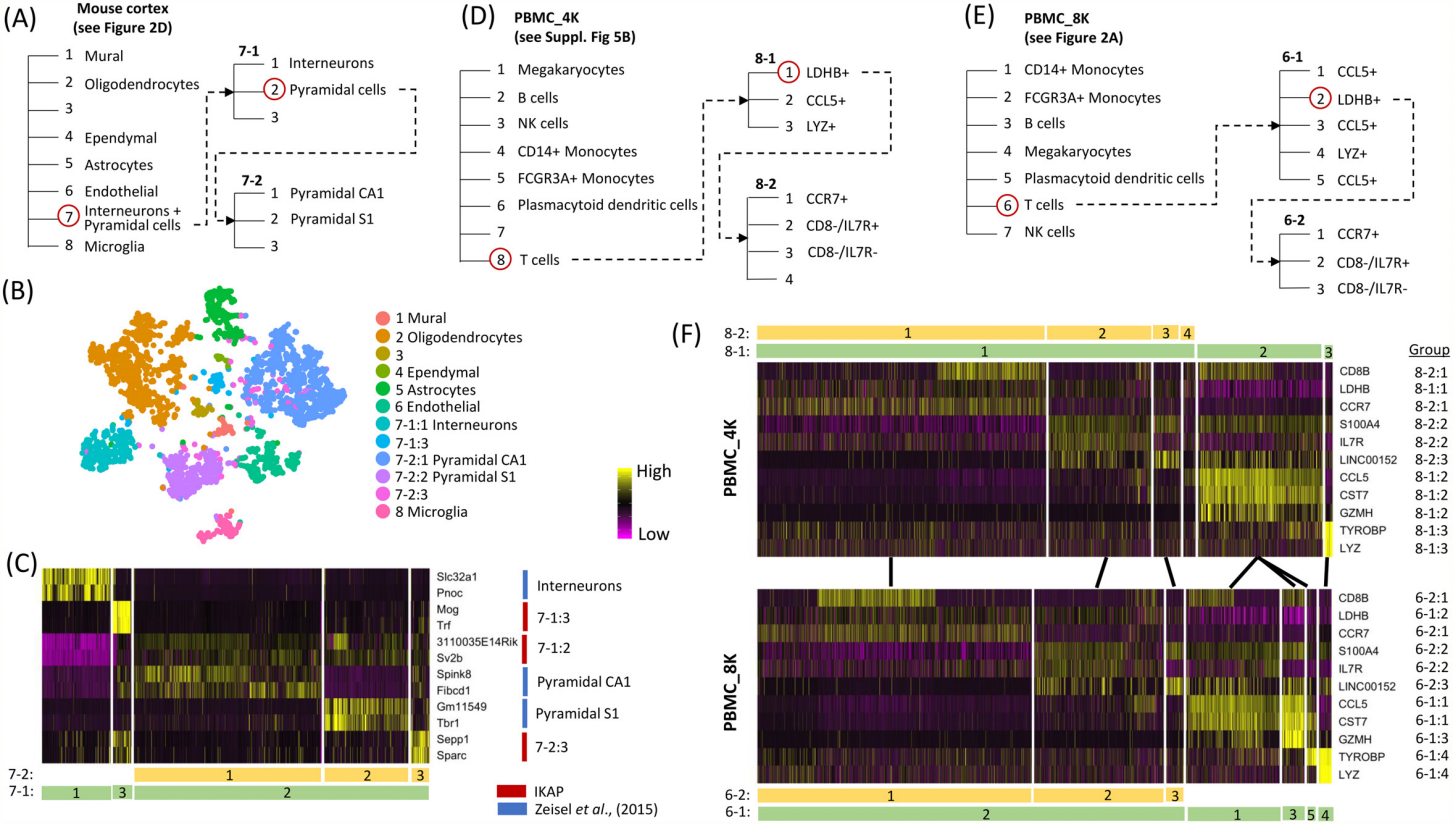
Examples of cell ontology proposed by IKAP. Three cell ontology examples were built by recursively running IKAP on the biggest groups (circled in red) for the mouse cortex dataset (A), PBMC_4K (D), and PBMC_8K (E). Putative cell types were labeled. Unknown types were left as blanks. (B) Shown is the tSNE plot for major groups and subgroups of group 7 presented in the mouse cortex ontology in (A). (C) The heat map shows expression of DE genes identified by IKAP (red) and annotated in Zeisel et al. 2015 [6] (blue) for subgroups of group 7 in (A) (labeled at bottom). (F) Heat maps show expression of selected DE genes that differentiate T-cell subtypes in PBMC_4K (top; subgroups labeled according to the ontology in [D]) and in PBMC_8K (bottom; subgroups labeled according to the ontology in [E]). Subgroups with similar expression profiles are linked by lines between PBMC_4K and PBMC_8K.

Finally, IKAP can be used to recover finer types (or subtypes) by running it recursively within each major cell group. To demonstrate this approach, we expanded 2 layers deeper for all 3 datasets by applying IKAP on their biggest major groups and the biggest resulting subgroups. For the mouse cortex dataset, IKAP successfully recovered interneurons, pyramidal S1, and pyramidal CA1 by subdividing their union group (group 7 in Fig. 2D) (Fig. 3A–C). The 3-layer ontology (Fig. 3A) delineated a more complete view of cell identities in the mouse cortex dataset. It not only reported all previously annotated cell types but also potential high-level cell identities such as the union of interneurons, pyramidal S1, and pyramidal CA1 (Fig. 2E) and the union of 2 pyramidal cell types (Fig. 3C). For PBMC datasets, T cells (the biggest group) were subdivided into 2 layers of subgroups in which subgroups in the same layers were consistent between PBMC_4K and PBMC_8K, suggesting that the ontology built by IKAP could be reproduced in replicates (Fig. 3D–F; Supplementary Figs 10 and 11). Based on identified DE genes, subgroups in the first layer were LDHB^+^, CCL5^+^, and LYZ^+^ T cells. The LDHB^+^ T cells were further divided into CCR7^+^, CD8-/IL7R^+^, and CD8^−^/IL7R^−^ T cells in the second layer. Results shown above were the best sets selected among candidate sets proposed by IKAP. The reported ontology can be modified by manual inspection of different candidate sets. For example, within T cells, a more flattened ontology was achieved by using the candidate set with 7 subgroups (among 4 candidate sets) for PBMC_4K and the set with 10 subgroups (among 5 sets) for PBMC_8K (Supplementary Figs 12–14).

In summary, IKAP can identify major cell groups that produce distinguishing DE genes without the need for specifying clustering parameters, facilitating the automation of the scRNA-seq analysis pipeline. In addition, by recursively applying IKAP within reported cell groups, subtypes can be identified at a finer resolution, delineating cell identities in a multi-layered ontology. As more and more scRNA-seq datasets are generated, it is worth noting that using single-cell transcriptomic data to refine existing cell ontology [7, 8] and to curate reference cell identities [9] is a necessary step forward. Because cell identities are often hierarchical in nature (such as T-cell subtypes within T cells), identifying the hierarchy of cell identities would be informative [10]. For example, rather than classifying PBMCs into subtypes (such as T-cell subtypes and B-cell subtypes) at once, it would be more biologically meaningful to recognize high-level identities such as T cells in the first layer and then subtypes in the next layer. However, not much effort has been made for this task yet. Computationally, it is essentially a task that recursively identifies major groups as parent identities in the upper layer and finer groups within each major group as child identities in the next layer. Conventional methods of estimating *k* by comparing with random data such as the rule of selecting the best *k* in the gap statistic paper [4] and the function sc3_estimate_k implemented in the scRNA-seq clustering package SC3 [11] tend to report many finer groups but miss major groups that represent high-level cell identities. In the present study, we developed IKAP aiming to identify major cell groups in an scRNA-seq dataset and we demonstrate that recursively running IKAP can be used to recover the hierarchy of cell identities for a subset of cells in the mouse cortex and PBMC datasets. We believe that IKAP will greatly assist with refining cell ontology and curation of reference cell identities in the future.

In spite of the advantages mentioned above, several concerns should be noted when using IKAP. First, the performance of IKAP would be affected by upstream data processing such as normalization, covariate removal, feature selection (e.g., selecting variable genes to compute PCs), and dimensional reduction. In addition, human intervention may still be needed to obtain optimal cell type classification (even though “optimal classification” could be subjective). For example, users may need to either apply IKAP within major cell groups to identify finer subgroups as we did to recover interneurons, pyramidal S1, and pyramidal CA1 in the mouse cortex dataset or to manually pick an alternative grouping rather than the best reported by IKAP. Finally, we have shown that IKAP can perform well on the 3 datasets that contain a limited number of discrete cell types with distinct gene expression profiles. However, IKAP may not work as well for more heterogenous datasets such as tumor samples or datasets where expression changes among cells are expected to be gradients such as samples in developmental studies.

In conclusion, IKAP enriches the scRNA-seq analysis toolbox by offering an unbiased solution for picking *k* major cell groups. It not only improves the automation of the scRNA-seq analysis pipeline but also has the ability to refine cell type ontology using single-cell transcriptomes in the future.

## Methods

### IKAP details

IKAP was implemented on top of Seurat (version 2.3.4) [3] in R (version 3.4). When running IKAP by default, it takes only a Seurat object that contains a normalized expression matrix and precomputed covariates that need to be regressed out. The expression matrix will be scaled with covariates regressed out (if provided) using the ScaleData function in Seurat. Then, IKAP finds variable genes using the FindVariableGenes function in Seurat. All Seurat functions are run by default unless a particular setting is specified. Default parameters for IKAP can be easily adjusted by users. Details are discussed in the following.

1. Determine nPC_min_, nPC_max_,and *k*_max_. IKAP avoids specifying a particular number of top principal components (nPC) and *k* by exploring combinations of nPC and *k* (nPC, *k*). nPC_min_, nPC_max_, and *k*_max_ are used to define the search space of (nPC, *k*) such that the combinations (nPC*, k*) that can generate major groups are enclosed (i.e., nPC_min_ ≤ nPC* ≤ nPC_max_ and k* ≤*k*_max_). Setting large nPC_max_ and *k*_max_ increases the search space and the computation time. By following the concept of the elbow method, nPC_min_ is computed as the first principal component (PC) such that a decrease in explained standard deviation relative to the next PCs is <10% for all following PCs. By doing so, the top nPC_min_ PCs should contain informative features to define ≥1 set of major groups. Setting an nPC_max_ > nPC_min_ is for exploring more possible (nPC*, k*) but would not affect the main result much. By default, nPC_max_ is set to nPC_min_ + 20. To set a *k*_max_ ≥k*, we found that setting *r* > 1 in Seurat clustering usually produced many fine groups. So, by default, *k*_max_ is set to the average of the number of resulting groups using the top nPC_min_ PCs and using the top nPC_max_ PCs by setting *r*_ini_ = 1.5. We varied the difference between nPC_max_ and nPC_min_ (nPC_max_ − nPC_min_ = 10, 15, 20, and 25) and *r*_ini_ (=0.9, 1.2, 1.5, and 1.8) to generate 16 test sets for PBMC_4K, PBMC_8K, and the mouse cortex datasets and found that grouping in the reported best sets did not change much (Supplementary Figs 15–17). This shows that our results were not sensitive to the default values of nPC_max_ and *r*_i__ni_.
2. Generate*k*_max_ sets of groups for each nPC. IKAP initializes the set of *k*_ini_ groups by setting *r* = 1.0 using Seurat clustering. If *k*_ini_*< k*_max_, increment *r* by 0.2 until *k*_ini_ ≥*k*_max_. Two nearest groups measured by their centers in the PC space are merged iteratively, generating *k*_ini_ sets of groups but only the first *k*_max_ sets (with *k* = 1 to *k*_max_) are used further.
3. Compute gap statistic. The gap statistic for a set of *k* groups is the difference between the log of sum of within-group pairwise distances over all *k* groups using the actual data and the log of expected sum of within-group pairwise distances over all *k* groups assuming data points (cells) are uniformly distributed in a bounded PC space where boundaries in each dimension are the minimum and the maximum of the actual data in that dimension. Details about gap statistic are described in Tibshirani et al. [4]. Note that the rule of selecting the best *k* proposed in the original gap statistic paper is not used in IKAP.
4. Select the candidate sets. The workflow of selecting candidate sets (PC9K7, PC16K8, and PC18K9) for PBMC_8K is shown in Supplementary Fig. 18. The formal procedure is briefly described as follows. By computing gap increase from a set of *k* − 1 groups to *k* groups (see Step 3 in Fig. 1) for every tested nPC, IKAP generates a gap-increase matrix *M* in which rows correspond to nPC and columns correspond to *k*. Note that each combination of (nPC, *k*) corresponds to a set of cell groups. IKAP first filters out those (nPC, *k*)’s with gap increase ≤ mean + standard deviation. Then, IKAP picks the largest non-zero gap increase for every *k* (every column of *M*), generating a list of gap increases and a list of corresponding (nPC, *k*)’s where *k*’s are different. The list of (nPC, *k*)’s is sorted by corresponding gap increases in descending order. The first (nPC, *k*) (which corresponds to the largest gap increase) is picked as a candidate set. Then, IKAP goes down to the list one by one and adds the (nPC, *k*) to the candidate list if the nPC and *k* are greater than all nPCs and *k*’s already in the candidate list. This requirement is to look for cases where additional cell groups (larger *k*) are identified because of incorporating additional PCs (larger nPC).
5. Compute DE genes. IKAP utilizes the FindAllMarker function in Seurat to compute DE genes for each candidate set. Only upregulated genes are reported. Other parameters are set by default.
6. Build decision trees. The idea of building the decision tree is to evaluate whether a group of cells can be differentiated by considering multiple genes jointly. For each candidate set of cell groups proposed by IKAP, a binary classifier (a decision tree) is built for each group using DE genes from all groups. The decision tree is built by the R package rpart [12] using default parameters.
7. Compute classification error and select the best and alternative candidate sets. The R package rpart builds the decision tree for each group in a candidate set (see "Build decision trees" above) and also reports relative errors (i.e., training errors) at different number of splits (nsplit) along the decision tree. For each group at a given nsplit, the group-level classification error is computed as the product of the relative error and the fraction of cells in that group. The set-level classification error of a candidate set at a given nsplit is defined as the sum of all group-level classification errors. IKAP computes the final classification error for each candidate set as the average of set-level classification errors for nsplit = 5–15. Note that the tree usually did not grow >15 splits in the experiments shown in this study. Finally, among candidate sets, the one with the lowest classification error is marked as the best and the rest are alternatives. In our experience, the number of candidate sets reported by IKAP usually ranges from 1 to 4.

### Performance summary

For each DE gene, the AUROC was computed for classifying its associated group versus others using normalized unique molecular identifier (UMI) count and the function roc.curve in the R package PRROC [13]. We counted the median of numbers of genes with high AUROC (>0.8, 0.85, and 0.9) across all groups. The classification error was computed as described above ("Compute classification error" in "IKAP details"). Average expression log fold change (AvgLFC) was reported by Seurat for each DE gene in each group. For each group, we sorted genes by AvgLFC and only considered DE genes with AUROC > 0.8. Among those, we computed the mean of AvgLFC across the top 10 (or *n* if *n* < 10) DE genes for each group. Running time was measured on a 4.2-GHz Intel Core i7 iMac desktop with 64 GB memory.

### PBMC_4K and PBMC_8K datasets

PBMC_4K and PBMC_8K were downloaded from the 10x Genomics website [5]. They were filtered and normalized using the R package Seurat [3]. We removed cells with <200 genes expressed or the UMI count of mitochondrial genes >5% of the total UMI count. For each dataset, we regressed out the percentage of mitochondrial gene UMI count and the total UMI count from the normalized expression matrix and scaled the matrix using the ScaleData function in Seurat. Finally, we got the expression matrix with 16,746 genes and 4,077 cells for PBMC_4K and the matrix with 18,408 genes and 8,090 cells for PBMC_8K.

### Mouse cortex dataset

The dataset was obtained from Zeisel et al. [6]. We normalized and scaled the expression matrix as we did for PBMC_4K and PBMC_8K, but we did not filter out any cells in order to be consistent with the published work. In total, the expression matrix comprised 19,972 genes and 3,005 cells.

### Cell type recognition

Major cell groups in PBMC datasets (Figs 2A and 3D–E) were annotated on the basis of expression of marker genes and the literature. CD14^+^ monocytes: expression of *LYZ* and *S100A8* [14]. FCGR3A^+^ monocytes: expression of *FCGR3A* and *MS4A7*, a monocyte marker [15]. B cells: expression of *CD79A* [16]. Megakaryocytes: expression of PPBP [17]. Plasmacytoid dendritic cells: expression of *LILRA4* [18]. T cells: expression of *CD3E* [19]. NK cells: expression of *GNLY* but not *CD3E* [20].

## Availability of supporting source code and requirements

Project name: IKAP

Project home page: https://github.com/NHLBI-BCB/IKAP

Operating system(s): Mac OS

Programming language: R

License: MIT license

RRID: IKAP, RRID:SCR_017417

## Availability of supporting data and materials

PBMC_4K and PBMC_8K can be downloaded from the 10x Genomics website [5]. The mouse cortex dataset can be acquired from the GEO database with the accession number GSE60361.

Supporting data are also available via the *GigaScience* repository GigaDB [21].

## Additional files

**Supplementary Figure 1:** Gap statistics increased as more cell groups were identified in PBMC_8K using different numbers of top principal components (nPCs) with large gap increase seen around the number of groups (*k*) = 7, 8, or 9.

**Supplementary Figure 2:** Major cell types in PBMC were not well aligned with the 20 trial sets of cell groups generated for PBMC_8K by varying resolution (*r*) and the number of top principal components (nPC) using Seurat clustering. (A) The tSNE plots for the 20 trial sets. (B) Expression of PBMC type marker genes: *CD3E* for T cells, *CD79A* for B cells, *GNLY* for NK cells, and *LYZ* for monocytes.

**Supplementary Figure 3:** DE gene expression for 6 trial sets selected from the 20 trial sets for PBMC_8K shown in Supplementary Fig. 2. In the heat maps, rows are genes and columns are cells. Groups of cells (separated by vertical white lines) from left to right correspond to groups of corresponding trial sets in Supplementary Fig. 2 in the order of 0, 1, 2, … etc.

**Supplementary Figure 4:** Two alternative sets (PC16K8 and PC18K9) of major cell groups identified for PBMC_8K by IKAP. Shown are tSNE plots of the major groups (left) and heat maps for expression of top DE genes (by expression fold change) (right) for PC16K8 (top) and PC18K9 (bottom). Rows are genes and columns are cells in the heat maps.

**Supplementary Figure 5:** Two candidate sets (PC8K7 and PC20K8) of major groups identified for PBMC_4K by IKAP were aligned with major cell types in PBMC and well differentiated by cell type marker genes. tSNE plots show major groups of PC8K7 (A) and PC20K8 (B). Heat maps show expression of top DE genes (by expression fold change) of each group for PC8K7 (C) and PC20K8 (D). (E) tSNE plots for expression of cell type marker genes: *CD3E* for T cells, *CD79A* for B cells, *GNLY* for NK cells, and *LYZ* for monocytes. (F) Performance summary of the 2 candidate sets proposed by IKAP and 20 trial sets. Running time is shown at the bottom. The dashed blue lines indicate the number of cell groups (top) and the median log_2_ fold change (bottom) of the best set (PC20K8).

**Supplementary Figure 6:** Major cell types in PBMC were not well aligned with the 20 trial sets of cell groups generated for PBMC_4K by varying resolution (*r*) and the number of top principal components (nPC) using Seurat clustering. (A) The tSNE plots for the 20 trial sets. (B) Expression of PBMC cell type marker genes: *CD3E* for T cells, *CD79A* for B cells, *GNLY* for NK cells, and *LYZ* for monocytes.

**Supplementary Figure 7:** Expression of top DE genes for 6 trial sets selected from the 20 trial sets for PBMC_4K shown in Supplementary Fig. 6. In the heat maps, rows are genes and columns are cells. Groups of cells (separated by vertical white lines) from left to right correspond to groups of corresponding trial sets in Supplementary Fig. 6 in the order of 0, 1, 2, … etc.

**Supplementary Figure 8:** Comparison among previously annotated major cell types, major cell groups identified by IKAP, and the 20 trial sets of cell groups for the mouse cortex data. (A) The top tSNE plot shows 9 cell types with original labels in Zeisel et al. 2015 [6] where astrocytes were merged with ependymal and endothelial merged with mural. The modified tSNE plot at bottom recovered ependymal and mural types using group 4 and group 1 identified by IKAP in Fig. 2D. (B) Performance summary of the set proposed by IKAP, the 20 trial sets, and the modified version of major cell types in (A). Running time is shown at the bottom. The dashed blue lines indicate the number of cell groups (top) and the median log_2_ fold change (bottom) of the best set (PC13K8).

**Supplementary Figure 9:** Genes exclusively upregulated in interneurons, pyramidal S1, and pyramidal CA1 in the mouse cortex dataset. Gene expression is indicated by color for each mouse cortex cell in the tSNE plot shown in Fig. 2D. Dark blue indicates high expression whereas grey indicates low expression.

**Supplementary Figure 10:** An example of 2-layer T-cell ontology proposed by IKAP for PBMC_4K. Shown on the left is the ontology with 2 layers (also shown in Fig. 3D). The heat map shows expression of top DE genes (ranked by expression fold change) of each subgroup. Rows are genes and columns are cells.

**Supplementary Figure 11:** An example of 2-layer T-cell ontology proposed by IKAP for PBMC_8K. Shown on the left is the ontology with 2 layers (also shown in Fig. 3E). The heat map shows expression of top DE genes (ranked by expression fold change) of each subgroup. Rows are genes and columns are cells.

**Supplementary Figure 12:** Alternative sets of T-cell subgroups reported by IKAP for PBMC_4K and PBMC_8K. Two PBMC ontologies with T-cell subgroups are shown on the left for PBMC_4K (top) and PBMC_8K (bottom). Expression of DE genes is plotted in heat maps on the right. Rows are genes and columns are cells. Subgroups with similar expression profiles are linked by lines.

**Supplementary Figure 13:** Expression of top DE genes (ranked by expression fold change) for PBMC_4K T cell subgroups shown in Supplementary Fig. 11. Rows are genes and columns are cells.

**Supplementary Figure 14:** Expression of top DE genes (ranked by expression fold change) for PBMC_8K T cell subgroups shown in Supplementary Fig. 11. Rows are genes and columns are cells.

**Supplementary Figure 15:** Major cell groups identified by IKAP were not sensitive to settings of parameters, *r*_ini_ and (nPC_max_ − nPC_min_), for PBMC_4K (see Online Methods). The tSNE plots for 16 sets of major cell groups generated by varying *r*_i__ni_ (=0.9, 1.2, 1.5, and 1.8) and (nPC_max_ − nPC_min_) (=10, 15, 20, and 25) using PBMC_4K. By default *r*_ini_ = 1.5 and (nPC_max_ − nPC_min_) = 20.

**Supplementary Figure 16:** Major cell groups identified by IKAP were not sensitive to settings of parameters, *r_ini_* and (nPC_max_ − nPC_min_), for PBMC_8K (see Online Methods). The tSNE plots for 16 sets of major cell groups generated by varying *r*_ini_ (=0.9, 1.2, 1.5, and 1.8) and (nPC_max_ − nPC_min_) (=10, 15, 20, and 25) using PBMC_8K. By default *r*_ini_ = 1.5 and (nPC_max_ − nPC_min_) = 20.

**Supplementary Figure 17:** Major cell groups identified by IKAP were not sensitive to settings of parameters, *r*_ini_ and (nPC_max_ − nPC_min_), for the mouse cortex dataset (see Online Methods). The tSNE plots for 16 sets of major cell groups generated by varying *r*_ini_ (=0.9, 1.2, 1.5, and 1.8) and (nPC_max_ − nPC_min_) (=10, 15, 20, and 25) using the mouse cortex dataset. By default *r*_ini_ = 1.5 and (nPC_max_ − nPC_min_) = 20.

**Supplementary Figure 18:** The workflow of selecting candidate sets (PC9K7, PC16K8, and PC18K9) for PBMC_8K. Given a gap-increase matrix *M* (see Fig. 1 for how to compute gap increase), the following steps were taken. Step 0: filter entries by gap increases > mean + standard deviation. Step 1: take the maximum gap increase across rows for each column (*k*) and record the corresponding (nPC, *k*). Step 2: sort recorded (nPC, *k*)’s based on their corresponding gap increases. Step 3: add the first (nPC, *k*), which is PC9K7, into the candidate list. Step 4: remove the second (nPC, *k*), which is PC9K6, because its nPC (=9) is not larger than nPC of the candidate (=9) in the candidate list and neither is its *k* not larger than *k* of the candidate (=7) in the candidate list. Step 5: add the third (nPC, *k*) into the candidate list because its nPC (=16) is larger than nPC of the candidate (=9) in the candidate list and so is its *k*. Step 6: add the fourth (nPC, *k*) into the candidate list because its nPC (=20) is larger than all nPCs of the candidates (=9 and 16) in the candidate list and so is its *k*. Finally, PC9K7, PC16K8, and PC18K9 were selected as candidate sets for PBMC_8K.

**Supplementary Table 1**. Median expression of genes significantly* upregulated in the union of interneurons, pyramidal S1, and pyramidal CA1 for the mouse cortex cell types annotated in Zeisel et al. 2015 [6].

giz121_GIGA-D-19-00138_Original_Submission

giz121_GIGA-D-19-00138_Revision_1

giz121_GIGA-D-19-00138_Revision_2

giz121_Response_to_Reviewer_Comments_Original_Submission

giz121_Response_to_Reviewer_Comments_Revision_1

giz121_Reviewer_1_Report_Original_Submission Antoine-Emmanuel Saliba -- 5/31/2019 Reviewed

giz121_Reviewer_2_Report_Original_Submission Philip Lijnzaad -- 6/13/2019 Reviewed

giz121_Reviewer_2_Report_Revision_1 Philip Lijnzaad -- 8/28/2019 Reviewed

giz121_Supplemental_Files

## Abbreviations

AUROC: area under the receiver operating curve; DE: differentially expressed; GEO: Gene Expression Omnibus; NK: natural killer; nPC: the number of top principal components; PBMC: peripheral blood mononuclear cell; PC: principal component; scRNA-seq: single-cell RNA-sequencing; tSNE: T-distributed stochastic neighbor embedding; UMI: unique molecular identifier.

## Competing interests

The authors declare that they have no competing interests.

## Funding

This work was supported by the Intramural Program of the National Heart, Lung, and Blood Institute, National Institutes of Health. Grant number: 1ZICHL006228-02. The funders had no role in study design, data collection and analysis, decision to publish, or preparation of the manuscript.

## Authors’ contributions

M.P. and Y.-C.C. conceived the study. Y.-C.C. developed and implemented the algorithm and drafted the manuscript; A.S. and F.S. helped with implementation; C.U., C.S., K.S., and A.W. helped with cell type annotation. M.P. supervised the research. All authors reviewed and approved the manuscript.
