# Supplementary material for: IKAP—Identifying K mAjor cell Population groups in single-cell RNA-sequencing analysis
Source: Gigascience. 2019 Oct 1;8(10):giz121. doi: 10.1093/gigascience/giz121 (PMC6771546; doi:10.1093/gigascience/giz121)
Supplement: giz121_GIGA-D-19-00138_Revision_1 [file giz121_giga-d-19-00138_revision_1.pdf]

GigaScience

IKAP - Identifying K mAjor cell Population groups in single-cell RNA-seq analysis

--Manuscript Draft--

**Manuscript Number:**	GIGA-D-19-00138R1
**Full Title:**	IKAP - Identifying K mAjor cell Population groups in single-cell RNA-seq analysis
**Article Type:**	Technical Note
**Funding Information:**	NHLBI (1ZICHL006228-02) — Dr. Mehdi Pirooznia
**Abstract:**	Background<br><br>In single-cell RNA-seq analysis, clustering cells into groups and differentiating cell groups by differentially expressed (DE) genes are two separate steps for investigating cell identity. However, the ability to differentiate between cell groups could be affected by clustering. This interdependency often creates a bottleneck in the analysis pipeline, requiring researchers to repeat these two steps multiple times by setting different clustering parameters to identify a set of cell groups that are more differentiated and biologically relevant.<br><br>Findings<br><br>To accelerate this process, we have developed IKAP – an algorithm to identify major cell groups and improve differentiating cell groups by systematically tuning parameters for clustering. We demonstrate that, with default parameters, IKAP successfully identifies major cell types such as T cells, B cells, NK cells, and monocytes in two peripheral blood mononuclear cell (PBMC) datasets and recovers major cell types in a previously published mouse cortex dataset. These major cell groups identified by IKAP present more distinguishing DE genes compared with cell groups generated by different combinations of clustering parameters. We further show that cell subtypes can be identified by recursively applying IKAP within identified major cell types thereby delineating cell identities in a multi-layered ontology.<br><br>Conclusions<br><br>By tuning the clustering parameters to identify major cell groups, IKAP greatly improves the automation of single-cell RNA-seq analysis to produce distinguishing DE genes and refine cell ontology using single-cell RNA-seq data.
**Corresponding Author:**	Mehdi Pirooznia<br><br>UNITED STATES
**Corresponding Author Secondary Information:**	
**Corresponding Author's Institution:**	
**Corresponding Author's Secondary Institution:**	
**First Author:**	Yun-Ching Chen
**First Author Secondary Information:**	
**Order of Authors:**	Yun-Ching Chen
	Abhilash Suresh
	Chingiz Underbayev
	Clare Sun
	Komudi Singh
	Fayaz Seifuddin

Powered by Editorial Manager® and ProduXion Manager® from Aries Systems Corporation

	Adrian Wiestner
	Mehdi Pirooznia
**Order of Authors Secondary Information:**	
**Response to Reviewers:**	Reviewer reports:

Reviewer #1: A major challenge in single-cell RNA-seq data analysis is the gap between the 'clustering' and 'annotation' steps as pointed out by the authors. The first one is usually driven by feature selections and dimension reduction while the latter is done manually based on biological knowledge. The authors build on the existing clustering method called Seurat to propose a rational selection of a clustering that could approximate the 'best' choice in first instance. By optimizing these steps they want to improve the speed and quality of unsupervised clustering. The classification is evaluated by averaging over the expression values of the ten most differentially expressed genes (ranked by average log fold change) and calculating an error statistic. A decision tree is used to determine the candidate with the lowest classification error. To prove the efficacy of their workflow the authors reproduced the results of three publically available datasets from PBMCs and mouse cortex.

When applied to PBMC, the authors failed to identify T-cell subsets showing the limits of the approach (Figure 2). But, the recursive approach proposed in Figure 3 is very interesting and can be applied to find out more subsets.

I believe the approach is interesting for unknown datasets. Soon the multiplication of dataset annotated (notably in the large consortium like the human cell atlas) will allow to develop machine learning approaches to classify the cells automatically. The authors should discuss this point in the discussion.

What are the major claims of the paper?
The authors claim that by choosing a range of principle components and k values, calculating the clustering and ranking them by gap statistic they can identify the biologically most significant clusters and thus improve the overall quality of the unsupervised clustering as well as reducing the runtime.

Are they novel and will they be of interest to others in the community and the wider field?
The Evaluation of data clustering itself is not novel but it has not been applied for single-cell RNA-seq. Guidelines or algorithms for feature selection are needed and this approach completely removes the need to define the clustering parameters manually.

Is the work convincing, and if not, what further evidence would be required to strengthen the conclusions?
This approach is based on the assumption that clustering and differential gene expression analysis are coupled and no knowledge of the cell types is present. When compared to a more biologically driven workflow that discerns clusters based on canonical marker genes it will rather slow down than speed up the analysis.

On a more subjective note, do you feel that the paper will influence thinking in the field?
In my opinion, the authors are pushing the field into the right direction by evaluating the quality of feature selection and unsupervised clustering.

Ability of a researcher to reproduce the work, given the level of detail provided
The authors have provided a clear and structured GitHub repository that should allow full reproducibility and usability of their work and algorithm, respectively.

Response:
We thank the reviewer’s positive comments about IKAP. The reviewer finds the

recursive approach interesting and has suggested that we discuss about how this approach can be used to classify unknown datasets and how it is related to the growing number of annotated datasets such as the human cell atlas (the corresponding reviewer's comments are highlighted above). We have added relevant discussion in the revised manuscript (page 8, paragraph 2) and described our thoughts briefly below.

Because cell identities are hierarchical in nature (e.g. T cell subtypes within T cells), rather than classifying cells into a fixed number of annotated cell types at once, it would be more biologically meaningful to recover the full hierarchy of cell identities. Delineating the hierarchy of cell identities using scRNA-seq data can refine the existing cell ontology [1, 2] and help curate annotations for the reference database such as Human Cell Atlas [3]. Computationally, recovering the hierarchy is essentially a task that recursively identifies major groups as parent identities in the upper layer and finer groups within each major group as child identities in the next layer. In this study, we demonstrate that IKAP recovered the hierarchy of cell identities by constructing a multi-layered ontology for subsets of PBMC datasets and the mouse cortex dataset. Therefore, we believe IKAP can potentially help refine cell ontology and annotate the reference cell identities (such as Human Cell Atlas).

In this version, we have corrected a typo ("pyramidal SS" to "pyramidal S1") and changed the wording for clarity in the revised manuscript. We used differentially expressed (DE) genes for those used to differentiate computationally identified cell groups and reserved "marker genes" for those used to identify manually (or conventionally) annotated cell types. In addition, all major changes in this revised version are highlighted.

Reviewer #2: (NOTE TO READER: I'm using Markdown format below)

Reviewer report

Title: IKAP - Identifying K mAjor cell Population groups in single-cell RNA-seq analysis

Version: Original Submission

Date: June 13, 2019

Review comments to authors:

The authors present a method that elegantly tunes Seurat's clustering parameters to obtain cell clusters that have the best decision tree-classifiability. The method appears to be useful and fast, and by-and-large gives biologically sound results, especially in samples where cells of well-defined type can be expected. I do have some reservations about one of the cases (Mouse brain data by Zeisel et al. 2015) they chose to highlight. The latter may well have prompted the development of a recursive approach which appears to work well and is an interesting extension. I would recommend publication provided the major comments are addressed satisfactorily.

Response:
We thank the reviewer for the positive comments overall. We have revised the manuscript based on the reviewer's comments. All major changes are highlighted. We also provide our point-to-point responses below.

Major comments

* Fig. 2D: It is really unsatisfactory that three clearly separated groups (Interneurons, Pyramidal SS and Pyramidal CA1) are lumped together into one "Group 7". The authors should improve and/or explain this behaviour: is it due to their method (and/or Seurat FindAllMarkers) ending up choosing the wrong differentially expressed genes? If so, why? E.g., most of the Interneurons, Pyramidal SS and Pyramidal CA1 cells are perfectly recognizable in the heatmap (big yellow blocks), were it not for the Atp1a3, Ndrg4 and Stmn3 obscuring things. The latter 3 genes are probably selected

based on their strong under-expression in groups 1 and 2. A number of things could be going on:
1.The decision tree approach yields a poor classifier that badly influences the choice of DE genes. It may not be much work to use a different classifier such as SVM, or even tweaking the parameters given to FindAllMarkers.

2.The authors developed and optimized the method based on the first big 10X Genomics data set, and as a result the method does not generalize well enough. In my experience, the 10X data set is not so easy to classify. Most current cell type classification methods have difficulties with it, see Abdelaal et al. 2019, BioRxiv https://doi.org/10.1101/644435.). It could be remedied by applying their method to another data set, e.g. any of the cancer data sets from the Regev lab (with or without the malignant cells).

3.Confounding factors are at play. It might help to first filter out mitochondrial(ly-related) and ribosomal protein genes and all genes related to the cell cycle before doing anything else.

Response:
The reviewer is concerned that IKAP lumps the 3 cell types: interneurons, pyramidal CA1, and pyramidal S1 (please note that we corrected the typo “pyramidal SS” to “pyramidal S1” in the revision), together due to some unknown issues that need to be fixed because the 3 types are clearly separated groups with distinct marker genes. The reviewer also suspects that the 3 DE genes of the union group of the 3 cell types identified by IKAP were picked because of strong under-expression in group 1 and 2.

We thank the reviewer for providing insights on what could go wrong in the analysis. However, the union group identified by IKAP represents a high-level cell identity shared across interneurons, pyramidal CA1, and pyramidal S1. To support this, we have now added a supplementary figure in the revised manuscript showing that the top 10 genes with high AUROC that classify cells into the union group versus others are highly expressed in the 3 cell types but exhibit low (or no) expression in other types (Supplementary Figure 9). Then, we conducted Wilcoxon rank sum tests showing that all these 10 genes are significantly upregulated in each of the 3 cell types compared with each of the other cell types ($P < 1E-30$ for all tests). This shows that their differential expression was not simply due to strong under-expression in groups 1 and 2 (see Supplementary Table 1). In addition, these 3 cell types are also shown to be close to each other compared to other types in the clustering result in the original paper (see Figure 1C in [4]). These data support that although the 3 cell types expressed distinct marker genes, they also shared certain similarity in gene expression, implicating a potential common cell identity. We have added the analyses mentioned above in the main texts (starting from page 6, line 122).

The reviewer’s comment also raises a key issue that it is not always clear as to what constitutes a cell type. For example, in the PBMC datasets, some people look for T cells whereas others look for T cell subtypes such as CD4+ and CD8+ T cells. All T cells express T cell marker genes but T cell subtypes are also differentiated by subtype marker genes. A similar case is seen for the union group versus the 3 cell types in the mouse cortex dataset. Because cell identities are hierarchical in nature, cell types can be presumably defined at different levels of resolution. To obtain the full picture of cell identities, we believe delineating the hierarchy of cell identities would be more biologically meaningful than classifying cells into a fixed number of reference cell types. Computationally, recovering the hierarchy is essentially a task that recursively identifies major groups as parent identities in the upper layer and finer groups within each major group as child identities in the next layer. We have shown that IKAP recovered the hierarchy of cell identities for subsets of PBMC and the mouse cortex datasets by recursively running it within each major group. We believe IKAP would be helpful for refining cell ontology and curating reference cell identities using scRNA-seq datasets in the future. We have added discussion for the points mentioned above in the main texts (page 8, paragraph 2).

To avoid ambiguity, in the revised manuscript, we use “cell type” for a group of cells that has been manually annotated and defined by a set of marker genes. This clarification has been added in page 5, line 86.

Powered by Editorial Manager® and ProduXion Manager® from Aries Systems Corporation

* The authors should point out (e.g. at the end of the Findings section) that their approach is only suitable for samples where a limited number of definite and distinct cell types can be expected. Their method forces cells into discrete groups, each with a maximally distinct gene expression profile. This will not work in cases with many unknown cells (e.g. tumor samples) or cases where gradients can be expected (time courses, developmental studies).

Response:
As suggested by the reviewer, we have added a paragraph that lists the concerns (including this one) when using IKAP (see page 9, paragraph 2).

* How were the AUROC's calculated? Details in the Performance Summary (page 13) are too few to reproduce.

Response:
We thank the reviewer for pointing out the missing detail. We have now added more details as follow: “For each DE gene, the AUROC (Area Under the ROC curve) was computed for classifying its associated group versus others using normalized UMI count and the function roc.curve in the R package PRROC.” (see page 13, line 268)

Suggestions

* From Suppl. Fig1 it would appear that testing each number of PC's is often superfluous, skipping a few could save time

* I strongly suggest making your package (also) compatible with Seurat 3.x, which is now the default and is likely to be used much more widely

Response:
We thank the reviewer’s suggestions about how to speed up IKAP and the compatibility of the latest Seurat package. We have already implemented a new version of IKAP compatible with Seurat v3. Now it is under the beta testing. We will release it as soon as the testing is completed. We are also compiling ideas to improve IKAP for our next release. This includes speeding up IKAP by skipping calculation of a few PCs.

Minor (editorial) comments:
* throughout the text:
  * a quick read-through by a native speaker should help to fix the missing "the"'s.

Response:
We have fixed the issue as requested by the reviewer.

* the authors should clarify the distinction between differentially expressed (DE) genes (which are inferred, as here, between groups of cells) and marker genes, which are consensus genes whose products are conventionally used to identify cells. It appears that in most occurrences of this term, DE genes are meant, rather than marker genes.

Response:
As suggested by the reviewer, we have replaced “marker genes” with “DE genes” throughout the text when it is meant to be the genes differentially expressed between two computationally identified cell groups and clarified that marker genes are used to define a manually (or conventionally) annotated cell type (see page 5, line 86).

* the terms "feature list" and "choice of features" are in the machine-learning world known as "feature selection" and "feature set". Please change for clarity.

Response:
Changed as suggested by the reviewer.

* provide references for all the R packages used (those for rpart and PRROC are

Powered by Editorial Manager® and ProduXion Manager® from Aries Systems Corporation

missing)

Response:
Both were added.

* Fig. 1, step 4, heatmap: biggest gap increase for nPC=9 visually appears to be for k=5->6, not for k=6->7 as indicated; perhaps adjust the color scale

* Fig. 2C: highlight the fact that chosen 3 parameters (3 leftmost columns) are contrasted with "trial-and-error strategy", e.g. by separating them horizontally a bit. Clarify that it need not be 3 candidates and one 'best'

* Fig. 2: what does the horizontal dashed line signify?

Response:
We thank the reviewer pointing out these details to improve the figures. We have adjusted Figures 1 and 2 and added description to the legend for Figure 2.

(Page numbers below refer to those in the reviewer document)

* page 6:
* "... two user-specified parameters: resolution r ..." This is a unclear, these are Seurat parameters which IKAP doesn't get, but infers itself.

Response:
We have changed the wording.

* " To simulate the coarse-to-fine grouping process ": should be "fine-to-coarse" as merging leads to ever-larger clusters

Response:
Changed.

* "For each set, gap statistic is" : insert "the". Authors should mention that Tibshirani's additional rule for selecting the 'best' k is not used here (why not?)

Response:
We have added “the”, mentioned that Tibshirani's rule of selecting the best k is not used (see page 12, line 231), and briefly explained why the rule is not used in the discussion (see page 9, line 169).

* "Finally, candidate sets grouped": clarify (apparently authors mean sets of k and nPC)

Response:
We have changed the wording to make it clear.

* reference [5]: incomplete reference, provide full download details

Response:
Added url to the download page.

* "marked as the best using decision trees" -> "marked as the best, using decision trees"

Response:
Changed.

* page 7:
* "such that cell lineage" -> "and cell lineage"

Response:
Changed.

	* page 12:<br>* "each group in a candidate set": clarify what a candidate set is.<br><br>Response:<br>We have moved the original step 7 to step 4 in the revised version and made some changes in the Methods to make it clear (see page 12, step 4).<br><br>* page 13:<br>* "IKAP utilizes FindAllMarkers": insert "the"<br><br>Response:<br>Added.<br><br>* "is marked as the best and the rest are alternatives": How many 'alternatives' are there typically?<br><br>Response:<br>Based on the experiments we have tried, the number of candidate sets (including the best and the alternatives) reported by IKAP usually range from 1 to 4. We have added this information in page 13, line 266.<br><br><br>Reference:<br><br>1.Aevermann BD, Novotny M, Bakken T, Miller JA, Diehl AD, Osumi-Sutherland D, et al. Cell type discovery using single-cell transcriptomics: implications for ontological representation. Hum Mol Genet. 2018;27(R1):R40-R7. doi:10.1093/hmg/ddy100<br>2.Bakken T, Cowell L, Aevermann BD, Novotny M, Hodge R, Miller JA, et al. Cell type discovery and representation in the era of high-content single cell phenotyping. BMC Bioinformatics. 2017;18(Suppl 17):559. doi:10.1186/s12859-017-1977-1<br>3.Regev A, Teichmann SA, Lander ES, Amit I, Benoist C, Birney E, et al. The Human Cell Atlas. Elife. 2017;6. doi:10.7554/eLife.27041<br>4.Zeisel A, Munoz-Manchado AB, Codeluppi S, Lonnerberg P, La Manno G, Jureus A, et al. Brain structure. Cell types in the mouse cortex and hippocampus revealed by single-cell RNA-seq. Science. 2015;347(6226):1138-42. doi:10.1126/science.aaa1934
**Additional Information:**	
**Question**	**Response**
Are you submitting this manuscript to a special series or article collection?	No
**Experimental design and statistics**<br><br><br>Full details of the experimental design and statistical methods used should be given in the Methods section, as detailed in our Minimum Standards Reporting Checklist. Information essential to interpreting the data presented should be made available in the figure legends.<br><br><br>Have you included all the information requested in your manuscript?	Yes
**Resources**	Yes

A description of all resources used, including antibodies, cell lines, animals and software tools, with enough information to allow them to be uniquely identified, should be included in the Methods section. Authors are strongly encouraged to cite Research Resource Identifiers (RRIDs) for antibodies, model organisms and tools, where possible.<br><br>Have you included the information requested as detailed in our Minimum Standards Reporting Checklist?	
**Availability of data and materials**<br><br>All datasets and code on which the conclusions of the paper rely must be either included in your submission or deposited in publicly available repositories (where available and ethically appropriate), referencing such data using a unique identifier in the references and in the “Availability of Data and Materials” section of your manuscript.<br><br>Have you have met the above requirement as detailed in our Minimum Standards Reporting Checklist?	Yes

Manuscript

Click here to access/download;Manuscript;manuscript_gigaScience_revised_

Click here to view linked References

IKAP - Identifying K mAjor cell Population groups in single-cell RNA-seq analysis

Yun-Ching Chen[1], Abhilash Suresh[1], Chingiz Underbayev[2], Clare Sun[2], Komudi Singh[1], Fayaz Seifuddin[1], Adrian Wiestner[2], Mehdi Pirooznia[1*]

[1]Bioinformatics and Computational Biology Core, National Heart, Lung, and Blood Institute, National Institutes of Health, Bethesda, United States

[2]Hematology Branch, National Heart, Lung, and Blood Institute, National Institutes of Health, Bethesda, United States

[*]Corresponding author

E-mail: mehdi.pirooznia@nih.gov (MP)

Abstract

Background

In single-cell RNA-seq analysis, clustering cells into groups and differentiating cell groups by differentially expressed (DE) genes are two separate steps for investigating cell identity. However, the ability to differentiate between cell groups could be affected by clustering. This interdependency often creates a bottleneck in the analysis pipeline, requiring researchers to repeat these two steps multiple times by setting different clustering parameters to identify a set of cell groups that are more differentiated and biologically relevant.

Findings

To accelerate this process, we have developed IKAP – an algorithm to identify major cell groups and improve differentiating cell groups by systematically tuning parameters for clustering. We demonstrate that, with default parameters, IKAP successfully identifies major cell types such as T cells, B cells, NK cells, and monocytes in two peripheral blood mononuclear cell (PBMC) datasets and recovers major cell types in a previously published mouse cortex dataset. These major cell groups identified by IKAP present more distinguishing DE genes compared with cell groups generated by different combinations of clustering parameters. We further show that cell subtypes can be identified by recursively applying IKAP within identified major cell types thereby delineating cell identities in a multi-layered ontology.

Conclusions

By tuning the clustering parameters to identify major cell groups, IKAP greatly improves the automation of single-cell RNA-seq analysis to produce distinguishing DE genes and refine cell ontology using single-cell RNA-seq data.

Keywords

Single-cell RNA-seq, clustering, cell ontology, Seurat

Findings

Single-cell RNA-sequencing (scRNA-seq) enables inquiry of cell identity based on single cell transcriptomics. To facilitate cell type characterization and recognition, computational methods have been developed for (*i*) clustering cells with similar transcriptomic profiles into groups and (*ii*) identifying a set of differentially expressed (DE) genes to differentiate those cell groups [1]. These two tasks are frequently treated as independent entities. However, groups identified by clustering greatly determine the DE genes associated with each group. Compared to clustering, computing DE genes is often more resource intensive. We therefore attempted to improve and accelerate biological interpretation of RNA-Seq data by developing an algorithm to effectively identify the *k* major groups that produce distinguishing DE genes.

Despite the existence of well-performing scRNA-seq clustering methods, identifying *k* groups remains a challenge due to parameter specification [2]. Most (if not all) clustering methods require a parameter suggesting *k* and a feature set such as a list of genes or principal components (PCs) for computing cell-to-cell similarity. The proper *k* is generally unknown a priori. Choosing a small *k* may mix more than one cell type in a group whereas choosing a large *k* would result in many subgroups of unclear biological significance. Both can complicate cell type recognition by

producing uninformative DE genes. In addition, feature selection can affect grouping quality which, in turn affects its distinguishing power. Therefore, *k* and feature selection often become a bottleneck in the scRNA-seq analysis pipeline.

To address this issue, we propose an unbiased approach – called IKAP (Identifying K mAjor cell Population groups) – which identifies well-separated *k* major groups poised to produce distinguishing DE genes in a scRNA-seq dataset by systematically exploring the parameter space (Figure 1 and Online Methods). IKAP is implemented on top of Seurat [3] – one of the most widely used scRNA-seq analysis packages – in which clustering requires two parameters that need to be specified by users: resolution *r* that determines *k* (the higher *r*, the larger *k*) and the number of top principal components (nPC) as the feature set. Briefly, for a given nPC, IKAP initializes a set of k_{max} groups by setting a high *r*. To simulate the fine-to-coarse grouping process, two nearest groups are merged iteratively, generating k_{max} sets of groups with k = 1 to k_{max}. For each set, the gap statistic is computed to measure the gap between the grouping with observed data and that with random data [4]. The gap often monotonically increases (at variable amount) as *k* increases from 1 to k_{max} indicating that splitting out each group somewhat contributes to the grouping moving away from randomness (Supplementary Figure 1). We reason that those *k*'s that contribute more (*i.e.* yield large gap increase) might correspond to the set of *k* well-separated major groups. IKAP repeats this procedure for a range of nPCs. Finally, a few candidate sets of cell groups with large gap increases are picked. Among all candidate sets, the one with the lowest classification error is marked as the best, using decision trees built from DE genes. IKAP can be

run default without specifying any parameter as we did for experimentation in this study and can potentially be tailored for scRNA-seq clustering methods other than Seurat.

We tested IKAP on a peripheral blood mononuclear cell (PBMC) dataset of ~8K cells (denoted as PBMC_8K) from a healthy donor [5]. The best set (with *k*=7 and nPC=9; thus, abbreviated as PC9K7) and two alternative sets (PC16K8 and PC18K9 respectively) were reported. The major groups reported in PC9K7 were effectively aligned with known major cell types such as B cells, T cells, and NK cells as evidenced by expression of marker genes (Figure 2A). Note that in this paper, cell types are defined as types of cells that have been manually (or conventionally) annotated and defined by a set of marker genes. Those marker genes of different cell types (such as *CD3E*, *TRAC*, and *IL32* for T cells) were also prioritized to the top of the DE gene list for every group (Figure 2B), facilitating cell type determination. To compare with the trial-and-error strategy, we varied nPC (=5, 10, 15, and 20) and *r* (=0.1, 0.2, 0.4, 0.6, and 1.0) to generate 20 trial sets of groups using Seurat clustering. Most trial sets did not divide cells into major cell types (Supplementary Figure 2) and cell type marker genes were not ranked at the top or were unspecific to particular cell groups, complicating cell type recognition (Supplementary Figure 3). To quantitatively evaluate whether a set of cell groups can produce distinguishing DE genes, we designed three metrics: (*i*) the number of DE genes with high AUROC (Area Under the ROC curve), (*ii*) in-group versus out-of-group expression fold change among high AUROC DE genes, and (*iii*) classification error when classifying cells using decision trees built from multiple DE genes. Compared with the 20 trial sets, we found PC9K7 yielded more DE genes with high AUROC, higher expression fold change, and lower classification error (Figure 2C). Two alternative sets (PC16K8 and PC18K9) also

agreed with major cell types and produced distinguishing DE genes with more rare types or subtypes reported (Figure 2C; Supplementary Figure 4). Finally, IKAP consumed less time (1hr 10m) than computing the 20 trial sets (5hr 13m) (Figure 2C). Although IKAP required an extra step to explore parameter space (19m), much time was saved because of fewer runs (3 candidate sets versus 20 trial sets) of time-consuming DE gene identification. The result shows that IKAP could help biological interpretation by picking appropriate parameters and reporting major cell groups that produce distinguishing DE genes within a reasonable time frame.

To test robustness, we repeated the same analysis for another dataset PBMC_4K (~ 4K cells) from the same donor [5]. This time two candidate sets (PC8K7 and PC20K8) were picked with PC20K8 marked as the best. Compared to the 20 trial sets, IKAP candidate sets were in better agreement with known cell types, cell type marker genes being prioritized at the top, producing more distinguishing DE genes, and consuming shorter running time (Supplementary Figures 5-7).

Next, we applied IKAP on a mouse cortex dataset (~ 3K cells) in which nine major cell types were previously annotated in Zeisel *et al.*, 2015 [6] (Supplementary Figure 8A). IKAP identified one candidate set with 8 major groups (PC13K8) (Figure 2D). Six groups were consistent with the annotated cell types as evidenced by expressing marker genes annotated specific to those cell types (Figure 2E) and high proportion of overlapping cells (Figure 2F). For the 2 remaining groups, one was a subtype of microglia cells (group 3 in Figure 2D) characterized by *Hexb* (AUROC=1.0), *Cst3* (0.98), and *P2ry12* (0.98) and the other was the union of 3 annotated cell types, interneurons, pyramidal S1 and pyramidal CA1 (group 7 in Figure 2D), characterized by many DE genes including

Atp1a3 (0.93), *Ndrg4* (0.94), and *Stmn3* (0.93) (Figure 2E; Supplementary Figure 9). Those genes were significantly upregulated in interneurons, pyramidal S1, and pyramidal CA1 compared with every other cell type annotated in Zeisel *et al*., 2015 [6] (Supplementary Tables 1). The common gene expression profile suggested a high-level cell identity shared across the 3 cell types, which was consistent with the clustering result showing their similarity compared to other cell types in Zeisel *et al*., 2015 (see Figure 1C in [6]). Compared with the 20 trial sets (generated in the same way described above), DE genes in PC13K8 yielded higher expression fold change and lower classification error (Supplementary Figure 8B). Interestingly, the number of DE genes with high AUROC remained high as more subgroups were identified (which was not true for PBMC datasets), suggesting transcriptomic differentiation in the mouse cortex cells was very fine-grained. Overall, IKAP successfully recovered major cell types (rather than many subtypes) that were consistent with previous annotations and produced distinguishing DE genes.

Finally, IKAP can be used to recover finer types (or subtypes) by running it recursively within each major cell group. To demonstrate this approach, we expanded two layers deeper for all 3 datasets by applying IKAP on their biggest major groups and the biggest resulting subgroups. For the mouse cortex dataset, IKAP successfully recovered interneurons, pyramidal S1, and pyramidal CA1 by subdividing their union group (group 7 in Figure 2D) (Figure 3A-C). The 3-layer ontology (Figure 3A) delineated a more complete view of cell identities in the mouse cortex dataset. It not only reported all previously annotated cell types but also potential high-level cell identities such as the union of interneurons, pyramidal S1, and pyramidal CA1 (Figure 2E) and the union of two pyramidal cell types (Figure 3C). For PBMC datasets, T cells (the biggest group) were subdivided

into 2 layers of subgroups in which subgroups in the same layers were consistent between PBMC_4K and PBMC_8K, suggesting the ontology built by IKAP could be reproduced in replicates (Figure 3D-F; Supplementary Figures 10-11). Based on identified DE genes, subgroups in the 1st layer were LDHB+, CCL5+, and LYZ+ T cells. The LDHB+ T cells were further divided into CCR7+, CD8-/IL7R+, and CD8-/IL7R- T cells in the 2nd layer. Results shown above were the best sets selected among candidate sets proposed by IKAP. The reported ontology can be modified by manual inspection of different candidate sets. For example, within T cells, a more flattened ontology was achieved by using the candidate set with 7 subgroups (among 4 candidate sets) for PBMC_4K and the set with 10 subgroups (among 5 sets) for PBMC_8K (Supplementary Figures 12-14).

In summary, IKAP can identify major cell groups that produce distinguishing DE genes without the need for specifying clustering parameters, facilitating the automation of the scRNA-seq analysis pipeline. In addition, by recursively applying IKAP within reported cell groups, subtypes can be identified at a finer resolution, delineating cell identities in a multi-layered ontology. As more and more scRNA-seq datasets are generated, it is worth noting that using single-cell transcriptomic data to refine existing cell ontology [7, 8] and to curate reference cell identities [9] are a necessary step forward. Because cell identities are often hierarchical in nature (such as T cell subtypes within T cells), identifying the hierarchy of cell identities would be informative. For example, rather than classifying PBMCs into subtypes (such as T cell subtypes and B cell subtypes) at once, it would be more biologically meaningful to recognize high-level identities such as T cells in the first layer and then subtypes in the next layer. However, not much effort has

been made for this task yet. Computationally, it is essentially a task that recursively identifies major groups as parent identities in the upper layer and finer groups within each major group as child identities in the next layer. Conventional methods of estimating *k* by comparing with random data such as the rule of selecting the best *k* in the gap statistic paper [4] and the function sc3_estimate_k implemented in the scRNA-seq clustering package SC3 [10] tend to report many finer groups but miss major groups that represent high-level cell identities. In this study, we developed IKAP aiming to identify major cell groups in a scRNA-seq dataset and we demonstrate that recursively running IKAP can be used to recover the hierarchy of cell identities for a subset of cells in the mouse cortex and PBMC datasets. We believe IKAP will greatly assist with refining cell ontology and curation of reference cell identities in the future.

In spite of the advantages mentioned above, several concerns should be noted when using IKAP. First, the performance of IKAP would be affected by upstream data processing such as normalization, covariate removal, feature selection (*e.g.* selecting variable genes to compute PCs), and dimensional reduction. In addition, human intervention may still be needed to obtain optimal cell type classification (even though “optimal classification” could be subjective). For example, users may need to either apply IKAP within major cell groups to identify finer subgroups as we did to recover interneurons, pyramidal S1, and pyramidal CA1 in the mouse cortex dataset or to manually pick an alternative grouping rather than the best reported by IKAP. Finally, we have shown that IKAP can perform well on the 3 datasets which contain a limited number of discrete cell types with distinct gene expression profiles. However, IKAP may not work as well for

more heterogenous datasets such as tumor samples or datasets where expression changes among cells are expected to be gradients such as samples in developmental studies.

In conclusion, IKAP enriches the scRNA-seq analysis toolbox by offering an unbiased solution for picking *k* major cell groups. It not only improves the automation of the scRNA-seq analysis pipeline but also has the ability to refine cell type ontology using single-cell transcriptomes in the future.

Methods

IKAP details. IKAP was implemented on top of Seurat (version 2.3.4) [3] in R (version 3.4). When running IKAP by default, it takes only a Seurat object which contains a normalized expression matrix and pre-computed covariates that need to be regressed out. The expression matrix will be scaled with covariates regressed out (if provided) using the ScaleData function in Seurat. Then, IKAP finds variable genes using the FindVariableGenes function in Seurat. All Seurat functions are run by default unless particular setting is specified. Default parameters for IKAP can be easily adjusted by users. Details are discussed in the following.

1. *Determine* nPC_{min}*,* nPC_{max}*, and* k_{max}. IKAP avoids specifying a particular number of top principal components (nPC) and *k* by exploring combinations of nPC and *k* (nPC, *k*). nPC_{min}, nPC_{max}, and k_{max} are used to define the search space of (nPC, *k*) such that the combinations (nPC^*, k^*) that can generate major groups are enclosed (*i.e.* $nPC_{min} \leq nPC^* \leq nPC_{max}$ and $k^* \leq k_{max}$). Setting large nPC_{max} and k_{max} increases the search space and the computation time. By following the concept of elbow method, nPC_{min} is computed as the first principal

component (PC) such that a decrease in explained standard deviation relative to the next PCs is less than 10% for all following PCs. By doing so, the top nPC_{min} PCs should contain informative features to define at least one set of major groups. Setting a $nPC_{max} > nPC_{min}$ is for exploring more possible (nPC^*, k^*) but would not affect the main result much. By default, nPC_{max} is set to $nPC_{min} + 20$. To set a $k_{max} \geq k^*$, we found setting $r > 1$ in Seurat clustering usually produced many fine groups. So, by default, k_{max} is set to the average of the number of resulting groups using the top nPC_{min} PCs and that using the top nPC_{max} PCs by setting $r_{ini} = 1.5$. We varied the difference between nPC_{max} and nPC_{min} ($nPC_{max} - nPC_{min}$ =10, 15, 20, and 25) and r_{ini} (=0.9, 1.2, 1.5, and 1.8) to generate 16 test sets for PBMC_4K, PBMC_8K, and the mouse cortex datasets and found grouping in the reported best sets did not change much (Supplementary Figures 15-17). This shows that our results were not sensitive to the default values of nPC_{max} and r_{ini}.

2. *Generate k_{max} sets of groups for each nPC*. IKAP initializes the set of k_{ini} groups by setting r=1.0 using Seurat clustering. If $k_{ini} < k_{max}$, increment r by 0.2 until $k_{ini} >= k_{max}$. Two nearest groups measured by their centers in the PC space are merged iteratively, generating k_{ini} sets of groups but only the first k_{max} sets (with k=1 to k_{max}) are used further.
3. *Compute gap statistic*. The gap statistic for a set of k groups is the difference between the log of sum of within-group pairwise distances over all k groups using the actual data and the log of expected sum of within-group pairwise distances over all k groups assuming data points (cells) are uniformly distributed in a bounded PC space where boundaries in each dimension are the minimum and the maximum of the actual data in that dimension.

Details about gap statistic are described in [4]. Note that the rule of selecting the best *k* proposed in the original gap statistic paper is not used in IKAP.

4. *Select the candidate sets*. The workflow of selecting candidate sets (PC9K7, PC16K8, and PC18K9) for PBMC_8K is shown in Supplementary Figure 18. The formal procedure is briefly described as follows. By computing gap increase from a set of *k*-1 groups to *k* groups (see Step 3 in Figure 1) for every tested nPC, IKAP generates a gap-increase matrix *M* in which rows correspond to nPC and columns correspond to *k*. Note that each combination of (nPC, *k*) corresponds to a set of cell groups. IKAP first filters out those (nPC, *k*)'s with gap increase ≤ mean + standard deviation. Then, IKAP picks the largest non-zero gap increase for every *k* (every column of *M*), generating a list of gap increases and a list of corresponding (nPC, *k*)'s where *k*'s are different. The list of (nPC, *k*)'s is sorted by corresponding gap increases in descending order. The first (nPC, *k*) (which corresponds to the largest gap increase) is picked as a candidate set. Then, IKAP goes down to the list one by one and adds the (nPC, *k*) to the candidate list if the nPC and *k* are greater than all nPCs and *k*'s already in the candidate list. This requirement is to look for cases where additional cell groups (larger *k*) are identified because of incorporating additional PCs (larger nPC).
5. *Compute DE genes*. IKAP utilizes the FindAllMarker function in Seurat to compute DE genes for each candidate set. Only upregulated genes are reported. Other parameters are set by default.
6. *Build decision trees*. The idea of building the decision tree is to evaluate if a group of cells can be differentiated by considering multiple genes jointly. For each candidate set of cell groups proposed by IKAP, a binary classifier (a decision tree) is built for each group using

DE genes from all groups. The decision tree is built by the R package rpart [11] using default parameters.

7. *Compute classification error and select the best and alternative candidate sets*. The R package rpart builds the decision tree for each group in a candidate set (see *Build decision trees* above) and also reports relative errors (*i.e.* training errors) at different number of splits (*nsplit*) along the decision tree. For each group at a given *nsplit*, the group-level classification error is computed as the product of the relative error and the fraction of cells in that group. The set-level classification error of a candidate set at a given *nsplit* is defined as the sum of all group-level classification errors. IKAP computes the final classification error for each candidate set as the average of set-level classification errors for *nsplit* = 5 to 15. Note that the tree usually did not grow more than 15 splits in the experiments shown in this study. Finally, among candidate sets, the one with the lowest classification error is marked as the best and the rest are alternatives. In our experience, the number of candidate sets reported by IKAP usually ranges from 1 to 4.

Performance summary. For each DE gene, the AUROC (Area Under the ROC curve) was computed for classifying its associated group versus others using normalized UMI count and the function *roc.curve* in the R package PRROC [12]. We counted the median of numbers of genes with high AUROC (> 0.8, 0.85, and 0.9) across all groups. The classification error was computed as described above (*Compute classification error* in **IKAP details**). Average expression log fold change (AvgLFC) was reported by Seurat for each DE gene in each group. For each group, we sorted genes by AvgLFC and only considered DE genes with AUROC > 0.8. Among those, we computed the mean

of AvgLFC across top 10 (or n if n < 10) DE genes for each group. Running time was measured on 4.2 GHz Intel Core i7 iMac desktop with 64 GB memory.

PBMC_4K and PBMC_8K datasets. PBMC_4K and PBMC_8K were downloaded from the 10x Genomics website [5]. They were filtered and normalized using the R package Seurat [3]. We removed cells with less than 200 genes expressed or the unique molecular identifier (UMI) count of mitochondrial genes > 5% of the total UMI count. For each dataset, we regressed out the percentage of mitochondrial gene UMI count and the total UMI count from the normalized expression matrix and scaled the matrix using ScaleData function in Seurat. Finally, we got the expression matrix with 16,746 genes and 4,077 cells for PBMC_4K and the matrix with 18,408 genes and 8090 cells for PBMC_8K.

Mouse cortex dataset. The dataset was obtained from [6]. We normalized and scaled the expression matrix as we did for PBMC_4K and PBMC_8K but we did not filter out any cells in order to be consistent with the published work. In total, the expression matrix comprised 19,972 genes and 3,005 cells.

Cell type recognition. Major cell groups in PBMC datasets (Figure 2A and Figure 3D-E) were annotated based on expression of marker genes and literature. CD14+ monocytes: expression of *LYZ* and *S100A8* [13]. FCGR3A+ monocytes: expression of *FCGR3A* and *MS4A7*, a monocyte marker [14]. B cells: expression of *CD79A* [15]. Megakaryocytes: expression of PPBP [16]. Plasmacytoid dendritic cells: expression of *LILRA4* [17]. T cells: expression of *CD3E* [18]. NK cells: expression of *GNLY* but not *CD3E* [19].

Availability of supporting source code and requirements

Project name: IKAP

Project home page: https://github.com/NHLBI-BCB/IKAP

Operating system(s): Mac OS

Programming language: R

License: MIT license

Availability of supporting data

PBMC_4K and PBMC_8K can be downloaded from 10x Genomics website: https://www.10xgenomics.com/resources/datasets/. The mouse cortex dataset can be acquired from the accession numbers provided in the original publications.

Declarations

List of abbreviations

AUROC: Area under the ROC curve; nPC: the number of top principal components; PBMC: peripheral blood mononuclear cell; PC: principal component; scRNA: single-cell RNA-sequencing

Competing interests

The author(s) declare that they have no competing interests.

Funding

This work was supported by the Intramural Program of the National Heart, Lung, and Blood Institute, National Institutes of Health. Grant number: 1ZICHL006228-02. The funders had no role in study design, data collection and analysis, decision to publish, or preparation of the manuscript.

Authors' contributions

M.P. and Y.C. conceived the study. Y.C. developed and implemented the algorithm and drafted the manuscript; A.S. and F.S. helped with implementation; C.U., C.S., K.S., and A.W. helped with cell type annotation. M.P. supervised the research. All authors reviewed and approved the manuscript.

References

1. Andrews TS, Hemberg M. Identifying cell populations with scRNASeq. Mol Aspects Med. 2018;59:114-22. doi:10.1016/j.mam.2017.07.002

2. Kiselev VY, Andrews TS, Hemberg M. Challenges in unsupervised clustering of single-cell RNA-seq data. Nat Rev Genet. 2019. doi:10.1038/s41576-018-0088-9

3. Butler A, Hoffman P, Smibert P, Papalexi E, Satija R. Integrating single-cell transcriptomic data across different conditions, technologies, and species. Nat Biotechnol. 2018;36(5):411-20. doi:10.1038/nbt.4096

4. Tibshirani R, Walther G, Hastie T. Estimating the number of clusters in a data set via the gap statistic. J Roy Stat Soc B. 2001;63:411-23. doi:Doi 10.1111/1467-9868.00293

5. Genomics x. Support: single cell gene expression datasets 2017 [Available from: https://www.10xgenomics.com/resources/datasets/.

6. Zeisel A, Munoz-Manchado AB, Codeluppi S, Lonnerberg P, La Manno G, Jureus A, et al. Brain structure. Cell types in the mouse cortex and hippocampus revealed by single-cell RNA-seq. Science. 2015;347(6226):1138-42. doi:10.1126/science.aaa1934

7. Aevermann BD, Novotny M, Bakken T, Miller JA, Diehl AD, Osumi-Sutherland D, et al. Cell type discovery using single-cell transcriptomics: implications for ontological representation. Hum Mol Genet. 2018;27(R1):R40-R7. doi:10.1093/hmg/ddy100

8. Bakken T, Cowell L, Aevermann BD, Novotny M, Hodge R, Miller JA, et al. Cell type discovery and representation in the era of high-content single cell phenotyping. BMC Bioinformatics. 2017;18(Suppl 17):559. doi:10.1186/s12859-017-1977-1

9. Regev A, Teichmann SA, Lander ES, Amit I, Benoist C, Birney E, et al. The Human Cell Atlas. Elife. 2017;6. doi:10.7554/eLife.27041

10. Kiselev VY, Kirschner K, Schaub MT, Andrews T, Yiu A, Chandra T, et al. SC3: consensus clustering of single-cell RNA-seq data. Nat Methods. 2017;14(5):483-6. doi:10.1038/nmeth.4236

11. Therneau T, Atkinson E. An Introduction to Recursive Partitioning Using the rpart Routine. 1997 [Available from: http://www.mayo.edu/hsr/techrpt/61.pdf.

12. Grau J, Grosse I, Keilwagen J. PRROC: computing and visualizing precision-recall and receiver operating characteristic curves in R. Bioinformatics. 2015;31(15):2595-7. doi:10.1093/bioinformatics/btv153

13. Zawada AM, Rogacev KS, Rotter B, Winter P, Marell RR, Fliser D, et al. SuperSAGE evidence for CD14++CD16+ monocytes as a third monocyte subset. Blood. 2011;118(12):e50-61. doi:10.1182/blood-2011-01-326827

14. Gingras MC, Lapillonne H, Margolin JF. CFFM4: a new member of the CD20/FcepsilonRIbeta family. Immunogenetics. 2001;53(6):468-76. doi:10.1007/s002510100345

15. Chu PG, Arber DA. CD79: a review. Appl Immunohistochem Mol Morphol. 2001;9(2):97-106.

16. Zhang C, Gadue P, Scott E, Atchison M, Poncz M. Activation of the megakaryocyte-specific gene platelet basic protein (PBP) by the Ets family factor PU.1. J Biol Chem. 1997;272(42):26236-46.

17. Cho M, Ishida K, Chen J, Ohkawa J, Chen W, Namiki S, et al. SAGE library screening reveals ILT7 as a specific plasmacytoid dendritic cell marker that regulates type I IFN production. Int Immunol. 2008;20(1):155-64. doi:10.1093/intimm/dxm127
18. Chetty R, Gatter K. CD3: structure, function, and role of immunostaining in clinical practice. J Pathol. 1994;173(4):303-7. doi:10.1002/path.1711730404
19. Pena SV, Krensky AM. Granulysin, a new human cytolytic granule-associated protein with possible involvement in cell-mediated cytotoxicity. Semin Immunol. 1997;9(2):117-25. doi:10.1006/smim.1997.0061

Figure legends

Figure 1. IKAP workflow. See Online Methods for details.

Figure 2. Major cell groups identified for PBMC_8K (A, B, and C) and the mouse cortex dataset (D, E, and F). (A) Shown are tSNE plots for the 7 major groups identified by IKAP with cell types labeled (*top*) and expression of known marker genes (*bottom*): *CD3E* for T cells, *CD79A* for B cells, *GNLY* for NK cells, and *LYZ* for monocytes. (B) The heatmap for expression of the top 5 DE genes (by expression fold change) from each group in (A). Rows are genes and columns are cells. (C) Performance summary of 3 candidate sets proposed by IKAP (*left*) and the 20 trial sets (*right*). Note that the number of candidate sets can vary for different datasets. Running time is shown at the bottom. The dashed blue lines indicate the number of cell groups (*top*) and the median log2 fold change (*bottom*) of the best set (PC9K7). (D) The tSNE plot for 8 major groups identified by IKAP in the mouse cortex dataset consistent with previously annotated cell types. (E) The heatmap for expression of marker genes annotated for major cell types in Zeisel *et al*., 2015 [6] (*blue*) and DE genes identified by IKAP for groups 3 and 7 in (D) (*red*). (F) The heatmap indicates the proportion of overlapping cells between IKAP-identified major groups and major cell types annotated in Zeisel *et al*., 2015 [6].

Figure 3. Examples of cell ontology proposed by IKAP. Three cell ontology examples were built by recursively running IKAP on the biggest groups (circled in red) for the mouse cortex dataset (A), PBMC_4K (D), and PBMC_8K (E). Putative cell types were labeled. Unknown types were left as blanks. (B) Shown is the tSNE plot for major groups and subgroups of group 7 presented in the

mouse cortex ontology in (A). (C) The heatmap shows expression of DE genes identified by IKAP (*red*) and annotated in Zeisel *et al.*, 2015 [6] (*blue*) for subgroups of group 7 in (A) (labeled at bottom). (F) Heatmaps show expression of selected DE genes that differentiate T cell subtypes in PBMC_4K (*top*; subgroups labeled according to the ontology in (D)) and in PBMC_8K (*bottom*; subgroups labeled according to the ontology in (E)). Subgroups with similar expression profiles are linked by lines between PBMC_4K and PBMC_8K.

Supplementary figure legends

Supplementary Figure 1. Gap statistics increased as more cell groups were identified in PBMC_8K using different numbers of top principal components (nPCs) with large gap increase seen around the number of groups (*k*) = 7, 8, or 9.

Supplementary Figure 2. Major cell types in PBMC were not well aligned with the 20 trial sets of cell groups generated for PBMC_8K by varying resolution (*r*) and the number of top principal components (nPC) using Seurat clustering. (A) The tSNE plots for the 20 trial sets. (B) Expression of PBMC type marker genes: *CD3E* for T cells, *CD79A* for B cells, *GNLY* for NK cells and *LYZ* for monocytes.

Supplementary Figure 3. DE gene expression for 6 trial sets selected from the 20 trial sets for PBMC_8K shown in Supplementary Figure 2. In the heatmaps, rows are genes and columns are cells. Groups of cells (separated by vertical white lines) from left to right correspond to groups of corresponding trial sets in Supplementary Figure 2 in the order of 0, 1, 2, … etc.

Supplementary Figure 4. Two alternative sets (PC16K8 and PC18K9) of major cell groups identified for PBMC_8K by IKAP. Shown are tSNE plots of the major groups (*left*) and heatmaps for expression of top DE genes (by expression fold change) (*right*) for PC16K8 (*top*) and PC18K9 (*bottom*). Rows are genes and columns are cells in the heatmaps.

Supplementary Figure 5. Two candidate sets (PC8K7 and PC20K8) of major groups identified for PBMC_4K by IKAP were aligned with major cell types in PBMC and well differentiated by cell type marker genes. tSNE plots show major groups of PC8K7 (A) and PC20K8 (B). Heatmaps show expression of top DE genes (by expression fold change) of each group for PC8K7 (C) and PC20K8 (D). (E) tSNE plots for expression of cell type marker genes: *CD3E* for T cells, *CD79A* for B cells, *GNLY* for NK cells and *LYZ* for monocytes. (F) Performance summary of the 2 candidate sets proposed by IKAP and 20 trial sets. Running time is shown at the bottom. The dashed blue lines indicate the number of cell groups (*top*) and the median log2 fold change (*bottom*) of the best set (PC20K8)

Supplementary Figure 6. Major cell types in PBMC were not well aligned with the 20 trial sets of cell groups generated for PBMC_4K by varying resolution (*r*) and the number of top principal components (nPC) using Seurat clustering. (A) The tSNE plots for the 20 trial sets. (B) Expression of PBMC cell type marker genes: *CD3E* for T cells, *CD79A* for B cells, *GNLY* for NK cells and *LYZ* for monocytes.

Supplementary Figure 7. Expression of top DE genes for 6 trial sets selected from the 20 trial sets for PBMC_4K shown in Supplementary Figure 6. In the heatmaps, rows are genes and columns are cells. Groups of cells (separated by vertical white lines) from left to right correspond to groups of corresponding trial sets in Supplementary Figure 6 in the order of 0, 1, 2, … etc.

Supplementary Figure 8. Comparison among previously annotated major cell types, major cell groups identified by IKAP, and the 20 trial sets of cell groups for the mouse cortex data. (A) The top tSNE plot shows 9 cell types with original labels in Zeisel *et al.*, 2015 [6] where astrocytes were merged with ependymal and endothelial merged with mural. The modified tSNE plot at bottom recovered ependymal and mural types using group 4 and group 1 identified by IKAP in Figure 2D. (B) Performance summary of the set proposed by IKAP, the 20 trial sets, and the modified version of major cell types in (A). Running time is shown at the bottom. The dashed blue lines indicate the number of cell groups (*top*) and the median log2 fold change (*bottom*) of the best set (PC13K8)

Supplementary Figure 9. Genes exclusively upregulated in interneurons, pyramidal S1, and pyramidal CA1 in the mouse cortex dataset. Gene expression is indicated by color for each mouse cortex cell in the tSNE plot shown in Figure 2D. Dark blue indicates high expression whereas grey indicates low expression.

Supplementary Figure 10. An example of 2-layer T cell ontology proposed by IKAP for PBMC_4K. Shown on the left is the ontology with two layers (also shown in Figure 3D). The heatmap shows expression of top DE genes (ranked by expression fold change) of each subgroup. Rows are genes and columns are cells.

Supplementary Figure 11. An example of 2-layer T cell ontology proposed by IKAP for PBMC_8K. Shown on the left is the ontology with two layers (also shown in Figure 3E). The heatmap shows

expression of top DE genes (ranked by expression fold change) of each subgroup. Rows are genes and columns are cells.

Supplementary Figure 12. Alternative sets of T cell subgroups reported by IKAP for PBMC_4K and PBMC_8K. Two PBMC ontologies with T cell subgroups are shown on the left for PBMC_4K (*top*) and PBMC_8K (*bottom*). Expression of DE genes is plotted in heatmaps on the right. Rows are genes and columns are cells. Subgroups with similar expression profiles are linked by lines.

Supplementary Figure 13. Expression of top DE genes (ranked by expression fold change) for PBMC_4K T cell subgroups shown in Supplementary Figure 11. Rows are genes and columns are cells.

Supplementary Figure 14. Expression of top DE genes (ranked by expression fold change) for PBMC_8K T cell subgroups shown in Supplementary Figure 11. Rows are genes and columns are cells.

Supplementary Figure 15. Major cell groups identified by IKAP were not sensitive to settings of parameters, r_{ini} and (nPC_{max}-nPC_{min}), for PBMC_4K (see Online Methods). The tSNE plots for 16 sets of major cell groups generated by varying r_{ini} (=0.9, 1.2, 1.5, and 1.8) and (nPC_{max}-nPC_{min}) (=10, 15, 20, and 25) using PBMC_4K. By default r_{ini}=1.5 and (nPC_{max}-nPC_{min})=20.

Supplementary Figure 16. Major cell groups identified by IKAP were not sensitive to settings of parameters, r_{ini} and (nPC_{max}-nPC_{min}), for PBMC_8K (see Online Methods). The tSNE plots for 16 sets of major cell groups generated by varying r_{ini} (=0.9, 1.2, 1.5, and 1.8) and (nPC_{max}-nPC_{min}) (=10, 15, 20, and 25) using PBMC_8K. By default r_{ini}=1.5 and (nPC_{max}-nPC_{min})=20.

Supplementary Figure 17. Major cell groups identified by IKAP were not sensitive to settings of parameters, r_{ini} and (nPC_{max}-nPC_{min}), for the mouse cortex dataset (see Online Methods). The tSNE plots for 16 sets of major cell groups generated by varying r_{ini} (=0.9, 1.2, 1.5, and 1.8) and (nPC_{max}-nPC_{min}) (=10, 15, 20, and 25) using the mouse cortex dataset. By default r_{ini}=1.5 and (nPC_{max}-nPC_{min})=20.

Supplementary Figure 18. The workflow of selecting candidate sets (PC9K7, PC16K8, and PC18K9) for PBMC_8K. Given a gap-increase matrix *M* (see Figure 1 for how to compute gap increase), the following steps were taken. Step 0: filter entries by gap increases > mean + standard deviation. Step 1: take the max gap increase across rows for each column (*k*) and record the corresponding (nPC, *k*). Step 2: sort recorded (nPC, *k*)'s based on their corresponding gap increases. Step 3: add the first (nPC, *k*), which is PC9K7, into the candidate list. Step 4: remove the second (nPC, *k*), which is PC9K6, because its nPC (=9) is not larger than nPC of the candidate (=9) in the candidate list and neither is its *k* not larger than *k* of the candidate (=7) in the candidate list. Step 5: add the third (nPC, *k*) into the candidate list because its nPC (=16) is larger than nPC of the candidate (=9) in the candidate list and so is its *k*. Step 6: add the fourth (nPC, *k*) into the candidate list because its nPC (=20) is larger than all nPCs of the candidates (=9 and 16) in the

candidate list and so is its *k*. Finally, PC9K7, PC16K8, and PC18K9 were selected as candidate sets for PBMC_8K.

Figure 1

Click here to access/download;Figure;Figure 1.pptx

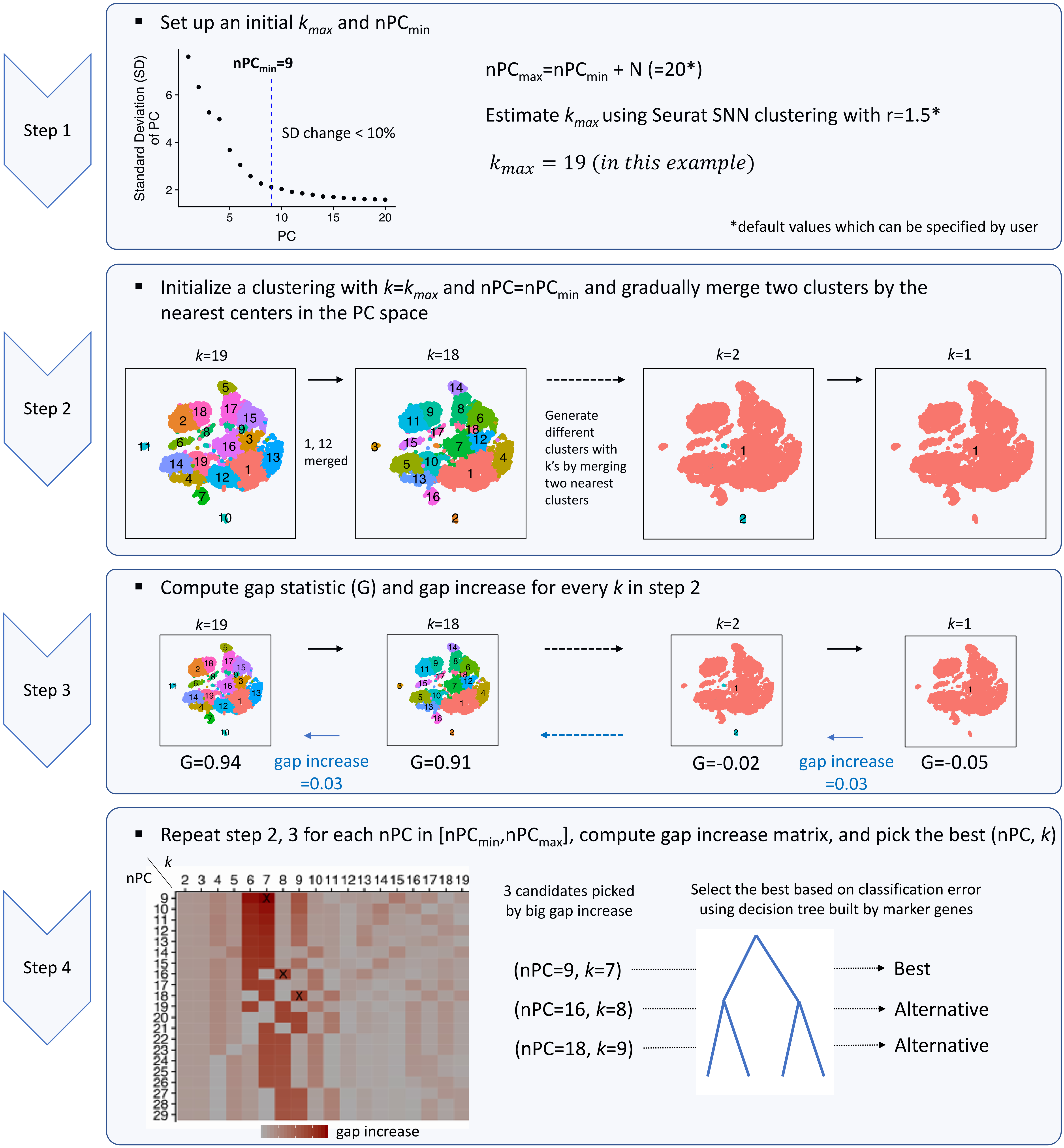

Figure 2

Click here to access/download;Figure;Figure 2.pptx

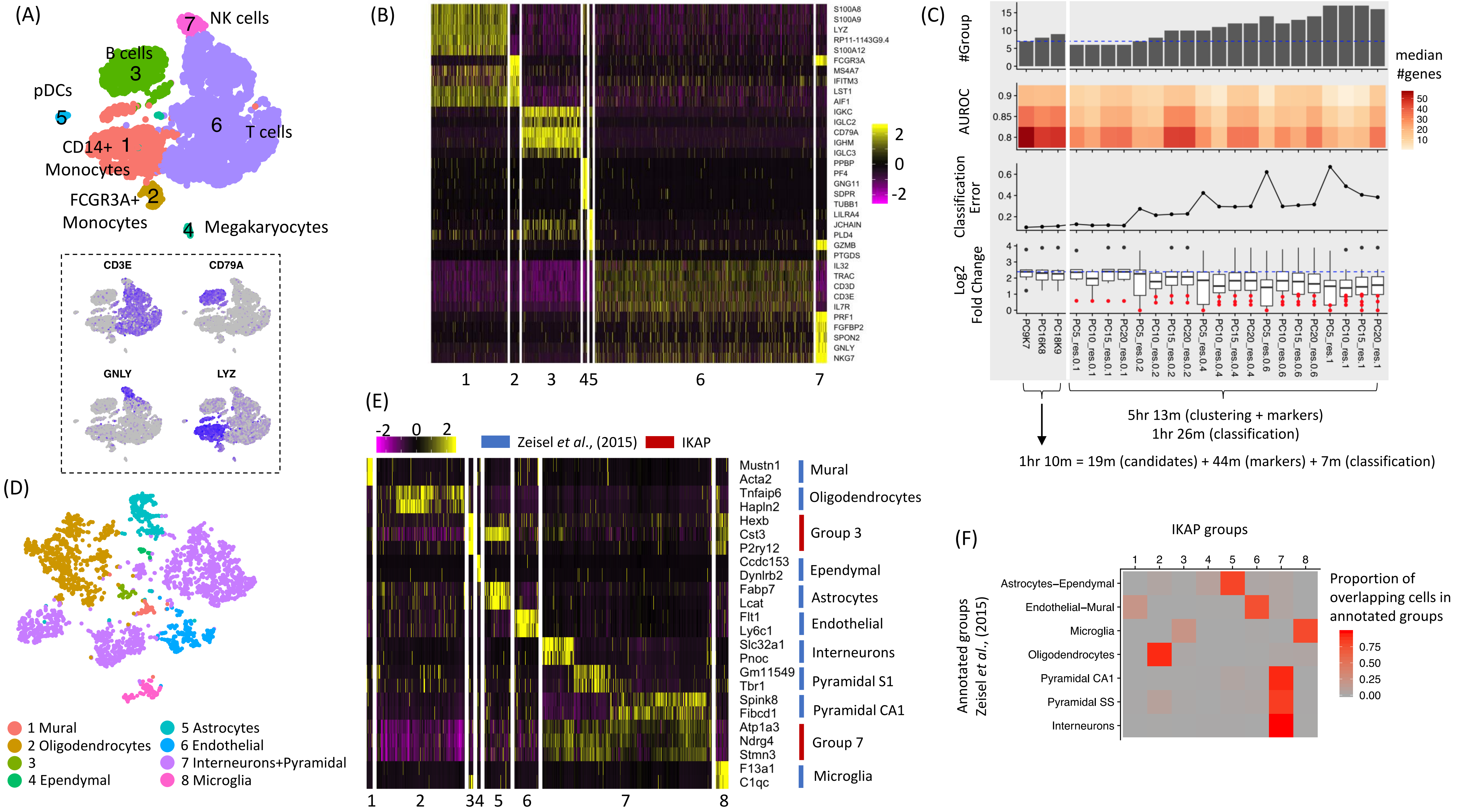

Figure 3

Click here to access/download;Figure;Figure 3.pptx

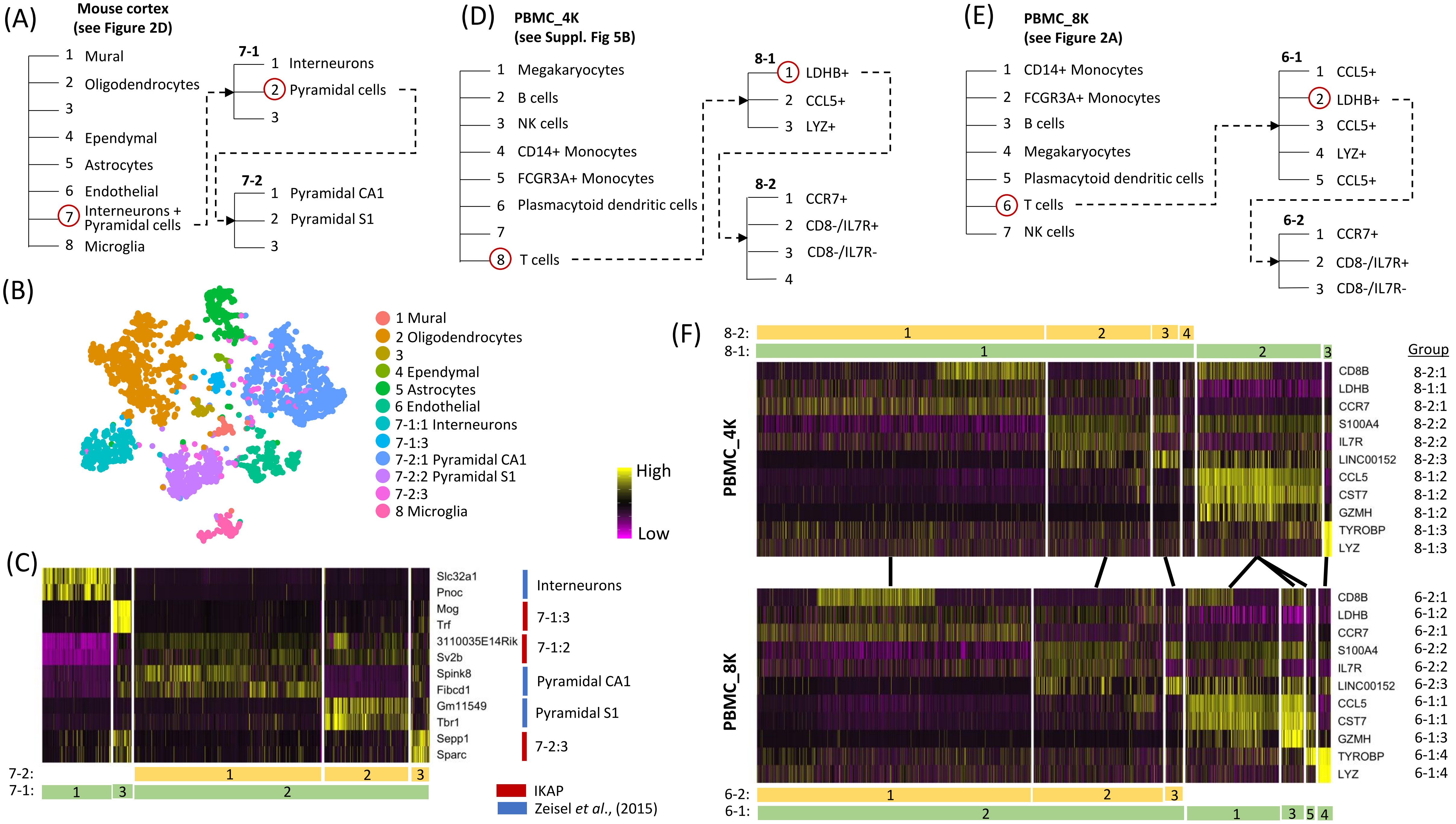

Supplementary Table 1

Click here to access/download
Supplementary Material
Supplementary Table 1.docx

Supplementary Figure 1

Click here to access/download
Supplementary Material
Supplementary Figure 1.pdf

Supplementary Figure 2

Click here to access/download
Supplementary Material
Supplementary Figure 2.pdf

Supplementary Figure 3

Click here to access/download
Supplementary Material
Supplementary Figure 3.pdf

Supplementary Figure 4

Click here to access/download
Supplementary Material
Supplementary Figure 4.pdf

Supplementary Figure 6

Click here to access/download
Supplementary Material
Supplementary Figure 6.pdf

Supplementary Figure 7

Click here to access/download
Supplementary Material
Supplementary Figure 7.pdf

Supplementary Figure 5

Click here to access/download

Supplementary Material

Supplementary Figure5.pdf

Supplementary Figure 8

Click here to access/download
Supplementary Material
Supplementary Figure8.pdf

Supplementary Figure 9

Click here to access/download
Supplementary Material
Supplementary Figure9.pdf

Supplementary Figure 10

Click here to access/download
Supplementary Material
Supplementary Figure10.pdf

Supplementary Figure 11

Click here to access/download
Supplementary Material
Supplementary Figure11.pdf

Supplementary Figure 12

Click here to access/download
Supplementary Material
Supplementary Figure12.pdf

Supplementary Figure 13

Click here to access/download
Supplementary Material
Supplementary Figure13.pdf

Supplementary Figure 14

Click here to access/download

Supplementary Material

Supplementary Figure14.pdf

Supplementary Figure 15

Click here to access/download
Supplementary Material
Supplementary Figure15.pdf

Supplementary Figure 16

Click here to access/download
Supplementary Material
Supplementary Figure16.pdf

Supplementary Figure 17

Click here to access/download
Supplementary Material
Supplementary Figure17.pdf

Supplementary Figure 18

Click here to access/download
Supplementary Material
Supplementary Figure18.pdf

Response to reviewers
Click here to access/download;Personal Cover;Response-to-reviewers_Final.docx

Reviewer reports:

Reviewer #1: A major challenge in single-cell RNA-seq data analysis is the gap between the 'clustering' and 'annotation' steps as pointed out by the authors. The first one is usually driven by feature selections and dimension reduction while the latter is done manually based on biological knowledge. The authors build on the existing clustering method called Seurat to propose a rational selection of a clustering that could approximate the 'best' choice in first instance. By optimizing these steps they want to improve the speed and quality of unsupervised clustering. The classification is evaluated by averaging over the expression values of the ten most differentially expressed genes (ranked by average log fold change) and calculating an error statistic. A decision tree is used to determine the candidate with the lowest classification error. To prove the efficacy of their workflow the authors reproduced the results of three publically available datasets from PBMCs and mouse cortex.

When applied to PBMC, the authors failed to identify T-cell subsets showing the limits of the approach (Figure 2). But, the recursive approach proposed in Figure 3 is very interesting and can be applied to find out more subsets.

I believe the approach is interesting for unknown datasets. Soon the multiplication of dataset annotated (notably in the large consortium like the human cell atlas) will allow to develop machine learning approaches to classify the cells automatically. The authors should discuss this point in the discussion.

What are the major claims of the paper?
The authors claim that by choosing a range of principle components and k values, calculating the clustering and ranking them by gap statistic they can identify the biologically most significant clusters and thus improve the overall quality of the unsupervised clustering as well as reducing the runtime.

Are they novel and will they be of interest to others in the community and the wider field?
The Evaluation of data clustering itself is not novel but it has not been applied for single-cell RNA-seq. Guidelines or algorithms for feature selection are needed and this approach completely removes the need to define the clustering parameters manually.

Is the work convincing, and if not, what further evidence would be required to strengthen the conclusions?
This approach is based on the assumption that clustering and differential gene expression analysis are coupled and no knowledge of the cell types is present. When compared to a more biologically driven workflow that discerns clusters based on canonical marker genes it will rather slow down than speed up the analysis.

On a more subjective note, do you feel that the paper will influence thinking in the field?

In my opinion, the authors are pushing the field into the right direction by evaluating the quality of feature selection and unsupervised clustering.

Ability of a researcher to reproduce the work, given the level of detail provided

The authors have provided a clear and structured GitHub repository that should allow full reproducibility and usability of their work and algorithm, respectively.

We thank the reviewer's positive comments about IKAP. The reviewer finds the recursive approach interesting and has suggested that we discuss about how this approach can be used to classify unknown datasets and how it is related to the growing number of annotated datasets such as the human cell atlas (the corresponding reviewer's comments are highlighted above). We have added relevant discussion in the revised manuscript (page 8, paragraph 2) and described our thoughts briefly below.

Because cell identities are hierarchical in nature (*e.g.* T cell subtypes within T cells), rather than classifying cells into a fixed number of annotated cell types at once, it would be more biologically meaningful to recover the full hierarchy of cell identities. Delineating the hierarchy of cell identities using scRNA-seq data can refine the existing cell ontology [1, 2] and help curate annotations for the reference database such as Human Cell Atlas [3]. Computationally, recovering the hierarchy is essentially a task that recursively identifies major groups as parent identities in the upper layer and finer groups within each major group as child identities in the next layer. In this study, we demonstrate that IKAP recovered the hierarchy of cell identities by constructing a multi-layered ontology for subsets of PBMC datasets and the mouse cortex dataset. Therefore, we believe IKAP can potentially help refine cell ontology and annotate the reference cell identities (such as Human Cell Atlas).

In this version, we have corrected a typo ("pyramidal SS" to "pyramidal S1") and changed the wording for clarity in the revised manuscript. We used differentially expressed (DE) genes for those used to differentiate computationally identified cell groups and reserved "marker genes" for those used to identify manually (or conventionally) annotated cell types. In addition, all major changes in this revised version are highlighted.

Reviewer #2: (NOTE TO READER: I'm using Markdown format below)

Reviewer report

Title: IKAP - Identifying K mAjor cell Population groups in single-cell RNA-seq analysis

Version: Original Submission

Date: June 13, 2019

Review comments to authors:

The authors present a method that elegantly tunes Seurat's clustering parameters to obtain cell clusters that have the best decision tree-classifiability. The method appears to be useful and fast, and by-and-large gives biologically sound results, especially in samples where cells of well-defined type can be expected. I do have some reservations about one of the cases (Mouse brain data by Zeisel et al. 2015) they chose to highlight. The latter may well have prompted the development of a recursive approach which appears to work well and is an interesting extension. I would recommend publication provided the major comments are addressed satisfactorily.

We thank the reviewer for the positive comments overall. We have revised the manuscript based on the reviewer's comments. All major changes are highlighted. We also provide our point-to-point responses below.

Major comments

* Fig. 2D: It is really unsatisfactory that three clearly separated groups (Interneurons, Pyramidal SS and Pyramidal CA1) are lumped together into one "Group 7". The authors should improve and/or explain this behaviour: is it due to their method (and/or Seurat FindAllMarkers) ending up choosing the wrong differentially expressed genes? If so, why? E.g., most of the Interneurons, Pyramidal SS and Pyramidal CA1 cells are perfectly recognizable in the heatmap (big yellow blocks), were it not for the Atp1a3, Ndrg4 and Stmn3 obscuring things. The latter 3 genes are probably selected based on their strong under-expression in groups 1 and 2. A number of things could be going on:

1. The decision tree approach yields a poor classifier that badly influences the choice of DE genes. It may not be much work to use a different classifier such as SVM, or even tweaking the parameters given to FindAllMarkers.

2. The authors developed and optimized the method based on the first big 10X Genomics data set, and as a result the method does not generalize well enough. In my experience, the 10X data set is not so easy to classify. Most current cell type classification methods have difficulties with it, see Abdelaal et al. 2019, BioRxiv https://doi.org/10.1101/644435.). It could be remedied by applying their

method to another data set, e.g. any of the cancer data sets from the Regev lab (with or without the malignant cells).

3. Confounding factors are at play. It might help to first filter out mitochondrial(ly-related) and ribosomal protein genes and all genes related to the cell cycle before doing anything else.

The reviewer is concerned that IKAP lumps the 3 cell types: interneurons, pyramidal CA1, and pyramidal S1 (please note that we corrected the typo "pyramidal SS" to "pyramidal S1" in the revision), together due to some unknown issues that need to be fixed because the 3 types are clearly separated groups with distinct marker genes. The reviewer also suspects that the 3 DE genes of the union group of the 3 cell types identified by IKAP were picked because of strong under-expression in group 1 and 2.

We thank the reviewer for providing insights on what could go wrong in the analysis. However, the union group identified by IKAP represents a high-level cell identity shared across interneurons, pyramidal CA1, and pyramidal S1. To support this, we have now added a supplementary figure in the revised manuscript showing that the top 10 genes with high AUROC that classify cells into the union group versus others are highly expressed in the 3 cell types but exhibit low (or no) expression in other types (Supplementary Figure 9). Then, we conducted Wilcoxon rank sum tests showing that all these 10 genes are significantly upregulated in each of the 3 cell types compared with each of the other cell types ($P < 1E-30$ for all tests). This shows that their differential expression was not simply due to strong under-expression in groups 1 and 2 (see Supplementary Table 1). In addition, these 3 cell types are also shown to be close to each other compared to other types in the clustering result in the original paper (see Figure 1C in [4]). These data support that although the 3 cell types expressed distinct marker genes, they also shared certain similarity in gene expression, implicating a potential common cell identity. We have added the analyses mentioned above in the main texts (starting from page 6, line 122).

The reviewer's comment also raises a key issue that it is not always clear as to what constitutes a cell type. For example, in the PBMC datasets, some people look for T cells whereas others look for T cell subtypes such as CD4+ and CD8+ T cells. All T cells express T cell marker genes but T cell subtypes are also differentiated by subtype marker genes. A similar case is seen for the union group versus the 3 cell types in the mouse cortex dataset. Because cell identities are hierarchical in nature, cell types can be presumably defined at different levels of resolution. To obtain the full picture of cell identities, we believe delineating the hierarchy of cell identities would be more biologically meaningful than classifying cells into a fixed number of reference cell types. Computationally, recovering the hierarchy is essentially a task that recursively identifies major groups as parent identities in the upper layer and finer groups within each major group as child identities in the next layer. We have shown that IKAP recovered the hierarchy of cell identities for subsets of PBMC and the mouse cortex datasets by recursively running it within each major group. We believe IKAP would be helpful for refining cell ontology and curating reference cell identities using scRNA-seq datasets in the future. We have added discussion for the points mentioned above in the main texts (page 8, paragraph 2).

To avoid ambiguity, in the revised manuscript, we use “cell type” for a group of cells that has been manually annotated and defined by a set of marker genes. This clarification has been added in page 5, line 86.

* The authors should point out (e.g. at the end of the Findings section) that their approach is only suitable for samples where a limited number of definite and distinct cell types can be expected. Their method forces cells into discrete groups, each with a maximally distinct gene expression profile. This will not work in cases with many unknown cells (e.g. tumor samples) or cases where gradients can be expected (time courses, developmental studies).

As suggested by the reviewer, we have added a paragraph that lists the concerns (including this one) when using IKAP (see page 9, paragraph 2).

* How were the AUROC's calculated? Details in the Performance Summary (page 13) are too few to reproduce.

We thank the reviewer for pointing out the missing detail. We have now added more details as follow: “For each DE gene, the AUROC (Area Under the ROC curve) was computed for classifying its associated group versus others using normalized UMI count and the function *roc.curve* in the R package PRROC.” (see page 13, line 268)

Suggestions

* From Suppl. Fig1 it would appear that testing each number of PC's is often superfluous, skipping a few could save time

* I strongly suggest making your package (also) compatible with Seurat 3.x, which is now the default and is likely to be used much more widely

We thank the reviewer’s suggestions about how to speed up IKAP and the compatibility of the latest Seurat package. We have already implemented a new version of IKAP compatible with Seurat v3. Now it is under the beta testing. We will release it as soon as the testing is completed. We are also compiling ideas to improve IKAP for our next release. This includes speeding up IKAP by skipping calculation of a few PCs.

Minor (editorial) comments:

* throughout the text:
  * a quick read-through by a native speaker should help to fix the missing "the"'s.

We have fixed the issue as requested by the reviewer.

* the authors should clarify the distinction between differentially expressed (DE) genes (which are inferred, as here, between groups of cells) and marker genes, which are consensus genes whose products are conventionally used to identify cells. It appears that in most occurrences of this term, DE genes are meant, rather than marker genes.

As suggested by the reviewer, we have replaced “marker genes” with “DE genes” throughout the text when it is meant to be the genes differentially expressed between two computationally identified cell groups and clarified that marker genes are used to define a manually (or conventionally) annotated cell type (see page 5, line 86).

* the terms "feature list" and "choice of features" are in the machine-learning world known as "feature selection" and "feature set". Please change for clarity.

Changed as suggested by the reviewer.

* provide references for all the R packages used (those for rpart and PRROC are missing)

Both were added.

* Fig. 1, step 4, heatmap: biggest gap increase for nPC=9 visually appears to be for k=5->6, not for k=6->7 as indicated; perhaps adjust the color scale

* Fig. 2C: highlight the fact that chosen 3 parameters (3 leftmost columns) are contrasted with "trial-and-error strategy", e.g. by separating them horizontally a bit. Clarify that it need not be 3 candidates and one 'best'

* Fig. 2: what does the horizontal dashed line signify?

We thank the reviewer pointing out these details to improve the figures. We have adjusted Figures 1 and 2 and added description to the legend for Figure 2.

(Page numbers below refer to those in the reviewer document)

* page 6:
  * "... two user-specified parameters: resolution r ..." This is a unclear, these are Seurat parameters which IKAP doesn't get, but infers itself.

We have changed the wording.

* " To simulate the coarse-to-fine grouping process ": should be "fine-to-coarse" as merging leads to ever-larger clusters

Changed.

* "For each set, gap statistic is" : insert "the". Authors should mention that Tibshirani's additional rule for selecting the 'best' k is not used here (why not?)

We have added “the”, mentioned that Tibshirani's rule of selecting the best *k* is not used (see page 12, line 231), and briefly explained why the rule is not used in the discussion (see page 9, line 169).

* "Finally, candidate sets grouped": clarify (apparently authors mean sets of k and nPC)

We have changed the wording to make it clear.

* reference [5]: incomplete reference, provide full download details

Added url to the download page.

* "marked as the best using decision trees" -> "marked as the best, using decision trees"

Changed.

* page 7:
  * "such that cell lineage" -> "and cell lineage"

Changed.

* page 12:
  * "each group in a candidate set": clarify what a candidate set is.

We have moved the original step 7 to step 4 in the revised version and made some changes in the Methods to make it clear (see page 12, step 4).

* page 13:
  * "IKAP utilizes FindAllMarkers": insert "the"

Added.

* "is marked as the best and the rest are alternatives": How many 'alternatives' are there typically?

Based on the experiments we have tried, the number of candidate sets (including the best and the alternatives) reported by IKAP usually range from 1 to 4. We have added this information in page 13, line 266.

Reference:

1. Aevermann BD, Novotny M, Bakken T, Miller JA, Diehl AD, Osumi-Sutherland D, et al. Cell type discovery using single-cell transcriptomics: implications for ontological representation. Hum Mol Genet. 2018;27(R1):R40-R7. doi:10.1093/hmg/ddy100
2. Bakken T, Cowell L, Aevermann BD, Novotny M, Hodge R, Miller JA, et al. Cell type discovery and representation in the era of high-content single cell phenotyping. BMC Bioinformatics. 2017;18(Suppl 17):559. doi:10.1186/s12859-017-1977-1
3. Regev A, Teichmann SA, Lander ES, Amit I, Benoist C, Birney E, et al. The Human Cell Atlas. Elife. 2017;6. doi:10.7554/eLife.27041
4. Zeisel A, Munoz-Manchado AB, Codeluppi S, Lonnerberg P, La Manno G, Jureus A, et al. Brain structure. Cell types in the mouse cortex and hippocampus revealed by single-cell RNA-seq. Science. 2015;347(6226):1138-42. doi:10.1126/science.aaa1934

Cover letter Click here to access/download;Personal Cover;Cover_letter.docx

DEPARTMENT OF HEALTH & HUMAN SERVICES

Bioinformatics and Computational Biology
National Heart Lung and Blood Institute
National Institutes of Health
https://bioinformatics.nhlbi.nih.gov/
Tel: 301-451-2772
BG 12A RM 3053N
12 SOUTH DR.
Bethesda MD 20892

NIH National Heart, Lung, and Blood Institute

April 22, 2019

Dear Dr. Edmunds,

We are pleased to submit our article, “IKAP - Identifying K mAjor cell Population groups in single-cell RNA-seq analysis,” to be considered for publication in *GigaScience as a Technical Note*.

Identifying cell types is the most important step in single-cell RNA-sequencing (scRNA) analysis achieved by clustering cells into cell groups and recognizing the cell type for each cell group based on its differentially expressed marker genes. The distinguishing power of those marker genes determines the effectiveness of cell type recognition. Although cell groups resulting from clustering greatly affect the marker genes that can be identified for each group, existing scRNA analysis packages do not consider this dependency but perform the two steps independently by clustering cell groups based on user-specified parameters followed by marker gene identification. This often becomes a bottleneck, in which users need to try different sets of parameters to get biologically meaningful cell groups.

In this study, we developed an algorithm (called IKAP) on top of Seurat – one of the most widely used scRNA analysis packages – to automatically identify major cell groups that produce distinguishing marker genes without any user-specified parameters. We tested IKAP on several datasets including two PBMC datasets (~4K and ~8K cells) and a mouse cortex dataset (~3K) and showed that IKAP accurately clustered cells into groups consistent with cell lineages such as B cells and T cells in PBMC datasets and identified cell groups consistent with previously annotated cell types in the mouse cortex dataset. In addition, we also designed quantitative metrics to show cell groups reported by IKAP produce distinguishing marker genes. Finally, we demonstrate IKAP can automate cell ontology curation using scRNA data, which is an emerging focus of this field as more and more scRNA data are generated. Therefore, we believe IKAP would be an extremely valuable tool to be added to the scRNA analysis toolbox.

This manuscript is not under consideration for publication elsewhere. All authors have read the manuscript and approve of the work. We do not have any conflicts of interest to disclose.

Thank you for your consideration. We look forward to hearing from you.

Sincerely,
Yun-Ching Chen and Mehdi Pirooznia
