# Supplementary material for: IKAP—Identifying K mAjor cell Population groups in single-cell RNA-sequencing analysis
Source: Gigascience. 2019 Oct 1;8(10):giz121. doi: 10.1093/gigascience/giz121 (PMC6771546; doi:10.1093/gigascience/giz121)
Supplement: giz121_Supplemental_Files [file giz121_supplemental_files.zip › Supplementary Figure12.pdf]

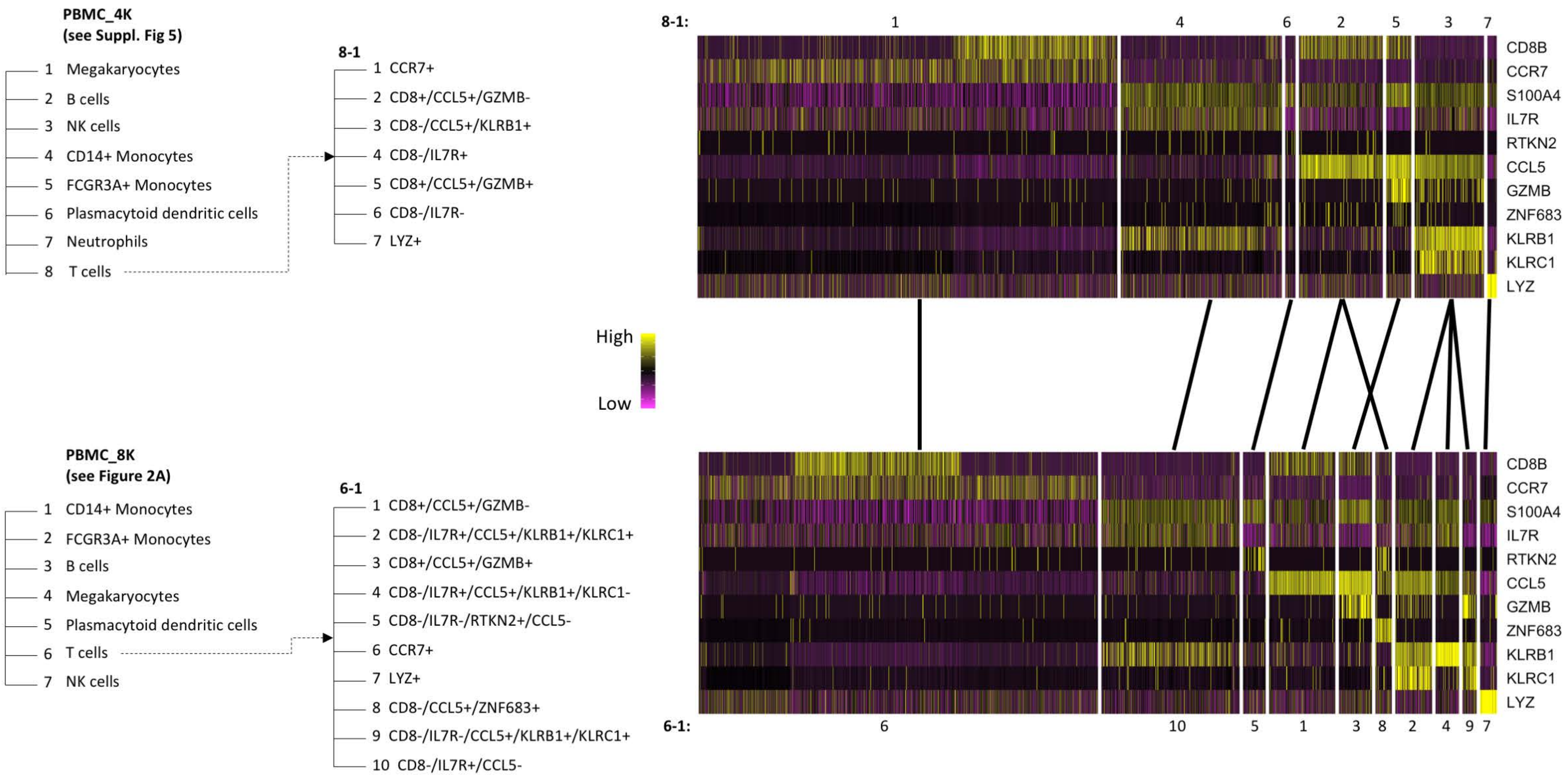
PBMC_4K
(see Suppl. Fig 5)
1 Megakaryocytes
2 B cells
3 NK cells
4 CD14+ Monocytes
5 FCGR3A+ Monocytes
6 Plasmacytoid dendritic cells
7 Neutrophils
8 T cells
8-1
1 CCR7+
2 CD8+/CCL5+/GZMB-
3 CD8-/CCL5+/KLRB1+
4 CD8-/IL7R+
5 CD8+/CCL5+/GZMB+
6 CD8-/IL7R-
7 LYZ+
8-1:
1
4
6
2
5
3
7
CD8B
CCR7
S100A4
IL7R
RTKN2
CCL5
GZMB
ZNF683
KLRB1
KLRC1
LYZ
High
Low
PBMC_8K
(see Figure 2A)
1 CD14+ Monocytes
2 FCGR3A+ Monocytes
3 B cells
4 Megakaryocytes
5 Plasmacytoid dendritic cells
6 T cells
7 NK cells
6-1
1 CD8+/CCL5+/GZMB-
2 CD8-/IL7R+/CCL5+/KLRB1+/KLRC1+
3 CD8+/CCL5+/GZMB+
4 CD8-/IL7R+/CCL5+/KLRB1+/KLRC1-
5 CD8-/IL7R-/RTKN2+/CCL5-
6 CCR7+
7 LYZ+
8 CD8-/CCL5+/ZNF683+
9 CD8-/IL7R-/CCL5+/KLRB1+/KLRC1+
10 CD8-/IL7R+/CCL5-
CD8B
CCR7
S100A4
IL7R
RTKN2
CCL5
GZMB
ZNF683
KLRB1
KLRC1
LYZ
6-1:
6
10
5
1
3
8
2
4
9
7
