# Supplementary material for: IKAP—Identifying K mAjor cell Population groups in single-cell RNA-sequencing analysis
Source: Gigascience. 2019 Oct 1;8(10):giz121. doi: 10.1093/gigascience/giz121 (PMC6771546; doi:10.1093/gigascience/giz121)
Supplement: giz121_Supplemental_Files [file giz121_supplemental_files.zip › Supplementary Figure18.pdf]

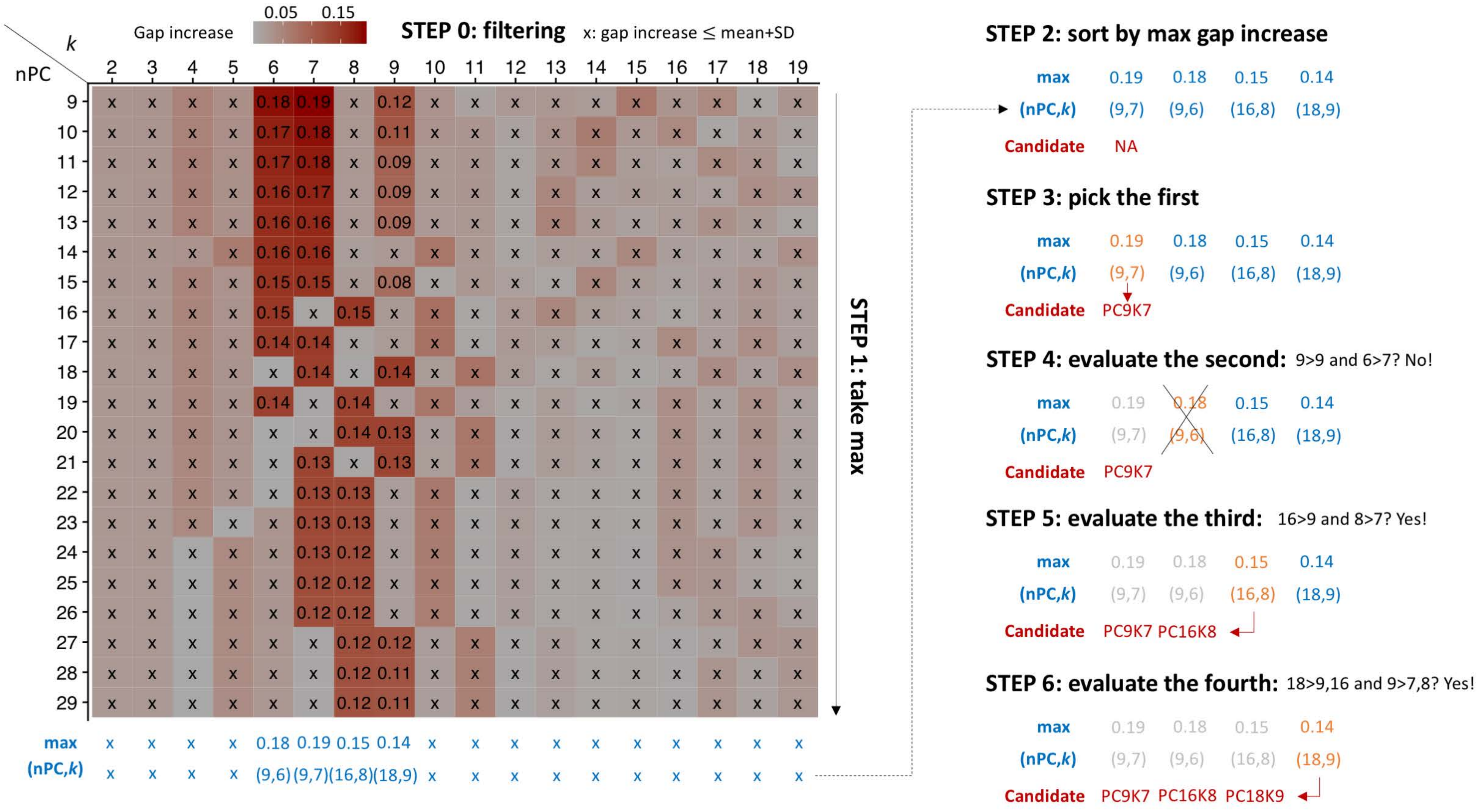

Gap increase
0.05 0.15
STEP 0: filtering
x: gap increase ≤ mean+SD
k
nPC
2 3 4 5 6 7 8 9 10 11 12 13 14 15 16 17 18 19
9 10 11 12 13 14 15 16 17 18 19 20 21 22 23 24 25 26 27 28 29
max x x x x 0.18 0.19 0.15 0.14 x x x x x x x x x x
(nPC,k) x x x x (9,6) (9,7) (16,8) (18,9) x x x x x x x x x x
STEP 1: take max
STEP 2: sort by max gap increase
max 0.19 0.18 0.15 0.14
(nPC,k) (9,7) (9,6) (16,8) (18,9)
Candidate NA
STEP 3: pick the first
max 0.19 0.18 0.15 0.14
(nPC,k) (9,7) (9,6) (16,8) (18,9)
Candidate PC9K7
STEP 4: evaluate the second: 9>9 and 6>7? No!
max 0.19 0.18 0.15 0.14
(nPC,k) (9,7) (9,6) (16,8) (18,9)
Candidate PC9K7
STEP 5: evaluate the third: 16>9 and 8>7? Yes!
max 0.19 0.18 0.15 0.14
(nPC,k) (9,7) (9,6) (16,8) (18,9)
Candidate PC9K7 PC16K8
STEP 6: evaluate the fourth: 18>9,16 and 9>7,8? Yes!
max 0.19 0.18 0.15 0.14
(nPC,k) (9,7) (9,6) (16,8) (18,9)
Candidate PC9K7 PC16K8 PC18K9
