# Supplementary material for: IKAP—Identifying K mAjor cell Population groups in single-cell RNA-sequencing analysis
Source: Gigascience. 2019 Oct 1;8(10):giz121. doi: 10.1093/gigascience/giz121 (PMC6771546; doi:10.1093/gigascience/giz121)
Supplement: giz121_Supplemental_Files [file giz121_supplemental_files.zip › Supplementary Table 1.docx]

Supplementary Table 1. Median expression of genes significantly^*^ upregulated in the union of interneurons, pyramidal S1, and pyramidal CA1 for the mouse cortex cell types annotated in Zeisel *et al*., 2015^#^.

|  | interneurons | pyramidal S1 | pyramidal CA1 | oligodendrocytes | microglia | endothelial-mural | astrocytes-ependymal |
| --- | --- | --- | --- | --- | --- | --- | --- |
| Atp1b1 | 3.64 | 3.08 | 3.54 | 0 | 1.15 | 1.36 | 1.6 |
| Stmn3 | 2.97 | 2.86 | 2.83 | 0 | 0 | 0 | 0 |
| Rtn1 | 2.99 | 2.78 | 3.1 | 0 | 0.39 | 0 | 0.69 |
| Atp1a3 | 2.4 | 1.79 | 2.36 | 0 | 0 | 0 | 0 |
| Ndrg4 | 2.78 | 2.36 | 2.18 | 0 | 0 | 0 | 0 |
| Nsf | 2.37 | 2.21 | 2.48 | 0 | 0 | 0 | 0 |
| Eno2 | 2.1 | 2.13 | 2.24 | 0 | 0 | 0 | 0 |
| Thy1 | 1.77 | 1.84 | 2.04 | 0 | 0 | 0 | 0 |
| Syp | 1.66 | 1.71 | 1.68 | 0 | 0 | 0 | 0 |
| Rab3a | 2.07 | 1.8 | 1.8 | 0 | 0 | 0 | 0 |

^*^All genes were significantly upregulated (Wilcoxon rank sum test P-value < 1E-30) in each of interneurons, pyramidal S1, and pyramidal CA1 compared with each of oligodendrocytes, microglia, endothelial-mural, and astrocytes-ependymal.

^#^Zeisel A, Munoz-Manchado AB, Codeluppi S, Lonnerberg P, La Manno G, Jureus A, et al. Brain structure. Cell types in the mouse cortex and hippocampus revealed by single-cell RNA-seq. Science. 2015;347(6226):1138-42. doi:10.1126/science.aaa1934
