# Supplementary figures and images for: IKAP—Identifying K mAjor cell Population groups in single-cell RNA-sequencing analysis
Source: Gigascience. 2019 Oct 1;8(10):giz121. doi: 10.1093/gigascience/giz121 (PMC6771546; doi:10.1093/gigascience/giz121)

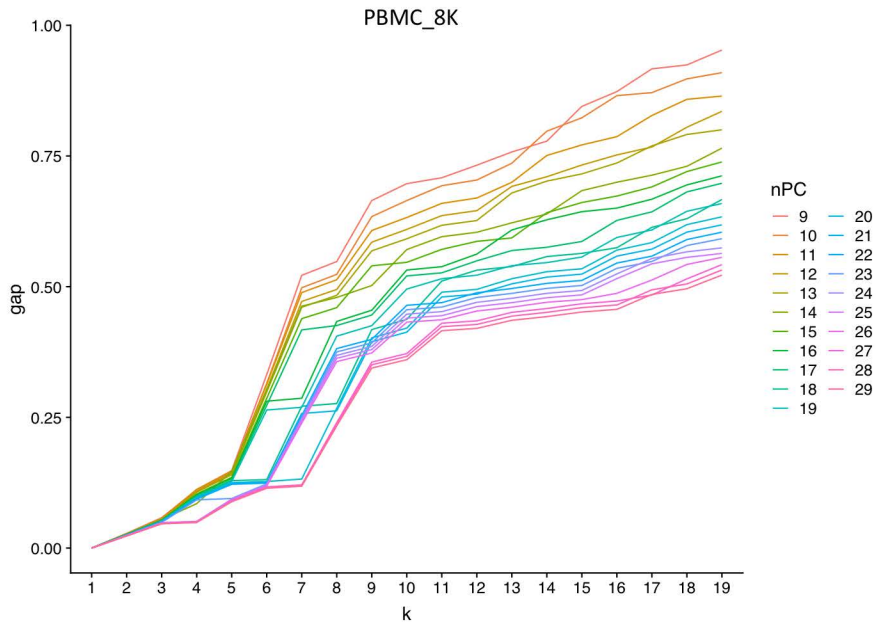

Supplement: giz121_Supplemental_Files [file giz121_supplemental_files.zip › Supplementary Figure 1.pdf]

High  
Low

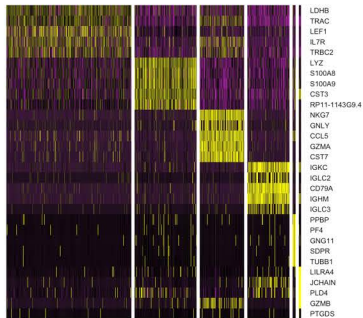

nPC=5, r=0.1

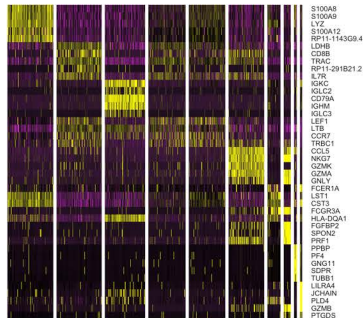

nPC=5, r=0.4

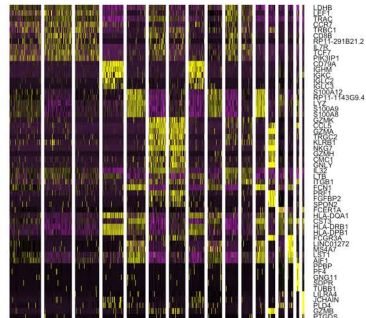

nPC=5, r=1.0

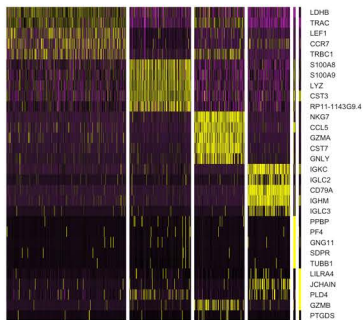

nPC=15, r=0.1

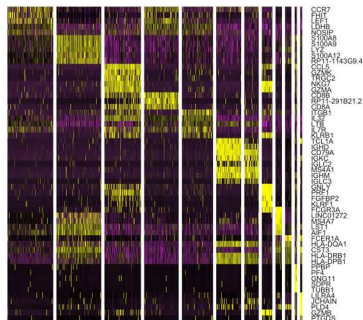

nPC=15, r=0.4

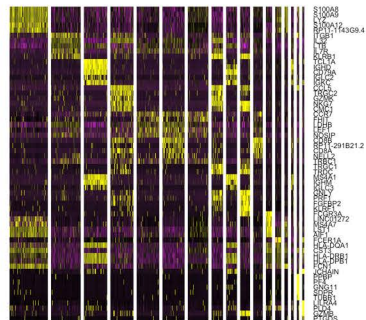

nPC=15, r=1.0

Supplement: giz121_Supplemental_Files [file giz121_supplemental_files.zip › Supplementary Figure 3.pdf]

PC16K8

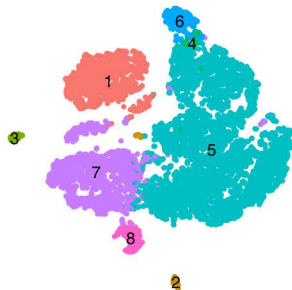

PC18K9

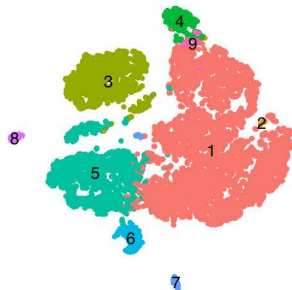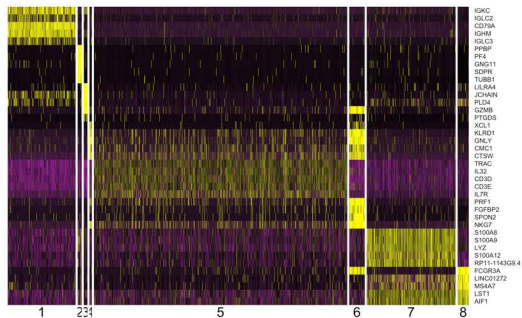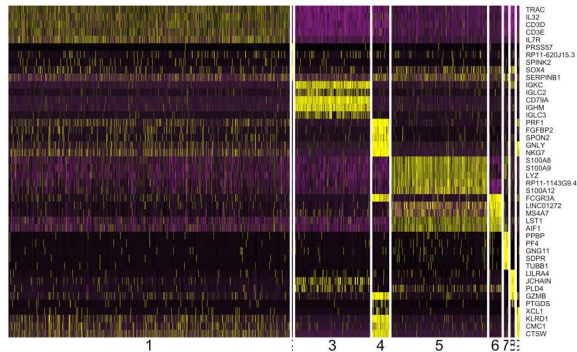

Supplement: giz121_Supplemental_Files [file giz121_supplemental_files.zip › Supplementary Figure 4.pdf]

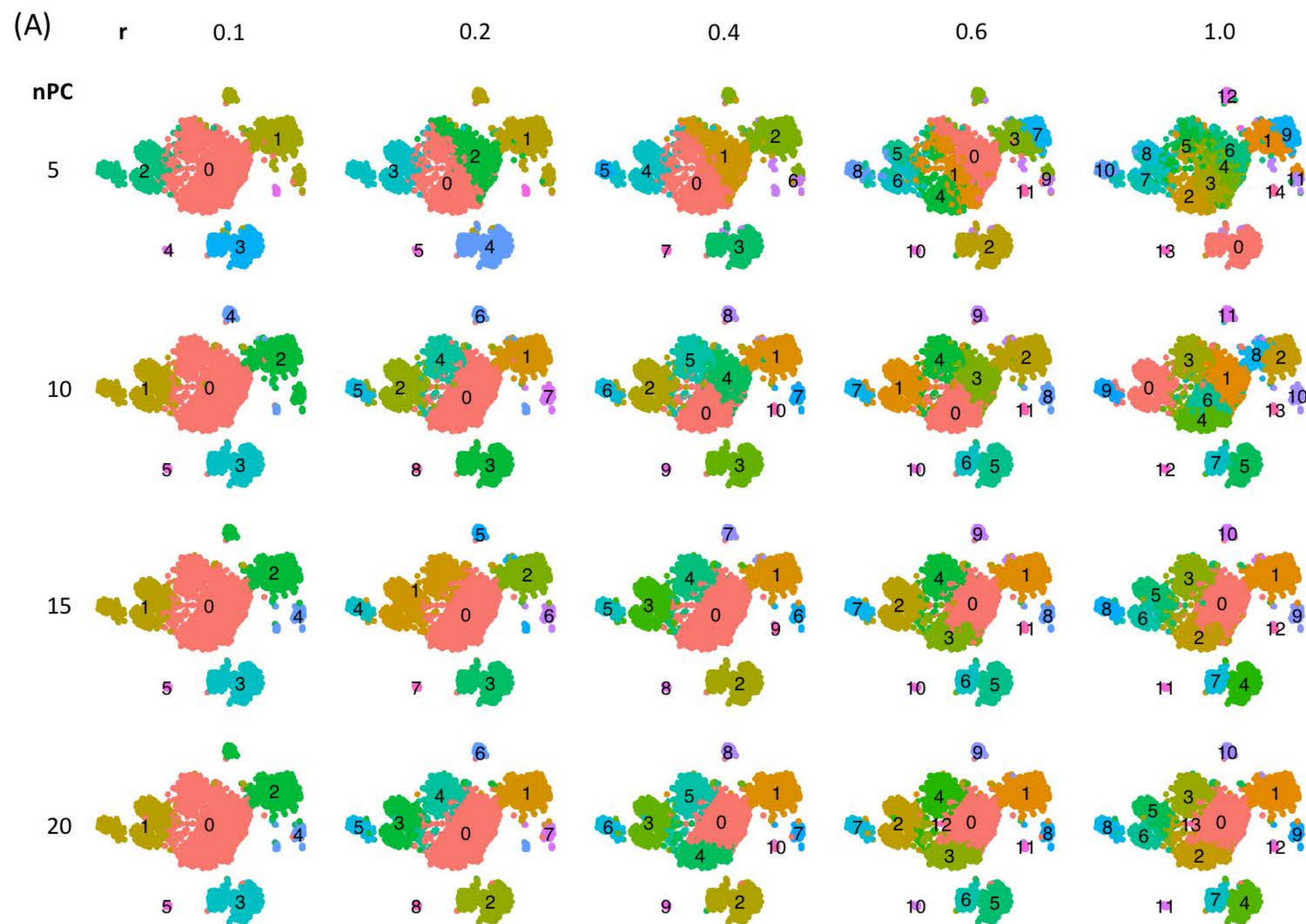

(B)

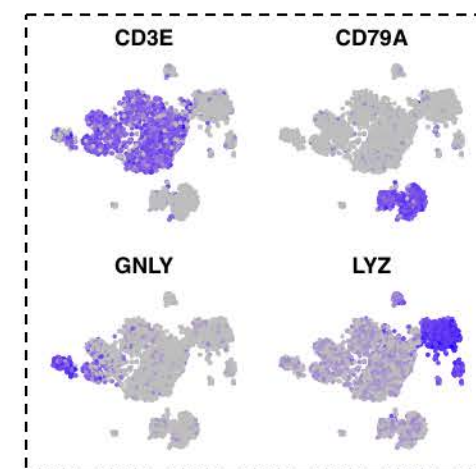

Supplement: giz121_Supplemental_Files [file giz121_supplemental_files.zip › Supplementary Figure 6.pdf]

High  
Low

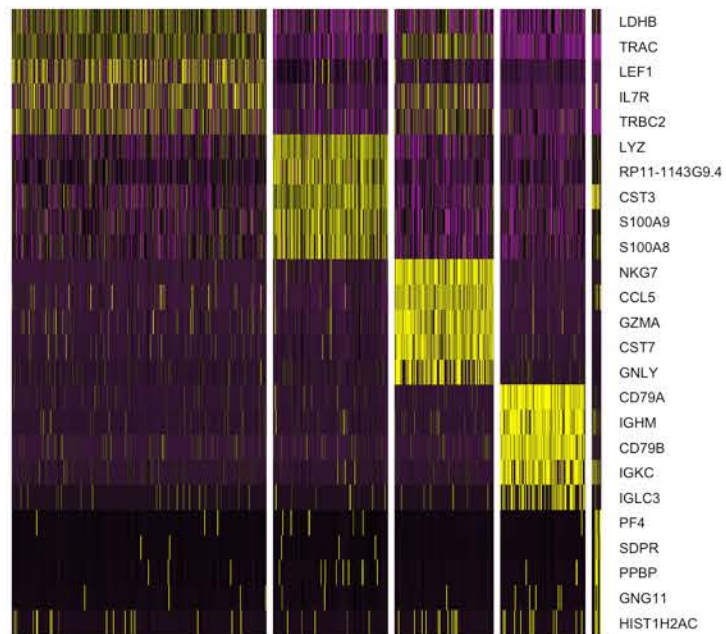

nPC=5, r=0.1

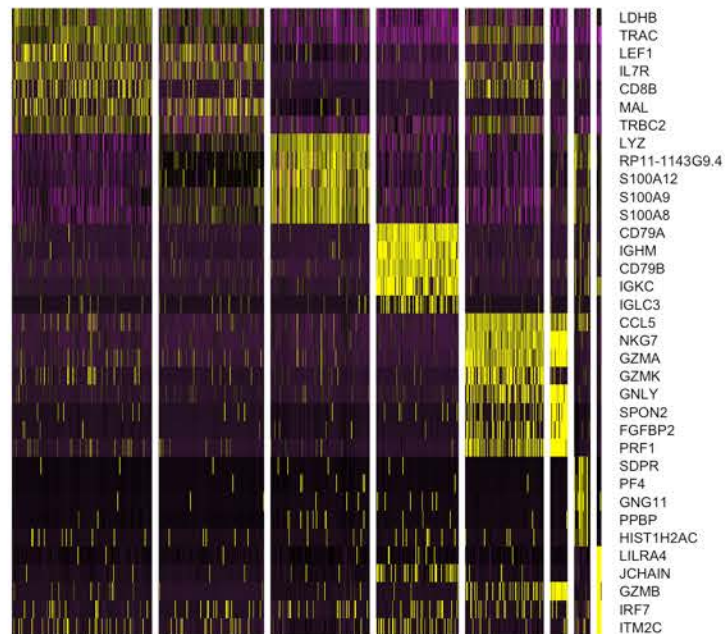

nPC=5, r=0.4

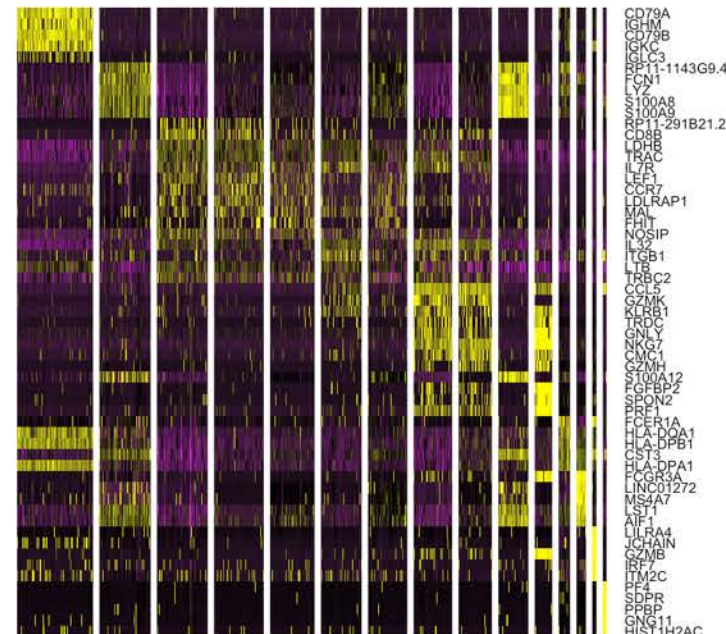

nPC=5, r=1.0

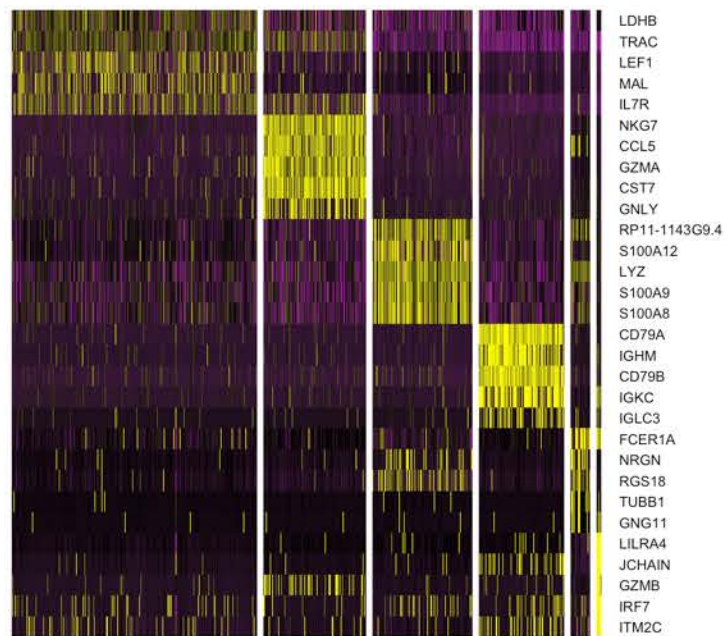

nPC=15, r=0.1

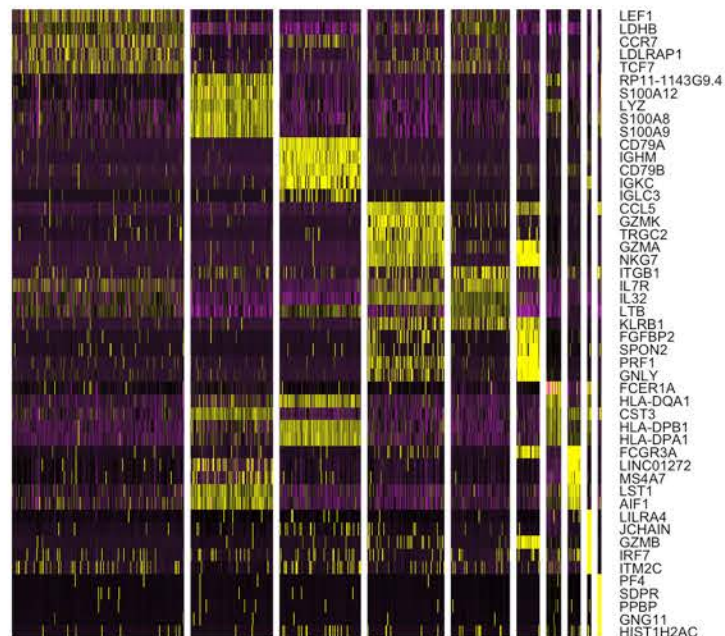

nPC=15, r=0.4

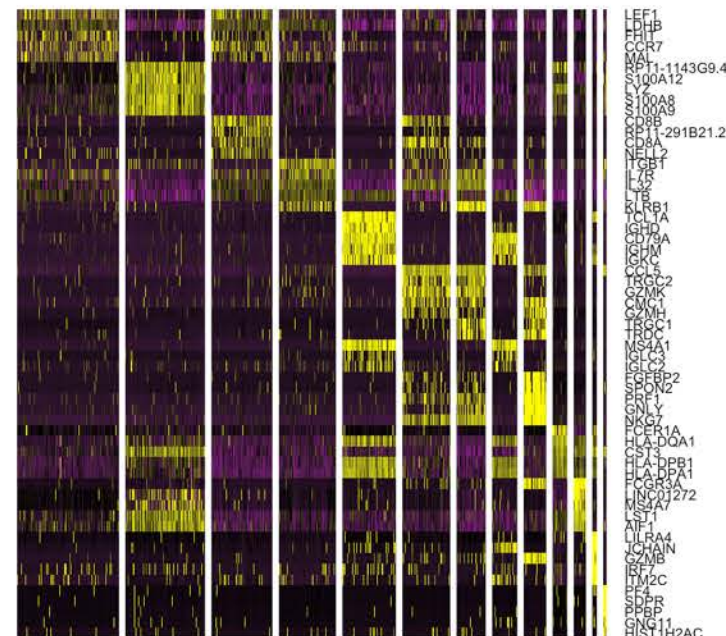

nPC=15, r=1.0

Supplement: giz121_Supplemental_Files [file giz121_supplemental_files.zip › Supplementary Figure 7.pdf]

**PBMC\_4K**  
(see Suppl. Fig X)

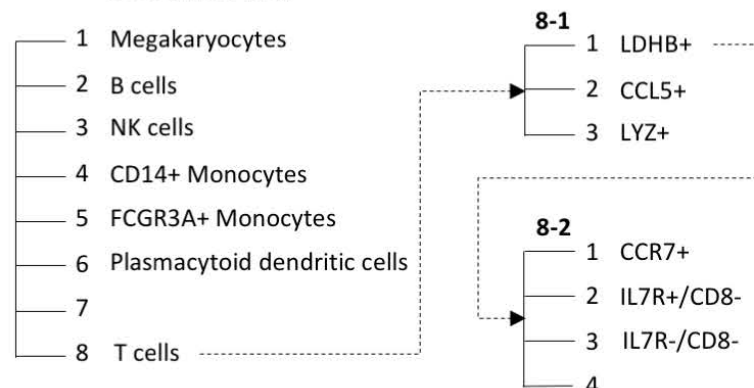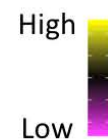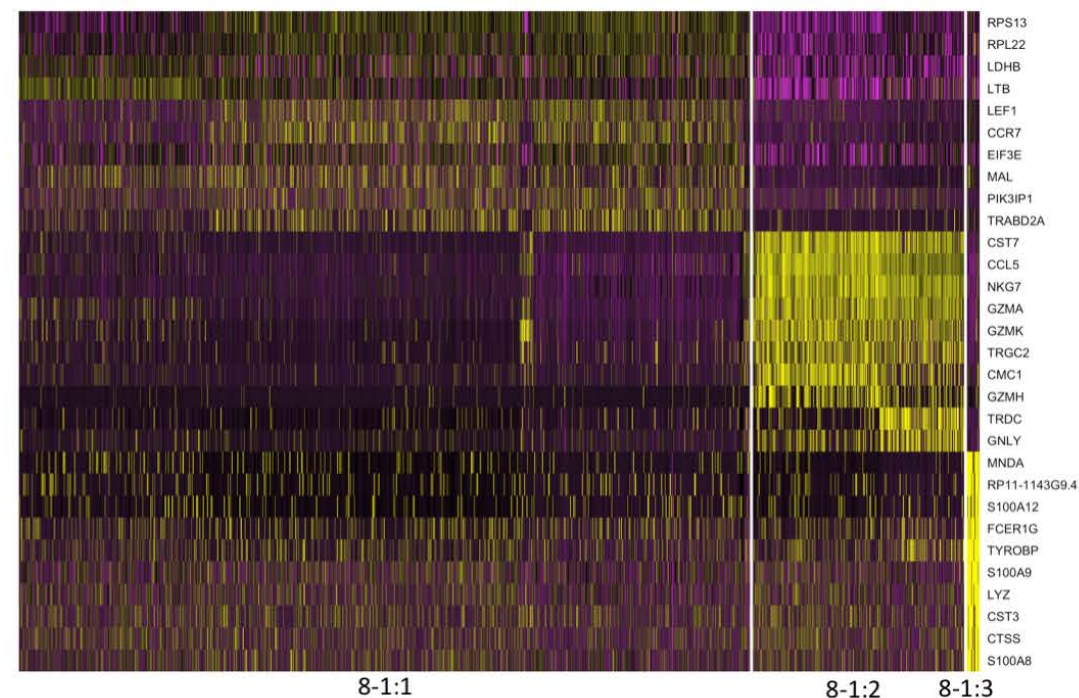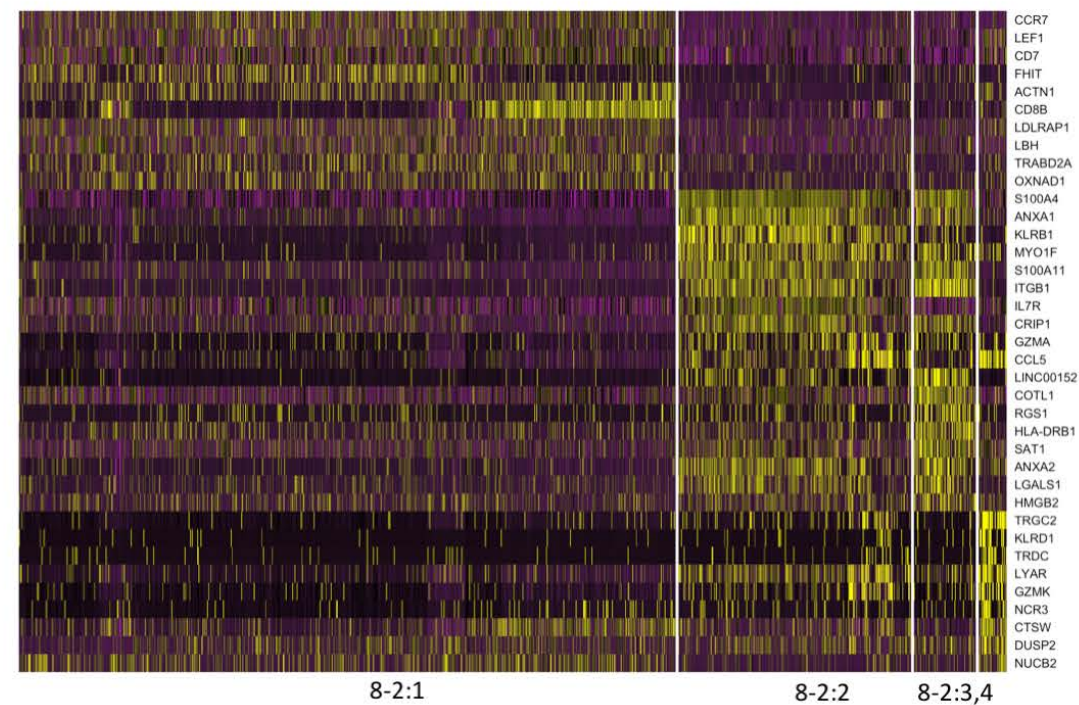

Supplement: giz121_Supplemental_Files [file giz121_supplemental_files.zip › Supplementary Figure10.pdf]

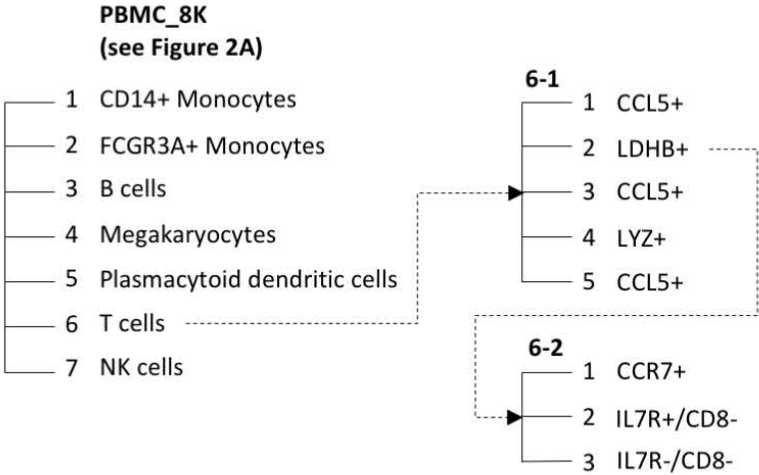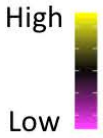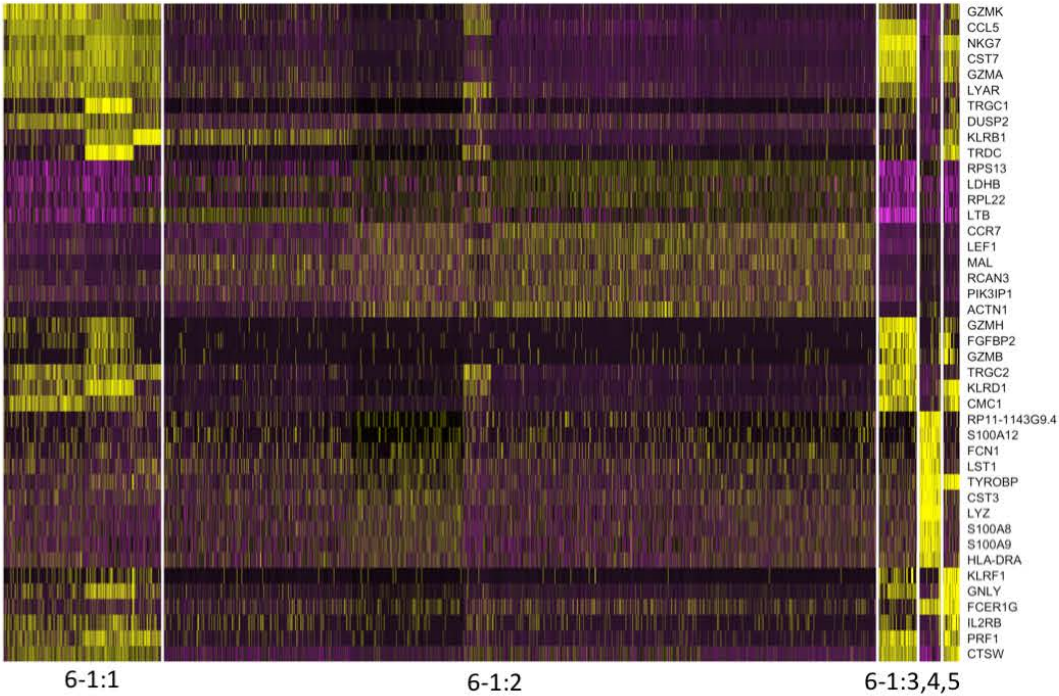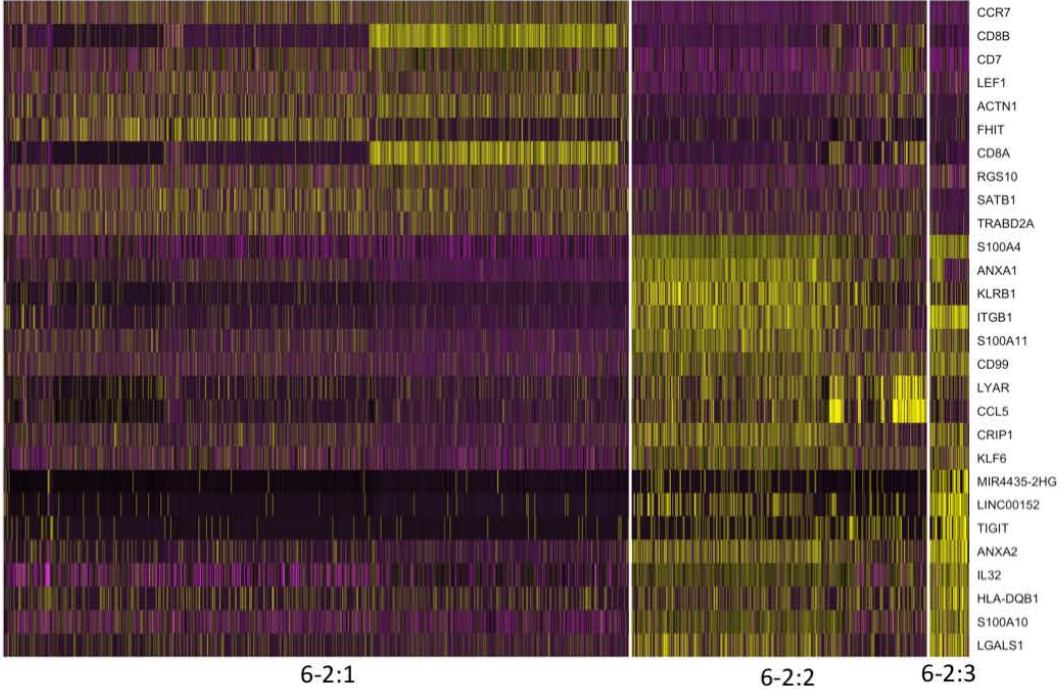

Supplement: giz121_Supplemental_Files [file giz121_supplemental_files.zip › Supplementary Figure11.pdf]

PBMC\_4K

Expression of top 10  
marker genes for 7 T  
cell subgroups

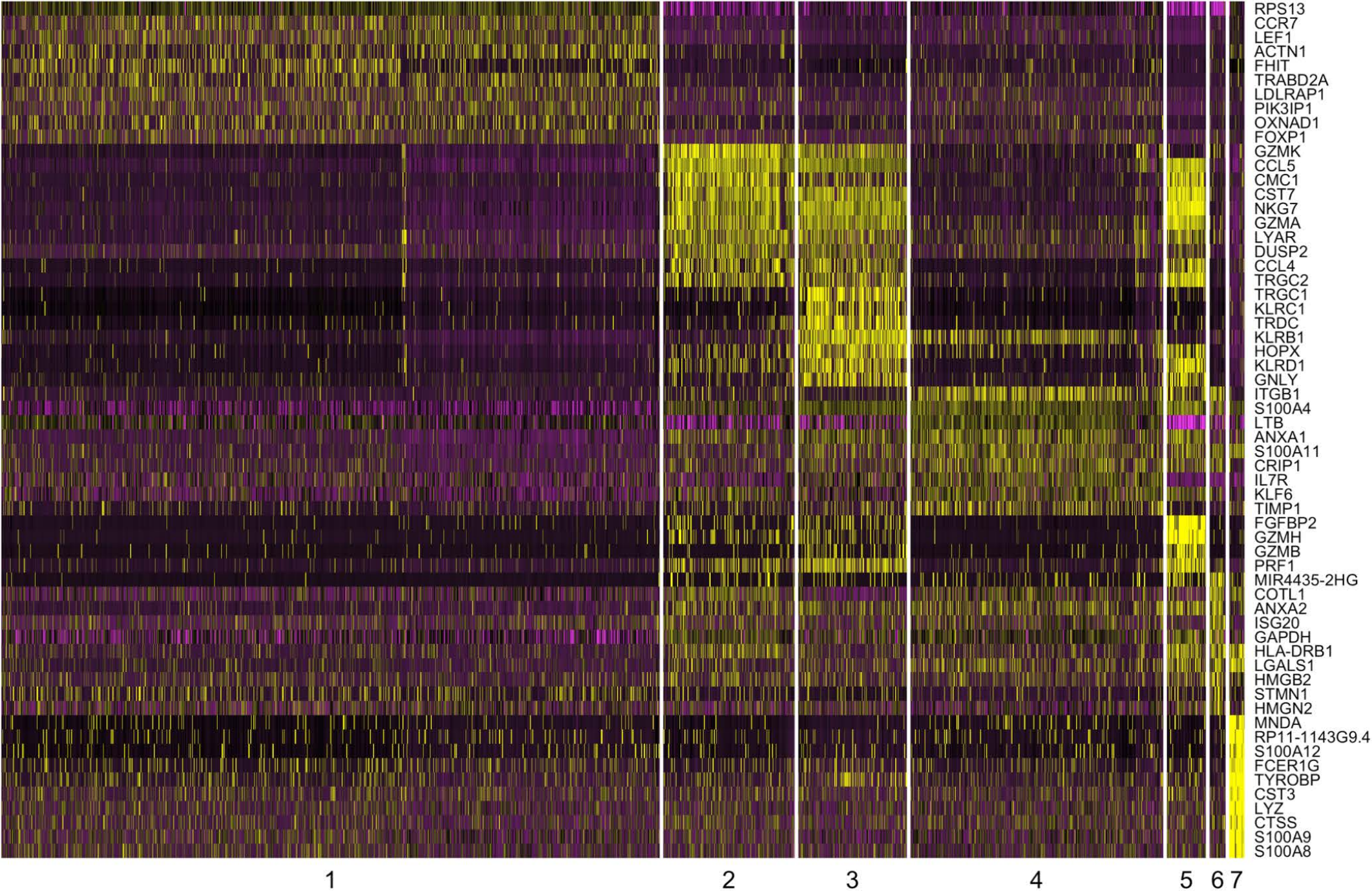

Supplement: giz121_Supplemental_Files [file giz121_supplemental_files.zip › Supplementary Figure13.pdf]

PBMC\_8K

Expression of top 10  
marker genes for 10 T  
cell subgroups

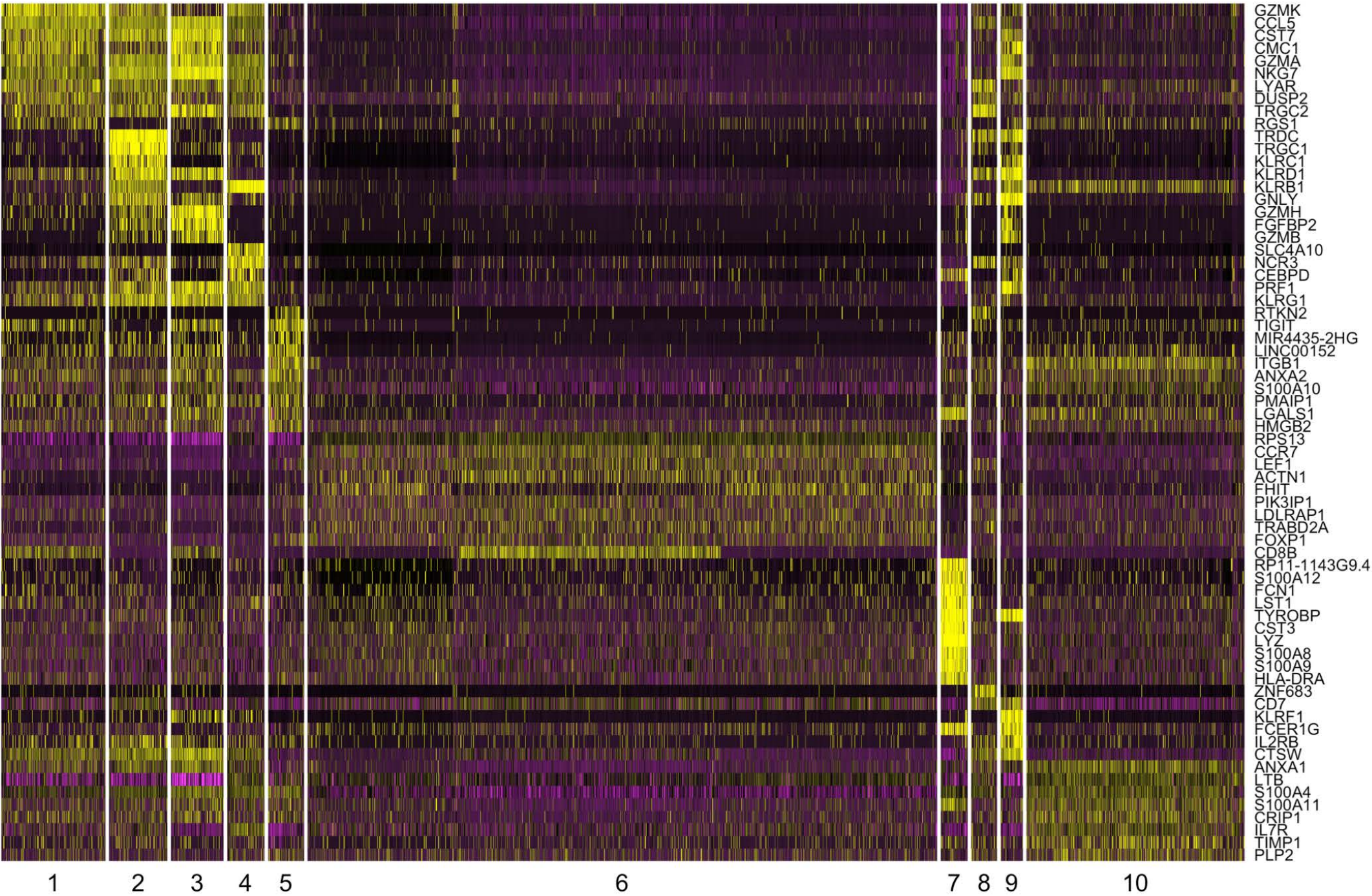

Supplement: giz121_Supplemental_Files [file giz121_supplemental_files.zip › Supplementary Figure14.pdf]

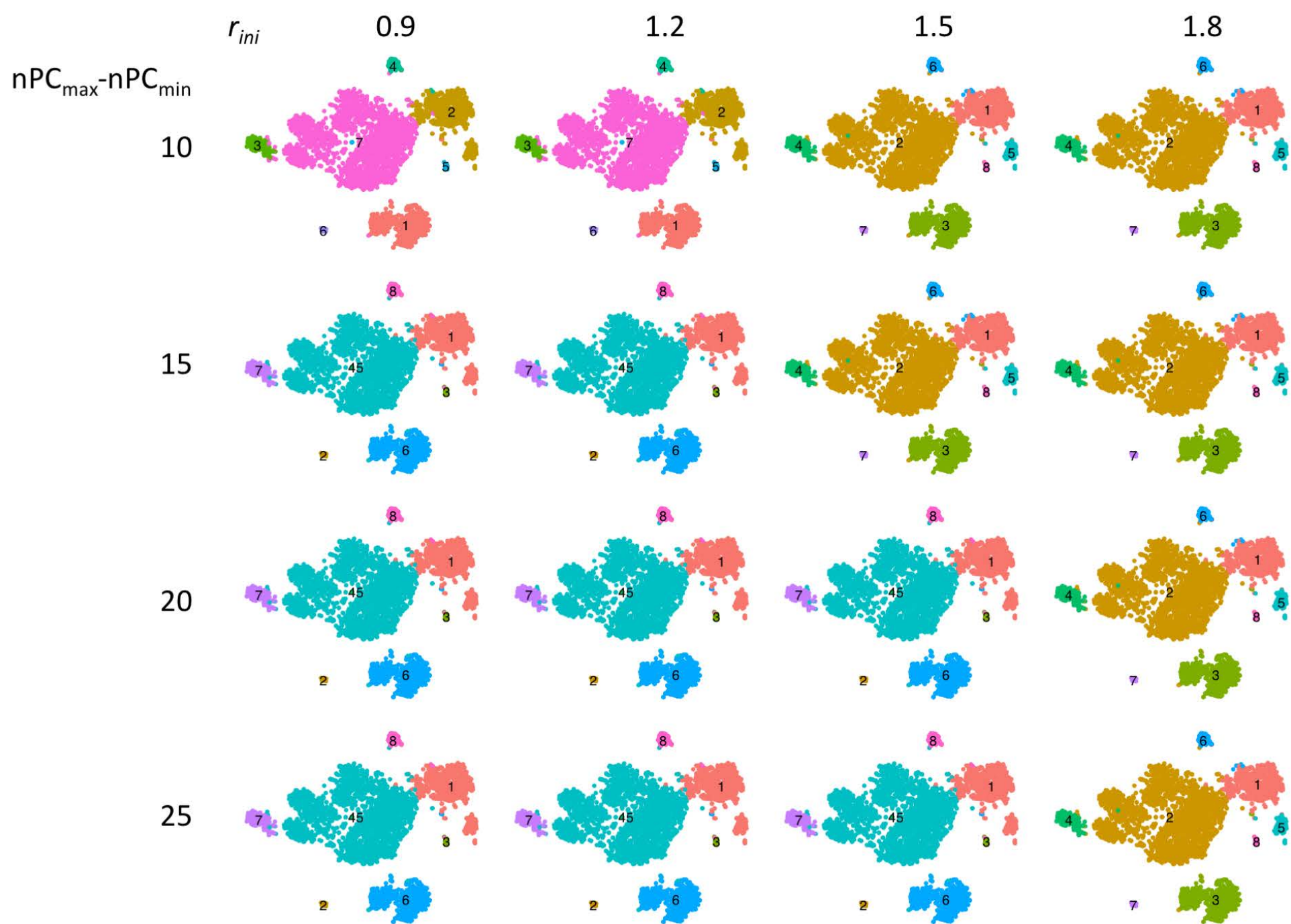

Supplement: giz121_Supplemental_Files [file giz121_supplemental_files.zip › Supplementary Figure15.pdf]

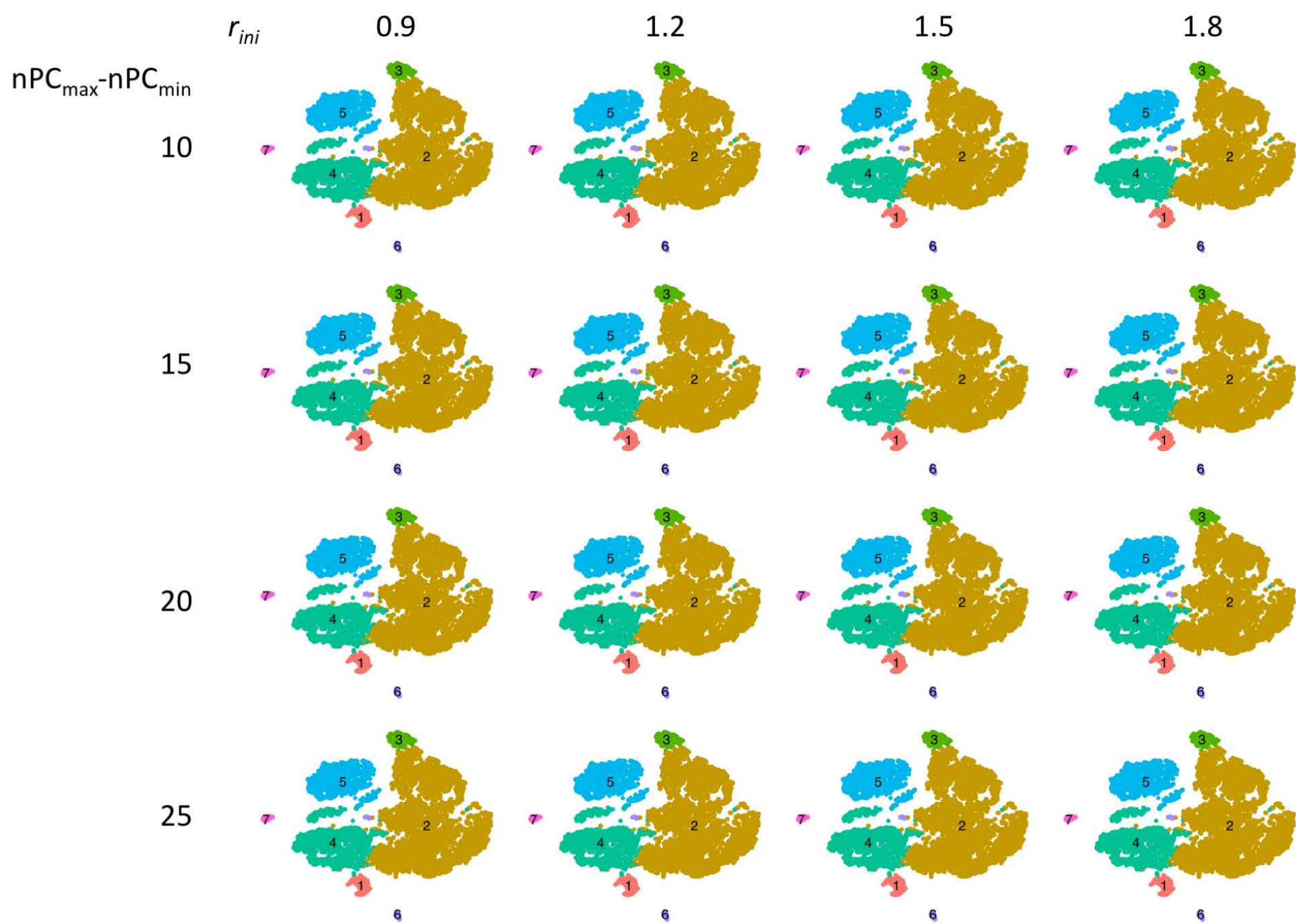

Supplement: giz121_Supplemental_Files [file giz121_supplemental_files.zip › Supplementary Figure16.pdf]

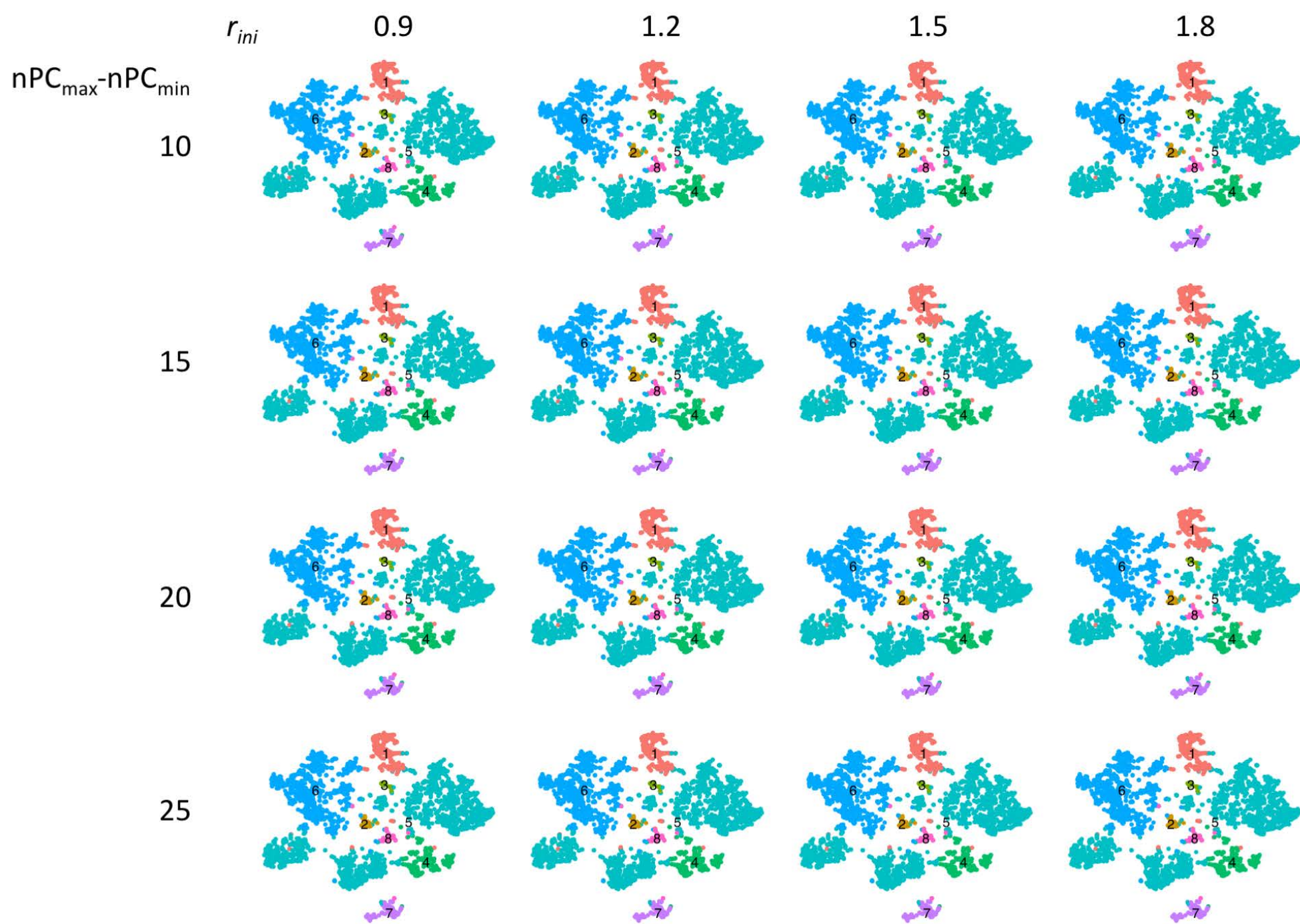

Supplement: giz121_Supplemental_Files [file giz121_supplemental_files.zip › Supplementary Figure17.pdf]

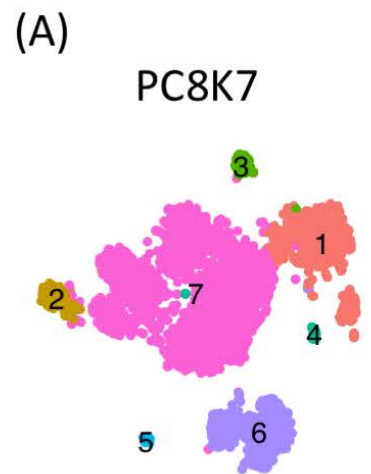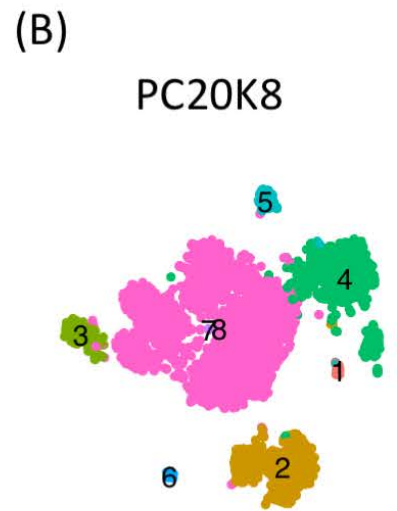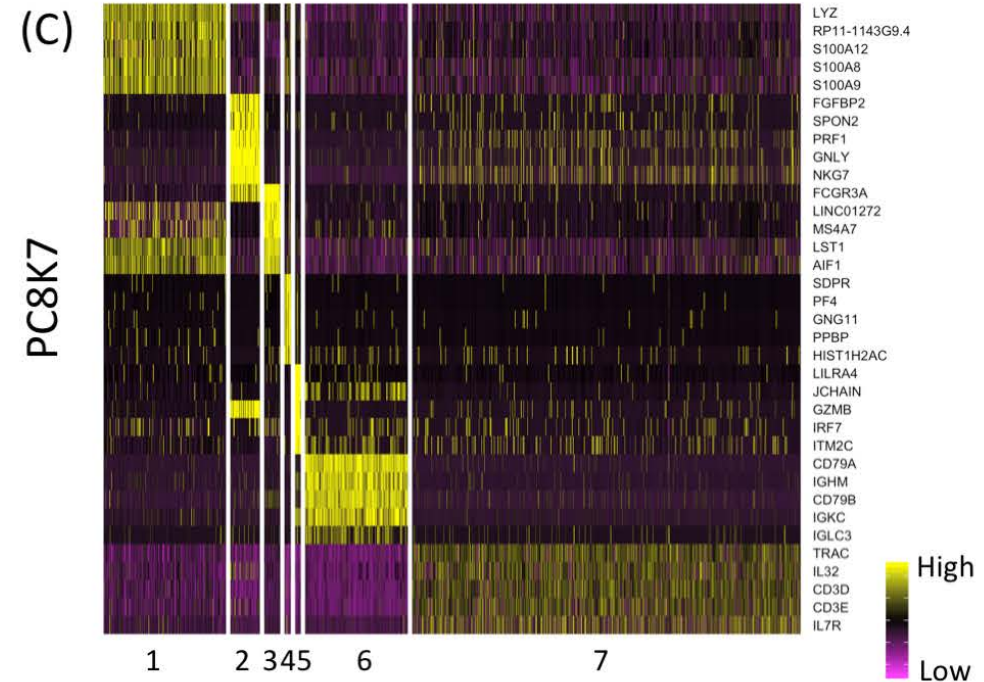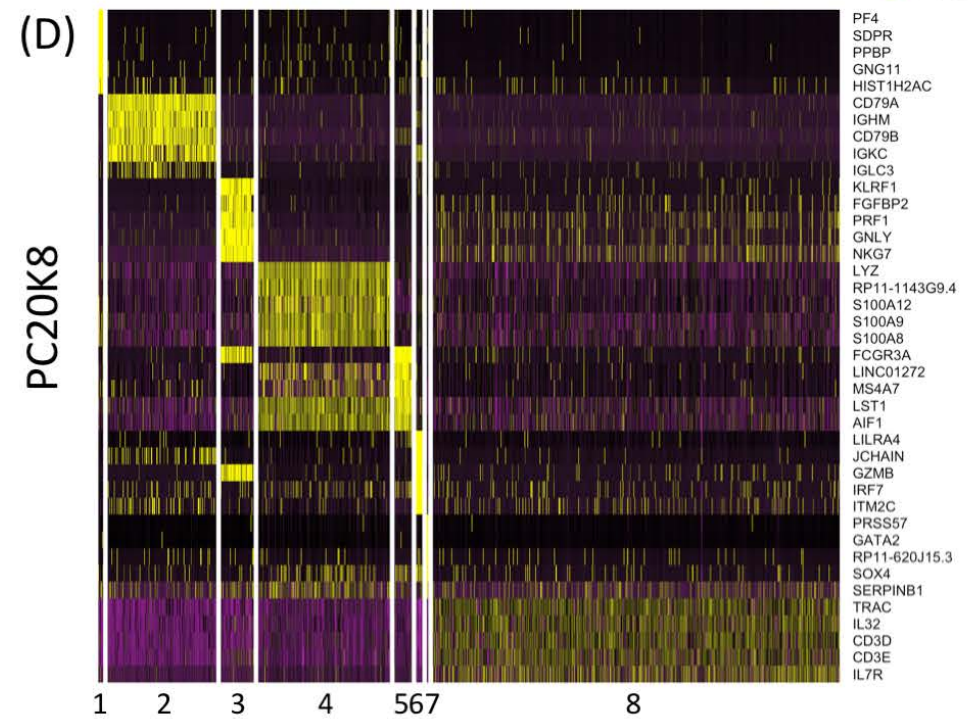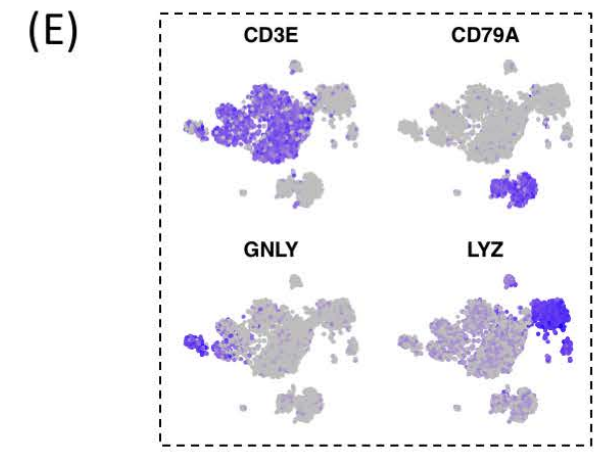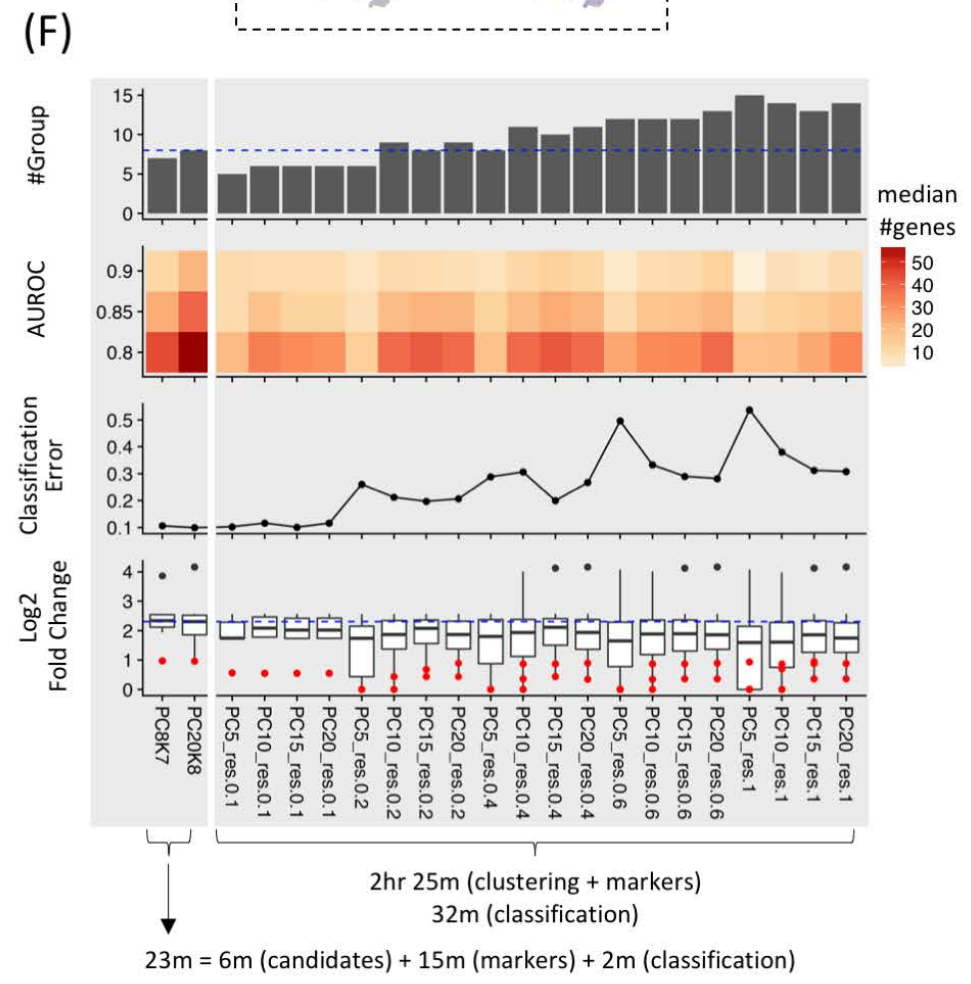

Supplement: giz121_Supplemental_Files [file giz121_supplemental_files.zip › Supplementary Figure5.pdf]

(A)

Original

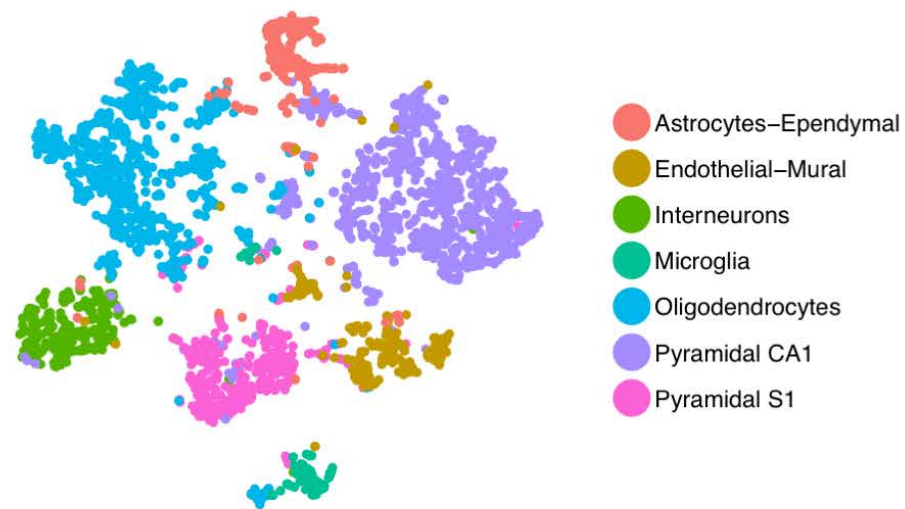

Modified

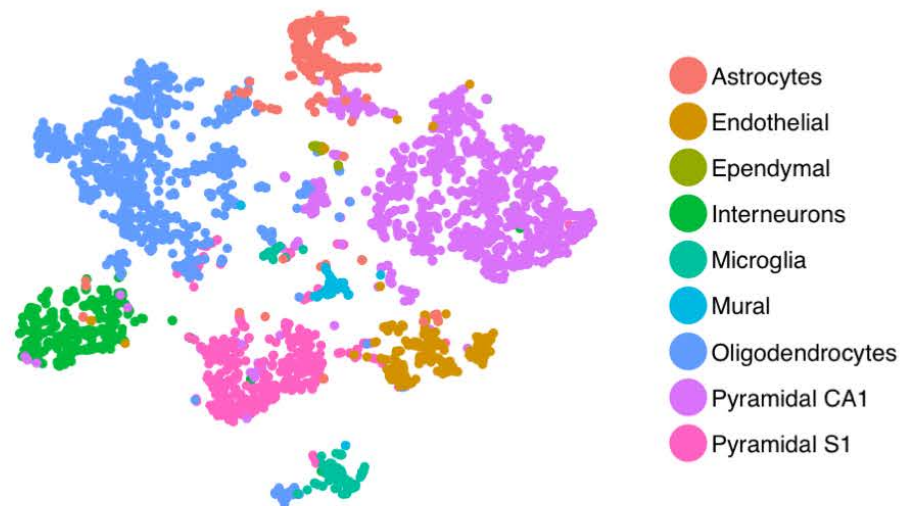

(B)

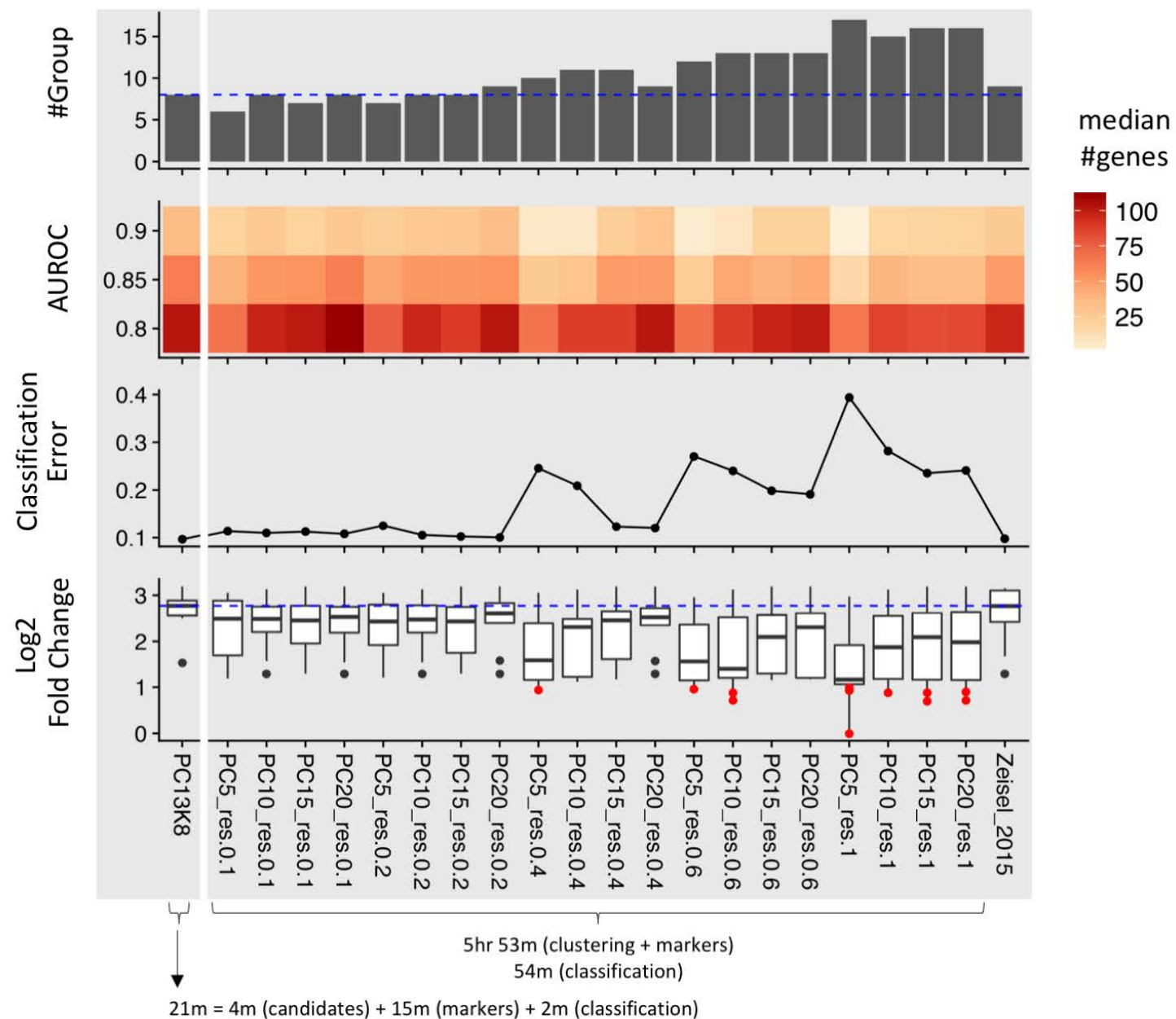

Supplement: giz121_Supplemental_Files [file giz121_supplemental_files.zip › Supplementary Figure8.pdf]

**Atp1a3**

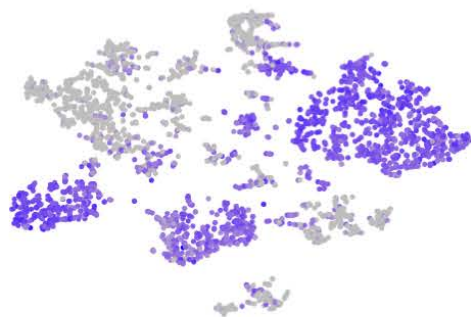

**Atp1b1**

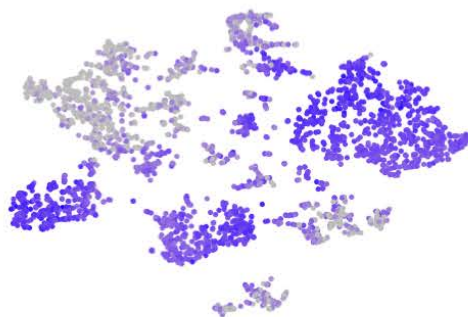

**Eno2**

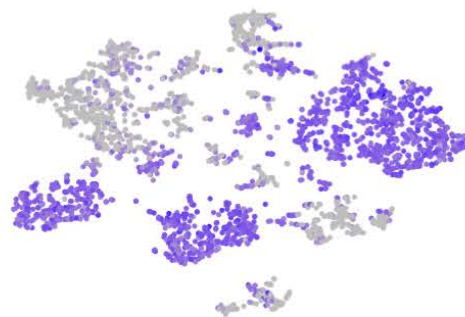

**Ndrg4**

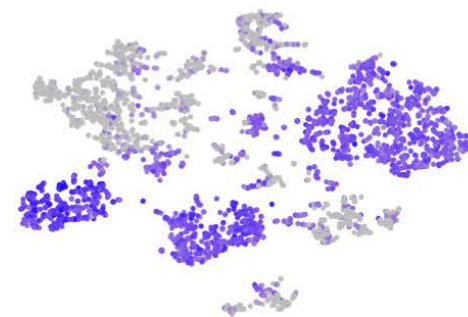

**Nsf**

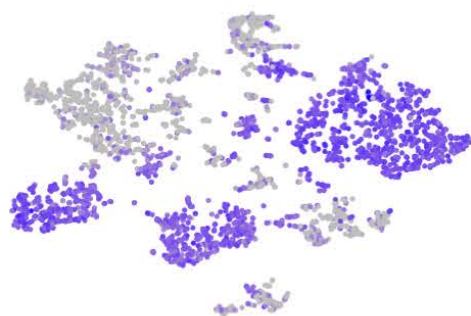

**Rab3a**

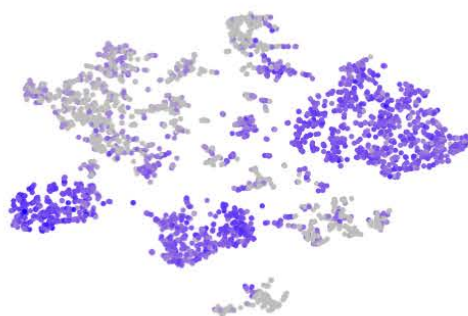

**Rtn1**

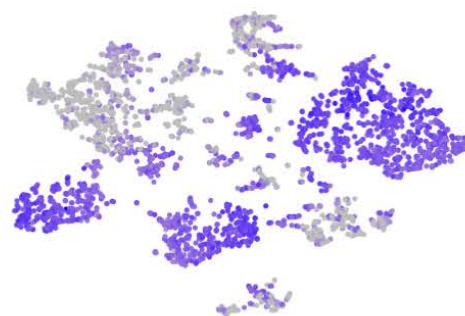

**Stmn3**

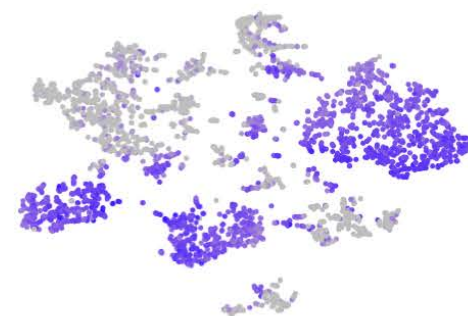

**Syp**

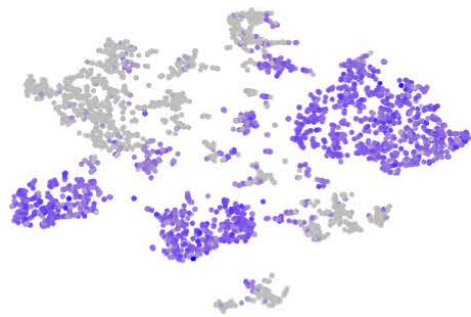

**Thy1**

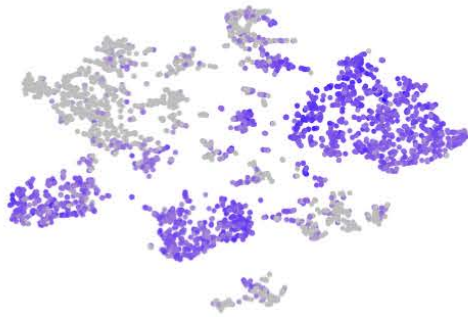

Supplement: giz121_Supplemental_Files [file giz121_supplemental_files.zip › Supplementary Figure9.pdf]
