# Supplementary material for: IKAP—Identifying K mAjor cell Population groups in single-cell RNA-sequencing analysis
Source: Gigascience. 2019 Oct 1;8(10):giz121. doi: 10.1093/gigascience/giz121 (PMC6771546; doi:10.1093/gigascience/giz121)
Supplement: giz121_Supplemental_Files [file Supplementary Figure 2.pdf › Supplementary Figure 2.pdf]

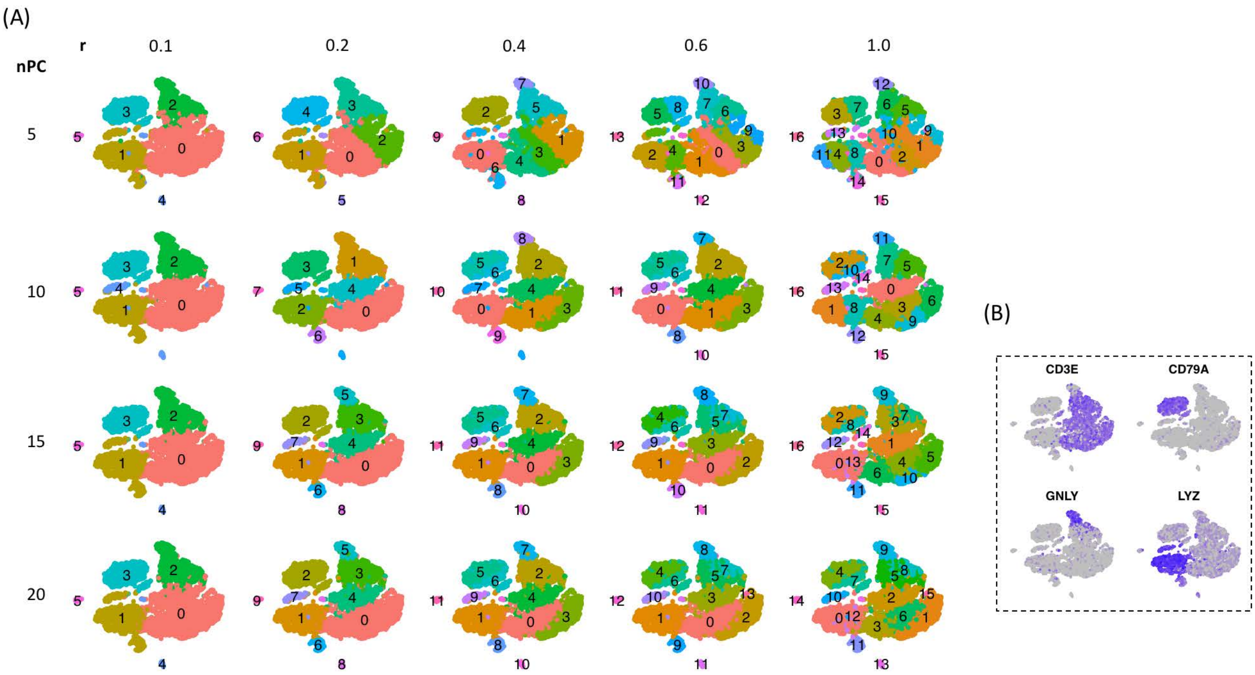

(A)
r
0.1
0.2
0.4
0.6
1.0
nPC
5
10
15
20
(B)
CD3E
CD79A
GNLY
LYZ
