# Supplementary material for: IKAP—Identifying K mAjor cell Population groups in single-cell RNA-sequencing analysis
Source: Gigascience. 2019 Oct 1;8(10):giz121. doi: 10.1093/gigascience/giz121 (PMC6771546; doi:10.1093/gigascience/giz121)
Supplement: giz121_Response_to_Reviewer_Comments_Original_Submission [file giz121_response_to_reviewer_comments_original_submission.pdf]

Author's Response To Reviewer Comments

Close

Reviewer reports:

Reviewer #1: A major challenge in single-cell RNA-seq data analysis is the gap between the 'clustering' and 'annotation' steps as pointed out by the authors. The first one is usually driven by feature selections and dimension reduction while the latter is done manually based on biological knowledge. The authors build on the existing clustering method called Seurat to propose a rational selection of a clustering that could approximate the 'best' choice in first instance. By optimizing these steps they want to improve the speed and quality of unsupervised clustering. The classification is evaluated by averaging over the expression values of the ten most differentially expressed genes (ranked by average log fold change) and calculating an error statistic. A decision tree is used to determine the candidate with the lowest classification error. To prove the efficacy of their workflow the authors reproduced the results of three publically available datasets from PBMCs and mouse cortex.

When applied to PBMC, the authors failed to identify T-cell subsets showing the limits of the approach (Figure 2). But, the recursive approach proposed in Figure 3 is very interesting and can be applied to find out more subsets.

I believe the approach is interesting for unknown datasets. Soon the multiplication of dataset annotated (notably in the large consortium like the human cell atlas) will allow to develop machine learning approaches to classify the cells automatically. The authors should discuss this point in the discussion.

What are the major claims of the paper?
The authors claim that by choosing a range of principle components and k values, calculating the clustering and ranking them by gap statistic they can identify the biologically most significant clusters and thus improve the overall quality of the unsupervised clustering as well as reducing the runtime.

Are they novel and will they be of interest to others in the community and the wider field?
The Evaluation of data clustering itself is not novel but it has not been applied for single-cell RNA-seq. Guidelines or algorithms for feature selection are needed and this approach completely removes the need to define the clustering parameters manually.

Is the work convincing, and if not, what further evidence would be required to strengthen the conclusions?
This approach is based on the assumption that clustering and differential gene expression analysis are coupled and no knowledge of the cell types is present. When compared to a more biologically driven workflow that discerns clusters based on canonical marker genes it will rather slow down than speed up the analysis.

On a more subjective note, do you feel that the paper will influence thinking in the field?
In my opinion, the authors are pushing the field into the right direction by evaluating the quality of feature selection and unsupervised clustering.

Ability of a researcher to reproduce the work, given the level of detail provided
The authors have provided a clear and structured GitHub repository that should allow full reproducibility and usability of their work and algorithm, respectively.

Response:
We thank the reviewer’s positive comments about IKAP. The reviewer finds the recursive approach

interesting and has suggested that we discuss about how this approach can be used to classify unknown datasets and how it is related to the growing number of annotated datasets such as the human cell atlas (the corresponding reviewer's comments are highlighted above). We have added relevant discussion in the revised manuscript (page 8, paragraph 2) and described our thoughts briefly below.

Because cell identities are hierarchical in nature (e.g. T cell subtypes within T cells), rather than classifying cells into a fixed number of annotated cell types at once, it would be more biologically meaningful to recover the full hierarchy of cell identities. Delineating the hierarchy of cell identities using scRNA-seq data can refine the existing cell ontology [1, 2] and help curate annotations for the reference database such as Human Cell Atlas [3]. Computationally, recovering the hierarchy is essentially a task that recursively identifies major groups as parent identities in the upper layer and finer groups within each major group as child identities in the next layer. In this study, we demonstrate that IKAP recovered the hierarchy of cell identities by constructing a multi-layered ontology for subsets of PBMC datasets and the mouse cortex dataset. Therefore, we believe IKAP can potentially help refine cell ontology and annotate the reference cell identities (such as Human Cell Atlas).

In this version, we have corrected a typo ("pyramidal SS" to "pyramidal S1") and changed the wording for clarity in the revised manuscript. We used differentially expressed (DE) genes for those used to differentiate computationally identified cell groups and reserved "marker genes" for those used to identify manually (or conventionally) annotated cell types. In addition, all major changes in this revised version are highlighted.

Reviewer #2: (NOTE TO READER: I'm using Markdown format below)

Reviewer report

Title: IKAP - Identifying K mAjor cell Population groups in single-cell RNA-seq analysis

Version: Original Submission

Date: June 13, 2019

Review comments to authors:

The authors present a method that elegantly tunes Seurat's clustering parameters to obtain cell clusters that have the best decision tree-classifiability. The method appears to be useful and fast, and by-and-large gives biologically sound results, especially in samples where cells of well-defined type can be expected. I do have some reservations about one of the cases (Mouse brain data by Zeisel et al. 2015) they chose to highlight. The latter may well have prompted the development of a recursive approach which appears to work well and is an interesting extension. I would recommend publication provided the major comments are addressed satisfactorily.

Response:
We thank the reviewer for the positive comments overall. We have revised the manuscript based on the reviewer's comments. All major changes are highlighted. We also provide our point-to-point responses below.

Major comments

* Fig. 2D: It is really unsatisfactory that three clearly separated groups (Interneurons, Pyramidal SS and Pyramidal CA1) are lumped together into one "Group 7". The authors should improve and/or explain this behaviour: is it due to their method (and/or Seurat FindAllMarkers) ending up choosing the wrong differentially expressed genes? If so, why? E.g., most of the Interneurons, Pyramidal SS and Pyramidal CA1 cells are perfectly recognizable in the heatmap (big yellow blocks), were it not for the Atp1a3, Ndrg4 and Stmn3 obscuring things. The latter 3 genes are probably selected based on their strong under-expression in groups 1 and 2. A number of things could be going on:
1. The decision tree approach yields a poor classifier that badly influences the choice of DE genes. It may not be much work to use a different classifier such as SVM, or even tweaking the parameters given to FindAllMarkers.

2. The authors developed and optimized the method based on the first big 10X Genomics data set, and

as a result the method does not generalize well enough. In my experience, the 10X data set is not so easy to classify. Most current cell type classification methods have difficulties with it, see Abdelaal et al. 2019, BioRxiv https://doi.org/10.1101/644435.). It could be remedied by applying their method to another data set, e.g. any of the cancer data sets from the Regev lab (with or without the malignant cells).

3. Confounding factors are at play. It might help to first filter out mitochondrial(ly-related) and ribosomal protein genes and all genes related to the cell cycle before doing anything else.

Response:
The reviewer is concerned that IKAP lumps the 3 cell types: interneurons, pyramidal CA1, and pyramidal S1 (please note that we corrected the typo "pyramidal SS" to "pyramidal S1" in the revision), together due to some unknown issues that need to be fixed because the 3 types are clearly separated groups with distinct marker genes. The reviewer also suspects that the 3 DE genes of the union group of the 3 cell types identified by IKAP were picked because of strong under-expression in group 1 and 2.

We thank the reviewer for providing insights on what could go wrong in the analysis. However, the union group identified by IKAP represents a high-level cell identity shared across interneurons, pyramidal CA1, and pyramidal S1. To support this, we have now added a supplementary figure in the revised manuscript showing that the top 10 genes with high AUROC that classify cells into the union group versus others are highly expressed in the 3 cell types but exhibit low (or no) expression in other types (Supplementary Figure 9). Then, we conducted Wilcoxon rank sum tests showing that all these 10 genes are significantly upregulated in each of the 3 cell types compared with each of the other cell types ($P < 1E-30$ for all tests). This shows that their differential expression was not simply due to strong under-expression in groups 1 and 2 (see Supplementary Table 1). In addition, these 3 cell types are also shown to be close to each other compared to other types in the clustering result in the original paper (see Figure 1C in [4]). These data support that although the 3 cell types expressed distinct marker genes, they also shared certain similarity in gene expression, implicating a potential common cell identity. We have added the analyses mentioned above in the main texts (starting from page 6, line 122).

The reviewer's comment also raises a key issue that it is not always clear as to what constitutes a cell type. For example, in the PBMC datasets, some people look for T cells whereas others look for T cell subtypes such as CD4+ and CD8+ T cells. All T cells express T cell marker genes but T cell subtypes are also differentiated by subtype marker genes. A similar case is seen for the union group versus the 3 cell types in the mouse cortex dataset. Because cell identities are hierarchical in nature, cell types can be presumably defined at different levels of resolution. To obtain the full picture of cell identities, we believe delineating the hierarchy of cell identities would be more biologically meaningful than classifying cells into a fixed number of reference cell types. Computationally, recovering the hierarchy is essentially a task that recursively identifies major groups as parent identities in the upper layer and finer groups within each major group as child identities in the next layer. We have shown that IKAP recovered the hierarchy of cell identities for subsets of PBMC and the mouse cortex datasets by recursively running it within each major group. We believe IKAP would be helpful for refining cell ontology and curating reference cell identities using scRNA-seq datasets in the future. We have added discussion for the points mentioned above in the main texts (page 8, paragraph 2).

To avoid ambiguity, in the revised manuscript, we use "cell type" for a group of cells that has been manually annotated and defined by a set of marker genes. This clarification has been added in page 5, line 86.

* The authors should point out (e.g. at the end of the Findings section) that their approach is only suitable for samples where a limited number of definite and distinct cell types can be expected. Their method forces cells into discrete groups, each with a maximally distinct gene expression profile. This will not work in cases with many unknown cells (e.g. tumor samples) or cases where gradients can be expected (time courses, developmental studies).

Response:
As suggested by the reviewer, we have added a paragraph that lists the concerns (including this one) when using IKAP (see page 9, paragraph 2).

* How were the AUROC's calculated? Details in the Performance Summary (page 13) are too few to reproduce.

Response:
We thank the reviewer for pointing out the missing detail. We have now added more details as follow: “For each DE gene, the AUROC (Area Under the ROC curve) was computed for classifying its associated group versus others using normalized UMI count and the function roc.curve in the R package PRROC.” (see page 13, line 268)

Suggestions

* From Suppl. Fig1 it would appear that testing each number of PC's is often superfluous, skipping a few could save time

* I strongly suggest making your package (also) compatible with Seurat 3.x, which is now the default and is likely to be used much more widely

Response:
We thank the reviewer’s suggestions about how to speed up IKAP and the compatibility of the latest Seurat package. We have already implemented a new version of IKAP compatible with Seurat v3. Now it is under the beta testing. We will release it as soon as the testing is completed. We are also compiling ideas to improve IKAP for our next release. This includes speeding up IKAP by skipping calculation of a few PCs.

Minor (editorial) comments:
* throughout the text:
* a quick read-through by a native speaker should help to fix the missing "the"'s.

Response:
We have fixed the issue as requested by the reviewer.

* the authors should clarify the distinction between differentially expressed (DE) genes (which are inferred, as here, between groups of cells) and marker genes, which are consensus genes whose products are conventionally used to identify cells. It appears that in most occurrences of this term, DE genes are meant, rather than marker genes.

Response:
As suggested by the reviewer, we have replaced “marker genes” with “DE genes” throughout the text when it is meant to be the genes differentially expressed between two computationally identified cell groups and clarified that marker genes are used to define a manually (or conventionally) annotated cell type (see page 5, line 86).

* the terms "feature list" and "choice of features" are in the machine-learning world known as "feature selection" and "feature set". Please change for clarity.

Response:
Changed as suggested by the reviewer.

* provide references for all the R packages used (those for rpart and PRROC are missing)

Response:
Both were added.

* Fig. 1, step 4, heatmap: biggest gap increase for nPC=9 visually appears to be for k=5->6, not for k=6->7 as indicated; perhaps adjust the color scale

* Fig. 2C: highlight the fact that chosen 3 parameters (3 leftmost columns) are contrasted with "trial-and-error strategy", e.g. by separating them horizontally a bit. Clarify that it need not be 3 candidates and one 'best'

* Fig. 2: what does the horizontal dashed line signify?

Response:
We thank the reviewer pointing out these details to improve the figures. We have adjusted Figures 1 and

2 and added description to the legend for Figure 2.

(Page numbers below refer to those in the reviewer document)

* page 6:
* "... two user-specified parameters: resolution r ..." This is a unclear, these are Seurat parameters which IKAP doesn't get, but infers itself.

Response:
We have changed the wording.

* " To simulate the coarse-to-fine grouping process ": should be "fine-to-coarse" as merging leads to ever-larger clusters

Response:
Changed.

* "For each set, gap statistic is" : insert "the". Authors should mention that Tibshirani's additional rule for selecting the 'best' k is not used here (why not?)

Response:
We have added “the”, mentioned that Tibshirani's rule of selecting the best k is not used (see page 12, line 231), and briefly explained why the rule is not used in the discussion (see page 9, line 169).

* "Finally, candidate sets grouped": clarify (apparently authors mean sets of k and nPC)

Response:
We have changed the wording to make it clear.

* reference [5]: incomplete reference, provide full download details

Response:
Added url to the download page.

* "marked as the best using decision trees" -> "marked as the best, using decision trees"

Response:
Changed.

* page 7:
* "such that cell lineage" -> "and cell lineage"

Response:
Changed.

* page 12:
* "each group in a candidate set": clarify what a candidate set is.

Response:
We have moved the original step 7 to step 4 in the revised version and made some changes in the Methods to make it clear (see page 12, step 4).

* page 13:
* "IKAP utilizes FindAllMarkers": insert "the"

Response:
Added.

* "is marked as the best and the rest are alternatives": How many 'alternatives' are there typically?

Response:
Based on the experiments we have tried, the number of candidate sets (including the best and the

alternatives) reported by IKAP usually range from 1 to 4. We have added this information in page 13, line 266.

Reference:

1. Aevermann BD, Novotny M, Bakken T, Miller JA, Diehl AD, Osumi-Sutherland D, et al. Cell type discovery using single-cell transcriptomics: implications for ontological representation. Hum Mol Genet. 2018;27(R1):R40-R7. doi:10.1093/hmg/ddy100
2. Bakken T, Cowell L, Aevermann BD, Novotny M, Hodge R, Miller JA, et al. Cell type discovery and representation in the era of high-content single cell phenotyping. BMC Bioinformatics. 2017;18(Suppl 17):559. doi:10.1186/s12859-017-1977-1
3. Regev A, Teichmann SA, Lander ES, Amit I, Benoist C, Birney E, et al. The Human Cell Atlas. Elife. 2017;6. doi:10.7554/eLife.27041
4. Zeisel A, Munoz-Manchado AB, Codeluppi S, Lonnerberg P, La Manno G, Jureus A, et al. Brain structure. Cell types in the mouse cortex and hippocampus revealed by single-cell RNA-seq. Science. 2015;347(6226):1138-42. doi:10.1126/science.aaa1934

Close
