# Supplementary material for: IKAP—Identifying K mAjor cell Population groups in single-cell RNA-sequencing analysis
Source: Gigascience. 2019 Oct 1;8(10):giz121. doi: 10.1093/gigascience/giz121 (PMC6771546; doi:10.1093/gigascience/giz121)
Supplement: giz121_Response_to_Reviewer_Comments_Revision_1 [file giz121_response_to_reviewer_comments_revision_1.pdf]

Author's Response To Reviewer Comments

Close

We thank the reviewer pointing out his latest work which shares the same idea with us. We are more than happy to cite his paper in our work and to see other people in the community pushing the field towards the same direction.

We also thank the reviewer picking up small details that need to be corrected but were missed by us. We have corrected them in this revision.

We will definitely speed up the release of the next version IKAP which is compatible with Seurat v3.

Close
