# Supplementary material for: IKAP—Identifying K mAjor cell Population groups in single-cell RNA-sequencing analysis
Source: Gigascience. 2019 Oct 1;8(10):giz121. doi: 10.1093/gigascience/giz121 (PMC6771546; doi:10.1093/gigascience/giz121)
Supplement: giz121_Reviewer_1_Report_Original_Submission — Antoine-Emmanuel Saliba -- 5/31/2019 Reviewed [file giz121_reviewer_1_report_original_submission.pdf]

Reviewer Report

Title: IKAP - Identifying K mAjor cell Population groups in single-cell RNA-seq analysis

Version: Original Submission **Date:** 5/31/2019

Reviewer name: Antoine-Emmanuel Saliba

Reviewer Comments to Author:

A major challenge in single-cell RNA-seq data analysis is the gap between the 'clustering' and 'annotation' steps as pointed out by the authors. The first one is usually driven by feature selections and dimension reduction while the latter is done manually based on biological knowledge. The authors build on the existing clustering method called Seurat to propose a rational selection of a clustering that could approximate the 'best' choice in first instance. By optimizing these steps they want to improve the speed and quality of unsupervised clustering. The classification is evaluated by averaging over the expression values of the ten most differentially expressed genes (ranked by average log fold change) and calculating an error statistic. A decision tree is used to determine the candidate with the lowest classification error. To prove the efficacy of their workflow the authors reproduced the results of three publically available datasets from PBMCs and mouse cortex.
When applied to PBMC, the authors failed to identify T-cell subsets showing the limits of the approach (Figure 2). But, the recursive approach proposed in Figure 3 is very interesting and can be applied to find out more subsets.
I believe the approach is interesting for unknown datasets. Soon the multiplication of dataset annotated (notably in the large consortium like the human cell atlas) will allow to develop machine learning approaches to classify the cells automatically. The authors should discuss this point in the discussion.
What are the major claims of the paper?
The authors claim that by choosing a range of principle components and k values, calculating the clustering and ranking them by gap statistic they can identify the biologically most significant clusters and thus improve the overall quality of the unsupervised clustering as well as reducing the runtime.
Are they novel and will they be of interest to others in the community and the wider field?
The Evaluation of data clustering itself is not novel but it has not been applied for single-cell RNA-seq. Guidelines or algorithms for feature selection are needed and this approach completely removes the need to define the clustering parameters manually.
Is the work convincing, and if not, what further evidence would be required to strengthen the conclusions?
This approach is based on the assumption that clustering and differential gene expression analysis are coupled and no knowledge of the cell types is present. When compared to a more biologically driven workflow that discerns clusters based on canonical markergenes it will rather slow down than speed up the analysis.
On a more subjective note, do you feel that the paper will influence thinking in the field?
In my opinion, the authors are pushing the field into the right direction by evaluating the quality of feature selection and unsupervised clustering.

Ability of a researcher to reproduce the work, given the level of detail provided
The authors have provided a clear and structured GitHub repository that should allow full reproducibility and usability of their work and algorithm, respectively.

Level of Interest

Please indicate how interesting you found the manuscript: Choose an item.

Quality of Written English

Please indicate the quality of language in the manuscript: Choose an item.

Declaration of Competing Interests

Please complete a declaration of competing interests, considering the following questions:

- Have you in the past five years received reimbursements, fees, funding, or salary from an organisation that may in any way gain or lose financially from the publication of this manuscript, either now or in the future?
- Do you hold any stocks or shares in an organisation that may in any way gain or lose financially from the publication of this manuscript, either now or in the future?
- Do you hold or are you currently applying for any patents relating to the content of the manuscript?
- Have you received reimbursements, fees, funding, or salary from an organization that holds or has applied for patents relating to the content of the manuscript?
- Do you have any other financial competing interests?
- Do you have any non-financial competing interests in relation to this paper?

If you can answer no to all of the above, write 'I declare that I have no competing interests' below. If your reply is yes to any, please give details below.

I declare that I have no competing interests

I agree to the open peer review policy of the journal. I understand that my name will be included on my report to the authors and, if the manuscript is accepted for publication, my named report including any attachments I upload will be posted on the website along with the authors' responses. I agree for my report to be made available under an Open Access Creative Commons CC-BY license (http://creativecommons.org/licenses/by/4.0/). I understand that any comments which I do not wish to be included in my named report can be included as confidential comments to the editors, which will not be published.

Choose an item.

To further support our reviewers, we have joined with Publons, where you can gain additional credit to further highlight your hard work (see: https://publons.com/journal/530/gigascience). On publication of this paper, your review will be automatically added to Publons, you can then choose whether or not to claim your Publons credit. I understand this statement.

Yes Choose an item.
