# Supplementary material for: IKAP—Identifying K mAjor cell Population groups in single-cell RNA-sequencing analysis
Source: Gigascience. 2019 Oct 1;8(10):giz121. doi: 10.1093/gigascience/giz121 (PMC6771546; doi:10.1093/gigascience/giz121)
Supplement: giz121_Reviewer_2_Report_Original_Submission — Philip Lijnzaad -- 6/13/2019 Reviewed [file giz121_reviewer_2_report_original_submission.pdf]

Reviewer Report

Title: IKAP - Identifying K mAjor cell Population groups in single-cell RNA-seq analysis

Version: Original Submission **Date:** 6/13/2019

Reviewer name: Philip Lijnzaad

Reviewer Comments to Author:

(NOTE TO READER: I'm using Markdown format below)
Reviewer report
Title: IKAP - Identifying K mAjor cell Population groups in single-cell RNA-seq analysis
Version: Original Submission
Date: June 13, 2019
Review comments to authors:
The authors present a method that elegantly tunes Seurat's clustering parameters to obtain cell clusters that have the best decision tree-classifiability. The method appears to be useful and fast, and by-and-large gives biologically sound results, especially in samples where cells of well-defined type can be expected. I do have some reservations about one of the cases (Mouse brain data by Zeisel et al. 2015) they chose to highlight. The latter may well have prompted the development of a recursive approach which appears to work well and is an interesting extension. I would recommend publication provided the major comments are addressed satisfactorily.
Major comments
* Fig. 2D: It is really unsatisfactory that three clearly separated groups (Interneurons, Pyramidal SS and Pyramidal CA1) are lumped together into one "Group 7". The authors should improve and/or explain this behaviour: is it due to their method (and/or Seurat FindAllMarkers) ending up choosing the wrong differentially expressed genes? If so, why? E.g., most of the Interneurons, Pyramidal SS and Pyramidal CA1 cells are perfectly recognizable in the heatmap (big yellow blocks), were it not for the Atp1a3, Ndrg4 and Stmn3 obscuring things. The latter 3 genes are probably selected based on their strong under-expression in groups 1 and 2. A number of things could be going on:
  1. The decision tree approach yields a poor classifier that badly influences the choice of DE genes. It may not be much work to use a different classifier such as SVM, or even tweaking the parameters given to FindAllMarkers.
  2. The authors developed and optimized the method based on the first big 10X Genomics data set, and as a result the method does not generalize well enough. In my experience, the 10X data set is not so easy to classify. Most current cell type classification methods have difficulties with it, see Abdelaal et al. 2019, BioRxiv https://doi.org/10.1101/644435.). It could be remedied by applying their method to another data set, e.g. any of the cancer data sets from the Regev lab (with or without the malignant cells).
  3. Confounding factors are at play. It might help to first filter out mitochondrial(ly-related) and ribosomal protein genes and all genes related to the cell cycle before doing anything else.
* The authors should point out (e.g. at the end of the Findings section) that their approach is only

suitable for samples where a limited number of definite and distinct cell types can be expected. Their method forces cells into discrete groups, each with a maximally distinct gene expression profile. This will not work in cases with many unknown cells (e.g. tumor samples) or cases where gradients can be expected (time courses, developmental studies).
* How were the AUROC's calculated? Details in the Performance Summary (page 13) are too few to reproduce.
# Suggestions
* From Suppl. Fig1 it would appear that testing each number of PC's is often superfluous, skipping a few could save time
* I strongly suggest making your package (also) compatible with Seurat 3.x, which is now the default and is likely to be used much more widely
# Minor (editorial) comments:
* throughout the text:
  * a quick read-through by a native speaker should help to fix the missing "the"'s.
  * the authors should clarify the distinction between differentially expressed (DE) genes (which are inferred, as here, between groups of cells) and marker genes, which are consensus genes whose products are conventionally used to identify cells. It appears that in most occurrences of this term, DE genes are meant, rather than marker genes.
  * the terms "feature list" and "choice of features" are in the machine-learning world known as "feature selection" and "feature set". Please change for clarity.
  * provide references for all the R packages used (those for rpart and PRROC are missing)
* Fig. 1, step 4, heatmap: biggest gap increase for nPC=9 visually appears to be for k=5->6, not for k=6->7 as indicated; perhaps adjust the color scale
* Fig. 2C: highlight the fact that chosen 3 parameters (3 leftmost columns) are contrasted with "trial-and-error strategy", e.g. by separating them horizontally a bit. Clarify that it need not be 3 candidates and one 'best'
* Fig. 2: what does the horizontal dashed line signify?
(Page numbers below refer to those in the reviewer document)
* page 6:
  * "... two user-specified parameters: resolution r ..." This is a unclear, these are Seurat parameters which IKAP doesn't get, but infers itself.
  * " To simulate the coarse-to-fine grouping process ": should be "fine-to-coarse" as merging leads to ever-larger clusters
  * "For each set, gap statistic is" : insert "the". Authors should mention that Tibshirani's additional rule for selecting the 'best' k is not used here (why not?)
  * "Finally, candidate sets grouped": clarify (apparently authors mean sets of k and nPC)
  * reference [5]: incomplete reference, provide full download details
  * "marked as the best using decision trees" -> "marked as the best, using decision trees"
* page 7:
  * "such that cell lineage" -> "and cell lineage"
* page 12:
  * "each group in a candidate set": clarify what a candidate set is.

* page 13:
  * "IKAP utilizes FindAllMarkers": insert "the"
  * "is marked as the best and the rest are alternatives": How many 'alternatives' are there typically?

Level of Interest

Please indicate how interesting you found the manuscript: Choose an item.

Quality of Written English

Please indicate the quality of language in the manuscript: Choose an item.

Declaration of Competing Interests

Please complete a declaration of competing interests, considering the following questions:

- Have you in the past five years received reimbursements, fees, funding, or salary from an organisation that may in any way gain or lose financially from the publication of this manuscript, either now or in the future?
- Do you hold any stocks or shares in an organisation that may in any way gain or lose financially from the publication of this manuscript, either now or in the future?
- Do you hold or are you currently applying for any patents relating to the content of the manuscript?
- Have you received reimbursements, fees, funding, or salary from an organization that holds or has applied for patents relating to the content of the manuscript?
- Do you have any other financial competing interests?
- Do you have any non-financial competing interests in relation to this paper?

If you can answer no to all of the above, write 'I declare that I have no competing interests' below. If your reply is yes to any, please give details below.

I declare that I have no competing interests.

I agree to the open peer review policy of the journal. I understand that my name will be included on my report to the authors and, if the manuscript is accepted for publication, my named report including any attachments I upload will be posted on the website along with the authors' responses. I agree for my report to be made available under an Open Access Creative Commons CC-BY license (http://creativecommons.org/licenses/by/4.0/). I understand that any comments which I do not wish to

be included in my named report can be included as confidential comments to the editors, which will not be published.

Choose an item.

To further support our reviewers, we have joined with Publons, where you can gain additional credit to further highlight your hard work (see: https://publons.com/journal/530/gigascience). On publication of this paper, your review will be automatically added to Publons, you can then choose whether or not to claim your Publons credit. I understand this statement.

Yes Choose an item.
